# Supplementary material for: Synthesis and characterization of a formal 21-electron cobaltocene derivative
Source: Nat Commun. 2023 Sep 5;14:4979. doi: 10.1038/s41467-023-40557-7 (PMC10480225; doi:10.1038/s41467-023-40557-7)
Supplement: Supplementary file 1 — Supplementary Information [file 41467_2023_40557_MOESM1_ESM.pdf]

Supplementary Information for

Synthesis and characterization of a formal 21-electron cobaltocene derivative

Satoshi Takebayashi, Jama Ariai, Urs Gellrich, Sergey V. Kartashov, Robert R. Fayzullin, Hyung-Been Kang, Takeshi Yamane, Kenji Sugisaki, and Kazunobu Sato

Correspondence to: satoshi.takebayashi@oist.jp, urs.gellrich@org.chemie.uni-giessen.de, robert.fayzullin@gmail.com

Table of Contents

1. Preparation of ligands and complexes 2

2. NMR, IR and EPR spectra and VSM data 9

3. EPR studies 36

4. Quantum crystallography and SC-XRD studies 43

5. DFT studies 75

6. Supplementary references 85

1. Preparation of ligands and complexes

Preparation of 2,6-bis(methylenecyclopentadienyl)pyridine disodium salt (Na_2CpNCp)

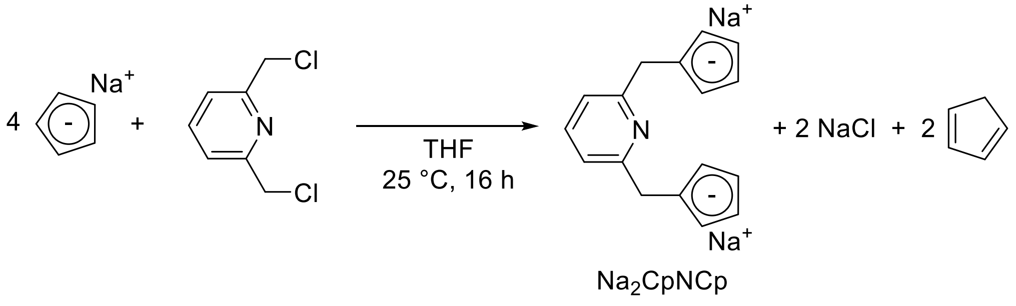

2,6-bis(methylenecyclopentadienyl)pyridine disodium salt, Na_2CpNCp, was prepared according to a published method[1]. A 20 mL vial equipped with a Teflon coated stirring bar was charged with NaCp (5.37 mL, 1.49 M THF solution, 8.00 mmol). 2,6-bis(chloromethyl)pyridine (352.0 mg, 2.00 mmol) in 4 mL THF was added to the vial dropwise for about 5 min. The solution warmed up to about 50 °C due to the exothermic reaction and white precipitate of NaCl formed. The mixture was stirred for 17 h at 25 °C. The resulting yellow solution and white precipitate was filtered using a plug of Celite. The Celite was washed three times with 1 mL each of THF, and the filtered yellow solution was concentrated under vacuum. White crystalline solid of the product formed on the glass wall upon concentration to about 2 mL. More product precipitated from the concentrated solution upon storing the solution at -35 °C for overnight. The red-orange supernatant was decanted and the remaining solid was washed three times with cold diethyl ether to remove unreacted NaCp and orange colored material. The resulting white solid was dried overnight under high vacuum (< 0.2 mmHg) to remove coordinated THF molecules. More product was obtained by repeating the precipitation procedure using the concentrated supernatant liquid. Total yield: 349.7 mg, 63%. ^{1}H NMR data in THF-d_8 agrees with the previous reports [1]. Previously not reported ^{13}C{^{1}H}, and ^{15}N NMR data and ^{1}H NMR data in CD_3CN and in THF-d_8 are reported. ^{1}H and ^{13}C{^{1}H}NMR spectrum in CD_3CN showed signals due to partial deuteration of cyclopentadienyl protons during NMR data collection, thus only ^{13}C{^{1}H}NMR chemical shifts of the major isotopologue are reported in CD_3CN.

Supplementary Figs. 1-3 shows ^{1}H, ^{13}C{^{1}H}, and ^{1}H-^{15}N HMBC NMR spectra of Na_2CpNCp.
^{1}H NMR (500.13 MHz, CD_3CN, 298 K): δ 3.91 (4H, s, 2 CH_2), 5.25 (4H, m, 2,5-position of C_5H_4 groups), 5.46 (4H, m, 3,4-position of C_5H_4 groups), 6.97 (2H, d, $^{3}J_{HH}$, = 7.6 Hz, 3,5-position of pyridine ring), 7.49 (1H, d, $^{3}J_{HH}$, = 7.6 Hz, 4-position of pyridine ring).
^{1}H NMR (500.13 MHz, THF-d_8, 298 K): δ 3.93 (4H, s, 2 CH_2), 5.34 (4H, pseudo t, 2,5-position of C_5H_4 groups), 5.52 (4H, pseudo t, 3,4-position of C_5H_4 groups), 6.89 (2H, d, $^{3}J_{HH}$, = 7.5 Hz, 3,5-position of pyridine ring), 7.40 (1H, d, $^{3}J_{HH}$, = 7.5 Hz, 4-position of pyridine ring).
^{13}C{^{1}H} NMR (125.76 MHz, CD_3CN, 298 K): δ 39.5 (s, CH_2), 103.7 (s, 3,4-position of C_5H_4 groups), 104.5 (s, 2,5-position of C_5H_4 groups), 116.2 (s, 1-position of C_5H_4 groups), 119.9 (s, 3,5-position of pyridine ring), 136.9 (s, 4-position of pyridine ring), 166.4 (s, 2,6-position of pyridine ring).
^{13}C{^{1}H} NMR (125.76 MHz, THF-d_8, 298 K): δ 39.7 (s, CH_2), 103.2 (s, 3,4-position of C_5H_4 groups), 104.3 (s, 2,5-position of C_5H_4 groups), 115.3 (s, 1-position of C_5H_4 groups), 119.6 (s, 3,5-

position of pyridine ring), 136.2 (s, 4-position of pyridine ring), 166.1 (s, 2,6-position of pyridine ring).
^{15}N NMR (50.68 MHz, CD_3CN, 298 K, detected using ^{1}H-^{15}N HMBC): δ 297.5 (s).
^{15}N NMR (50.68 MHz, THF-d_8, 298 K, detected using ^{1}H-^{15}N HMBC): δ 297.3 (s).

Preparation of [Co(CpNCp)] (**1**)

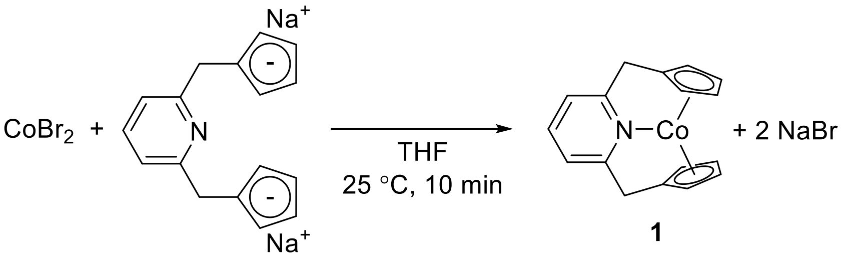

In a nitrogen glovebox, a 20 mL vial equipped with a Teflon coated stirring bar was charged with $CoBr_2$ (245.3 mg, 1.12 mmol) and 4 mL THF. The solution was stirred at 25 °C until clear blue solution was obtained. To the solution was then added a solid of Na_2CpNCp (250.4 mg, 0.897 mmol) portionwise for ca. 2 min. As soon as addition of Na_2CpNCp was completed, 10 mL n-pentane was added to the solution to precipitate purple solid of byproducts, and the red-orange solution was filtered using plug of Celite, and the Celite was washed by 1:1 THF:pentane mixture. Concentration of combined solution gave red-orange crystals of **1**. Yield: 88.3 mg, 34%. This procedure was repeated another time using $CoBr_2$ (253.6 mg, 1.16 mmol) and Na_2(CpNCp), (247.1 mg, 0.885 mmol), and 88.9 mg of **1** (34% yield) was obtained.
Supplementary Figs. 4-9 shows ^{1}H, ^{13}C{^{1}H}, ^{1}H-^{1}H COSY and ^{1}H-^{13}C HSQC NMR, FTIR, and VSM data of **1**.
^{1}H NMR (400.15 MHz, C_6D_6, 298 K): δ -260.2 (4H, $\Delta\nu^{1/2}$ = 1324.7 Hz, a signal from 3,4-position of C_5H_4 groups), -187.5 (4H, $\Delta\nu^{1/2}$ = 873.5 Hz, a signal from 2,5-position of C_5H_4 groups), 24.7 (1H, $\Delta\nu^{1/2}$ = 25.9 Hz, a signal from 4-position of pyridine ring), 74.9 (2H, $\Delta\nu^{1/2}$ = 49.6 Hz, a signal from 3,5-position of pyridine ring), 144.3 (4H, $\Delta\nu^{1/2}$ = 164.2 Hz, 2 CH_2).
^{13}C NMR (proton coupled) (125.76 MHz, C_6D_6, 298 K): δ -503.8 (br), 122.7 (br), 251.6 (d, $^{1}J_{CH}$ = 159.7 Hz, a signal from 4-position of pyridine ring), 409.7 (d, $^{1}J_{CH}$ = 159.7 Hz, a signal from 3,5-position of pyridine ring). Three ^{13}C NMR signals are missing likely due to direct bonding of cyclopentadienyl group to paramagnetic Co.
Effective magnetic moment: μ_{eff} (Evans' method, C_6D_6, 298.1 K) = 4.3 μ_B. μ_{eff} (VSM, 298.17 K) = 3.99 μ_B, Weiss constant (θ) = -7.69 K.
FTIR (Thin film, cm^{-1}): 1568 (w, C=C and C=N stretch), 1451 (m, sp^3 C-H bending), 1413 (m, sp^3 C-H bending), 1023 (m, sp^2 C-H bending), 758 (s, sp^2 C-H bending).
EPR (9.079 GHz, 2.5 mM toluene glass, 4.2 K): g_1, g_2, g_3 = 5.077, 3.172, 1.896. A_1, A_2, A_3 (^{59}Co) = 205, 142, 293 MHz.
Elemental analysis: Calcd for $C_{17}H_{15}NCo$: C:69.87, H:5.17, N:4.79. Found: C:69.93, H:5.21, N:4.80.
HRMS (ESI/Orbitrap, $[M]^+$): Calcd for $C_{17}H_{15}NCo$: 292.0531. Found: 292.0528.

Preparation of partially deuterated [Co(CpNCp)]-d_8 (**1-*d*₈**)

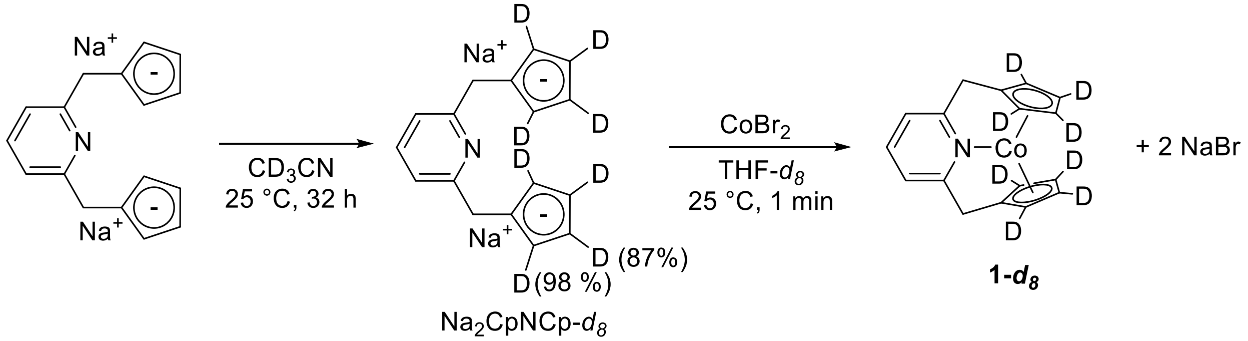

In a nitrogen glovebox, a J. Young NMR tube was charged with Na_2CpNCp (3.6 mg, 0.013 mmol) and 0.5 mL dry CD_3CN. The clear solution was then kept at 25 °C, and progress of deuteration of cyclopentadienyl moiety was monitored by ^1H NMR. The deuteration of 2,5-position was faster than 3,4-position. Thus 2,5-position was deuterated 83%, whereas 3,4-position was deuterated 44% after 9 h. After 32 h, 2,5-position and 3,4-position of cyclopentadienyl groups were deuterated 98% and 87%, respectively. After 32 h, CD_3CN solvent was removed under vacuum and the product was dissolved in 0.5 mL dry THF-d_8. After this procedure extent of deuteration was changed slightly to 95% and 90% for 2,5-position and 3,4-position of cyclopentadienyl groups, respectively. To the solution was added a solid of $CoBr_2$ (3.4 mg, 0.016 mmol). The solution was shaken 1 min to obtain purple solution of crude product mixture. ^1H NMR of the crude mixture showed formation of **1-*d*₈** with deuteration of signals at -260.5 and -187.7 ppm. ^1H signals from cyclopentadienyl groups were assigned based on relative integration values of signals at -260.5 (3,4-position) and -187.7 (2,5-position) ppm, which was 0.67:1. Detection of deuterated signals by ^2H NMR was not successful likely due to lower sensitivity of ^2H NMR and broadening of signals at -260.5 and -187.7 ppm.

Supplementary Figs. 10-11 shows ^1H NMR and ^2H NMR spectrum of Na_2CpNCp-d_8. Supplementary Fig.12 shows ^1H NMR spectra of partially deuterated **1-*d*₈** used for assignment of ^1H NMR signals from cyclopentadienyl groups.

Preparation of [Co(CpNCp)]-d_8 (**1-*d*₈**)

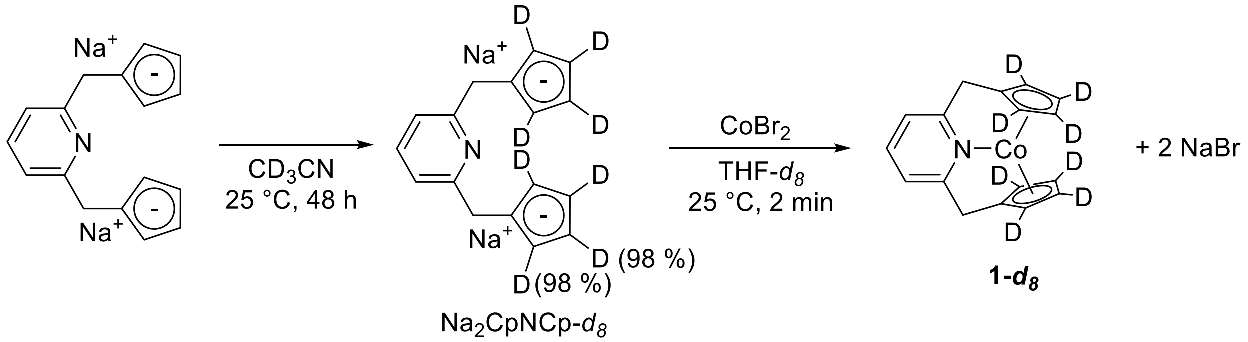

In a nitrogen glovebox, a 20 mL vial equipped with a stirring bar was charged with Na_2CpNCp (70.0 mg, 0.250 mmol) and 2.0 mL dry CD_3CN. The dark clear solution was then stirred at 25 °C for 48 h. After 48 h, aliquot of the solution was analyzed by ^1H NMR. ^1H NMR

showed formation of Na_2CpNCp-d_8 with 98% deuteration at 2,5- and 3,4-positions of cyclopentadienyl groups.
HRMS (ESI/Orbitrap, $[M + 3H^+ - 2Na^-]^+$): Calcd for $C_{17}H_{10}D_8N$: 244.1936. Found: 244.1936.

CD_3CN solvent was removed under vacuum and the product was dissolved in 2 mL THF. Complete dissolution was not achieved and hence the solution was use as a suspension. In another 20 mL vial equipped with a stirring bar was added $CoBr_2$ (68.3 mg, 0.312 mmol) and 2 mL THF. A clear blue solution of $CoBr_2$ formed on stirring. To the stirred solution was added a suspension of $Na_2(CpNCp)$-d_8 dropwise for ca. 2 min. The vial containing $Na_2(CpNCp)$-d_8 was washed 3 times with 0.5 mL each of THF and the THF solution was added to the $CoBr_2$ solution. As soon as addition of $Na_2(CpNCp)$-d_8 was completed, 10 mL n-pentane was added to the solution to precipitate purple solid of byproducts, and the red-orange solution was filtered using plug of Celite, and the Celite was washed by 1:1 THF:pentane mixture. Concentration of combined solution gave red-orange crystals of **1-*d*$_8$**. Yield: 36.4 mg, 48%.
Supplementary Fig. 13 shows ^{1}H NMR spectra of **1-*d*$_8$**.
^{1}H NMR (400.15 MHz, C_6D_6, 298 K): δ 24.6 (1H, $\Delta\nu^{1/2}$ = 20.2 Hz, a signal from 4-position of pyridine ring), 74.6 (2H, $\Delta\nu^{1/2}$ = 44.4 Hz, a signal from 3,5-position of pyridine ring), 144.2 (4H, $\Delta\nu^{1/2}$ = 165.1 Hz, 2 CH_2).
HRMS (ESI/Orbitrap, $[M]^+$): Calcd for $C_{17}H_7D_8NCo$: 300.1033. Found: 300.1033.

<u>Preparation of $[Ni(CpNCp)]_2$ (**2**)</u>

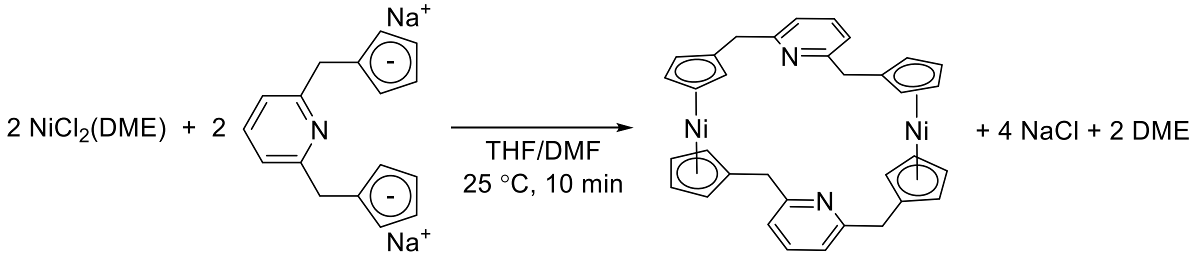

2

In a nitrogen glovebox, a 20 mL vial equipped with a Teflon coated stirring bar was charged with $CoCl_2$(DME) (44.6 mg, 0.203 mmol) and 4 mL DMF. The solution was stirred at 25 °C until clear solution was obtained. To the solution was then added dropwise Na_2(CpNCp) (56.3 mg, 0.202 mmol) in 8 mL THF. The mixture was stirred for 30 min at 25 °C, and green solution and grey precipitate was obtained. The solution was filtered using plug of Celite, and the Celite was washed by THF. The combined solution was concentrated to dryness at 25 °C using high vacuum (< 0.2 mmHg). The resulting green oil was extracted using 1:4 THF-pentane mixture. Concentration of the solution gave green solid of **2**. Yield: 35.1 mg, 60%.
Supplementary Figs. 14-17 shows ^{1}H, ^{13}C{^{1}H}, and ^{1}H-^{13}C HSQC NMR, and FTIR spectra of **2**.
^{1}H NMR (400.15 MHz, THF-d_8, 298 K): δ -253.3 (8H, $\Delta\nu^{1/2}$ = 1230.5 Hz, a signal from C_5H_4 groups due to overlapping of ^{1}H signals from 2,5- and 3,4-positions), -187.7 (4H, $\Delta\nu^{1/2}$ = 873.5 Hz, a signal from 2,5-position of C_5H_4 groups), 5.05 (2H, $\Delta\nu^{1/2}$ = 88.4 Hz, a signal from 3,5-position of pyridine ring), 8.59 (1H, $\Delta\nu^{1/2}$ = 21.7 Hz, a signal from 4-position of pyridine ring), 196.7 (4H, $\Delta\nu^{1/2}$ = 610.5 Hz, 2 CH_2).
^{13}C NMR (proton coupled) (125.76 MHz, THF-d_8, 298 K): δ -559.3 (br), 158.10 (d, $^1J_{CH}$ = 160.4 Hz, a signal from 4-position of pyridine ring), 171.9 (d, $^1J_{CH}$ = 159.1 Hz, a signal from 3,5-position

of pyridine ring). Four ^{13}C NMR signals are missing likely due to proximity to the paramagnetic Co.
Effective magnetic moment: μ_{eff} (Evans' method, THF-d_8, 298 K) = 2.8 μ_B/Ni.
FTIR (Thin film, cm^{-1}): 1587 (m, C=C and C=N stretch), 1575 (m, C=C and C=N stretch), 1454 (m, sp^3 C-H bending), 1429 (m, sp^3 C-H bending), 1037 (m, sp^2 C-H bending), 1022 (m, sp^2 C-H bending), 768 (m, sp^2 C-H bending).
EPR (9.077 GHz, toluene glass, 77.4 K): No signal detected due to S = 1, non-Kramer system.
Elemental analysis: Calcd: C:69.93, H:5.18, N:4.80. Found: C:70.29, H:5.01, N:4.54.
HRMS (ESI/Orbitrap, [M]$^+$): Calcd for $C_{34}H_{30}N_2Ni_2$: 582.1110. Found: 582.1105

Formation of [Co(CpNCp)]BF_4 (**3**) by oxidation of **1**

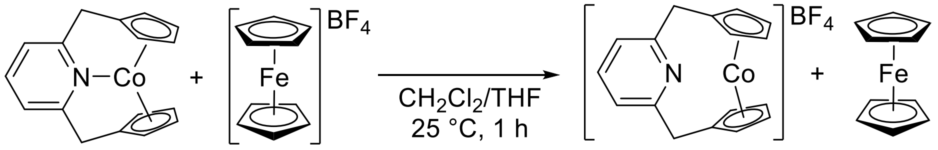

1 **3**

In a nitrogen glovebox, a 20 mL vial equipped with a Teflon coated stirring bar was charged with **1** (14.6 mg, 0.050 mmol), ferrocenium tetrafluoroborate (14.3 mg, 0.052 mmol) and 2 mL THF and 4 mL CH_2Cl_2. The solution was stirred at 25 °C for 10 min, and dark purple solution was obtained. The solution was filtered using plug of Celite, and the Celite was washed by CH_2Cl_2. The combined solution was concentrated to dryness, and a mixture of dark purple crystal of **3** and orange crystal of ferrocene was obtained. The solid was washed three times with diethyl ether, and then three times with THF and dried under vacuum. Yield: 18.2 mg, 100%. Broadening of ^{1}H signals or increase of effective magnetic moment due to formation of Co-N bond were not observed between -40 and 80 °C.
Supplementary Figs. 18-23 shows ^{1}H, ^{13}C{^{1}H}, ^{1}H-^{15}N HMBC, ^{19}F, and ^{11}B NMR, and FTIR spectra of **3**. Supplementary Fig. 24 shows comparison of ^{1}H NMR spectra recorded at variable temperatures.
^{1}H NMR (500.13 MHz, CD_3CN, 298 K): δ 3.31 (4H, s, 2 CH_2), 5.61 (4H, pseudo t with roofing, $^3J_{HH}$, $^4J_{HH}$ = 2.0, 2.4 Hz, 3,4-position of C_5H_4 groups), 5.64 (4H, pseudo t with roofing, $^3J_{CH}$, $^4J_{CH}$ = 2.0, 2.4 Hz, 2,5-position of C_5H_4 groups), 7.27 (2H, d, $^3J_{HH}$, = 7.6 Hz, 3,5-position of pyridine ring), 7.72 (1H, d, $^3J_{HH}$, = 7.6 Hz, 4-position of pyridine ring).
^{13}C{^{1}H} NMR (125.76 MHz, CD_3CN, 298 K): δ 32.2 (s, CH_2), 77.0 (s, 3,4-position of C_5H_4 groups), 88.0 (s, 2,5-position of C_5H_4 groups), 120.9 (s, 3,5-position of pyridine ring), 123,4 (s, 1-position of C_5H_4 groups), 139.3 (s, 4-position of pyridine ring), 153.1 (s, 2,6-position of pyridine ring).
^{15}N NMR (50.68 MHz, CD_3CN, 298 K, detected using ^{1}H-^{15}N HMBC): δ 319.5 (s).
^{19}F NMR (376.52 MHz, CD_3CN, 298 K): δ -151.76 (s, $^{10}BF_4$) and -151.82 (s, $^{11}BF_4$) in ca. 2:8 integration ratio due to natural abundance of ^{10}B and ^{11}B.
^{11}B NMR (128.38 MHz, CD_3CN, 298 K): δ -1.2 (s, BF_4).
Effective magnetic moment: μ_{eff} (Evans' method, CD_3CN, measured at 298 and 233 K) = 0 μ_B.
FTIR (Thin film, cm^{-1}): 1592 (w, C=C and C=N stretch), 1581 (w, C=C and C=N stretch), 1463 (w, sp^3 C-H bending), 1423 (w, sp^3 C-H bending), 950-1150 (very strong, orvelapping sp^2 C-H bending and B-F stretch), 866 (m, sp^2 C-H bending), 795 (m, sp^2 C-H bending).

Elemental analysis: Calcd: C:53.87, H:3.99, N:3.70. Found: C:54.22, H:3.61, N:3.79.
HRMS (ESI/Orbitrap, $[M-BF_4]^+$): Calcd for $C_{17}H_{15}NCo$: 292.0531. Found: 292.0528.

Preparation of [Mn(CpNCp)] (**4**)

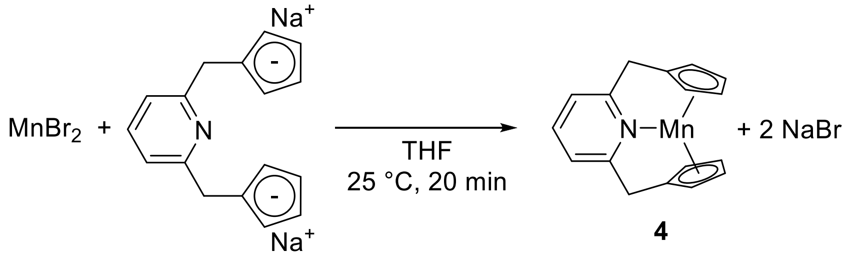

In a nitrogen glovebox, a 20 mL vial equipped with a Teflon coated stirring bar was charged with $MnBr_2$ (22.0 mg, 0.102 mmol) and 4 mL THF. The vial was sealed with a plastic cap, and the solution was stirred, and heated intermittently using a heat gun until all pink chunks of $MnBr_2$ dissolved. The resulting slightly cloudy colorless solution was cooled to 25 °C. To the solution was then added a solid of Na_2CpNCp (28.5 mg, 0.102 mmol) portionwise for ca. 2 min. The vial containing Na_2CpNCp was washed with 1 mL THF, and the THF solution was added to the $MnBr_2$ solution. A yellow solution and white precipitate were formed as soon as Na_2CpNCp was added. The solution was stirred for 20 min and concentrated to dryness. The product was extracted using ether and the yellow ether solution was filtered using plug of Celite, and the Celite was washed by ether. The concentration of combined solution gave yellow crystals of **4**. Yield: 22.6 mg, 77%. **4** is more sensitive to air and moisture. In an experiment, prolonged drying of **4** resulted in formation of pink solid which is insoluble in ether.
Supplementary Figs. 25-27 shows ^{1}H NMR, FTIR, and EPR spectrum of **4**.
^{1}H NMR (400.15 MHz, C_6D_6, 298 K): δ -102.1, -25.3 (overlapping with a signal at 3.2 ppm), 3.2 (overlapping with residual solvent signals and a signal at 31.4 ppm), 31.4 (overlapping with a signal at 3.2 ppm), 83.5 (overlapping with a signal at 31.4 ppm). Assignment of the signals was not possible due to extreme broadening and overlapping of the signals.
Effective magnetic moment: μ_{eff} (Evans' method, C_6D_6, 298.0 K) = 6.2 μ_B.
EPR (9.079 GHz, 11 mM toluene glass, 77 K): g_1, g_2, g_3 = 2.02, 1.95, 2.0. A_1, A_2, A_3 (^{55}Mn) = 220, 200, 210 MHz.
FTIR (Thin film, cm^{-1}): 1568 (m, C=C and C=N stretch), 1453 (m, sp^3 C-H bending), 1413 (m, sp^3 C-H bending), 1024 (m, sp^2 C-H bending), 787 (s, sp^2 C-H bending), 737 (s, sp^2 C-H bending).
Elemental analysis: Calcd for $C_{17}H_{15}NMn$: C:70.84, H:5.25, N:4.86. Found: C:70.76, H:5.20, N:4.78.
HRMS (ESI): Assignable signal was not detected due to higher air and moisture sensitivity of **4**.

Examination of possible coordination of pyridine to cobaltocene

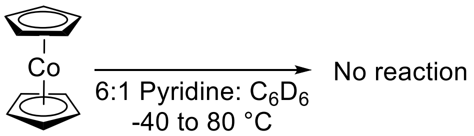

In a nitrogen glovebox, a 4 mL vial was charged with 10 μL $Me_3SiOSiMe_3$, 0.50 mL pyridine, and 0.10 mL C_6D_6. Cobaltocene (5.7 mg, 0.030 mmol) was completely dissolved in 0.50 mL of this solvent mixture, and the solution was transferred to a J. Young NMR tube. To the tube was then added a glass capillary containing the same solvent mixture. Effective magnetic moment (μ_{eff}) of the complex was calculated using ^{1}H NMR spectra recorded at 80, 25, and -40 °C, and was 1.8, 1.9, and 1.9 μ_B, respectively. The slight change of μ_{eff} is likely due to change of solvent density upon temperature changes, which causes slight change of solution concentration. ^{1}H NMR signals with chemical shift similar to **1** were not detected. The NMR sample was transferred to an EPR tube in a nitrogen glove box, and EPR spectra was recorded at 4.2 K. Signals expected for the formation of pyridine-coordinated, S = 3/2 cobaltocene species, [$CoCp_2$(pyridine)], were not detected.

2. NMR and IR spectra and VSM data

A

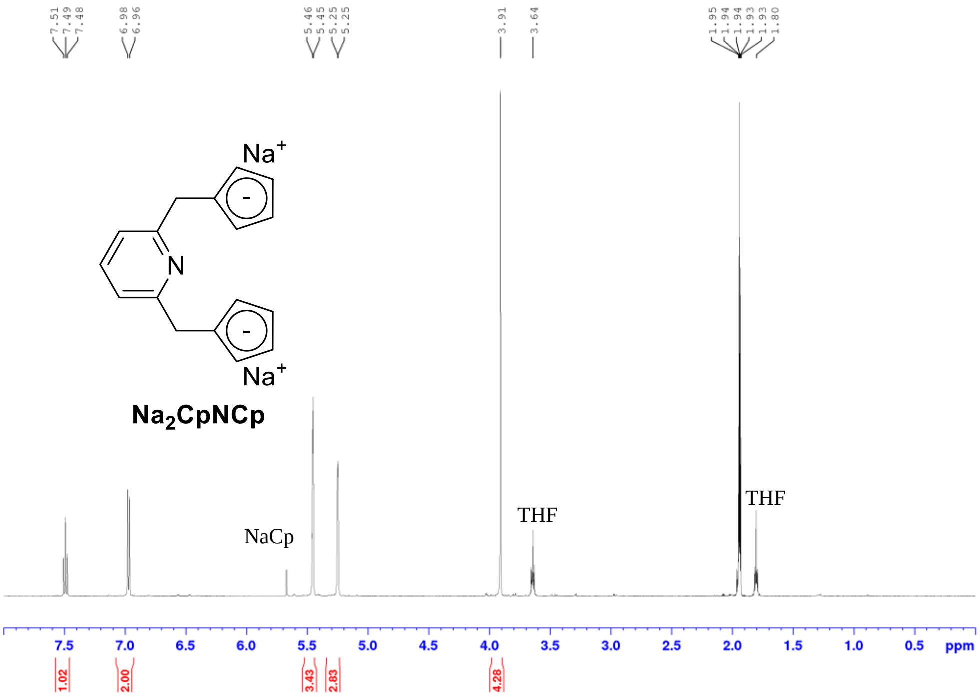

B

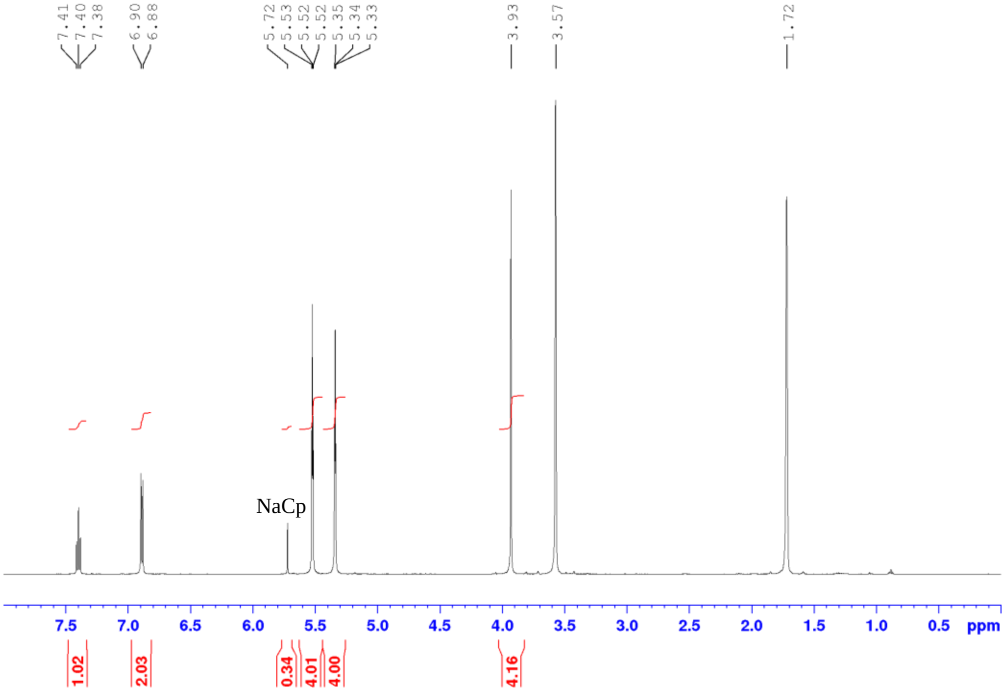

Supplementary Fig. 1.

^{1}H NMR spectrum (500.13 MHz, 298 K) of **Na_2CpNCp**. (**A**) In CD_3CN. Partially deuterated due to deuteration of Cp protons by CD_3CN. (**B**) In THF-d_8.

A

B

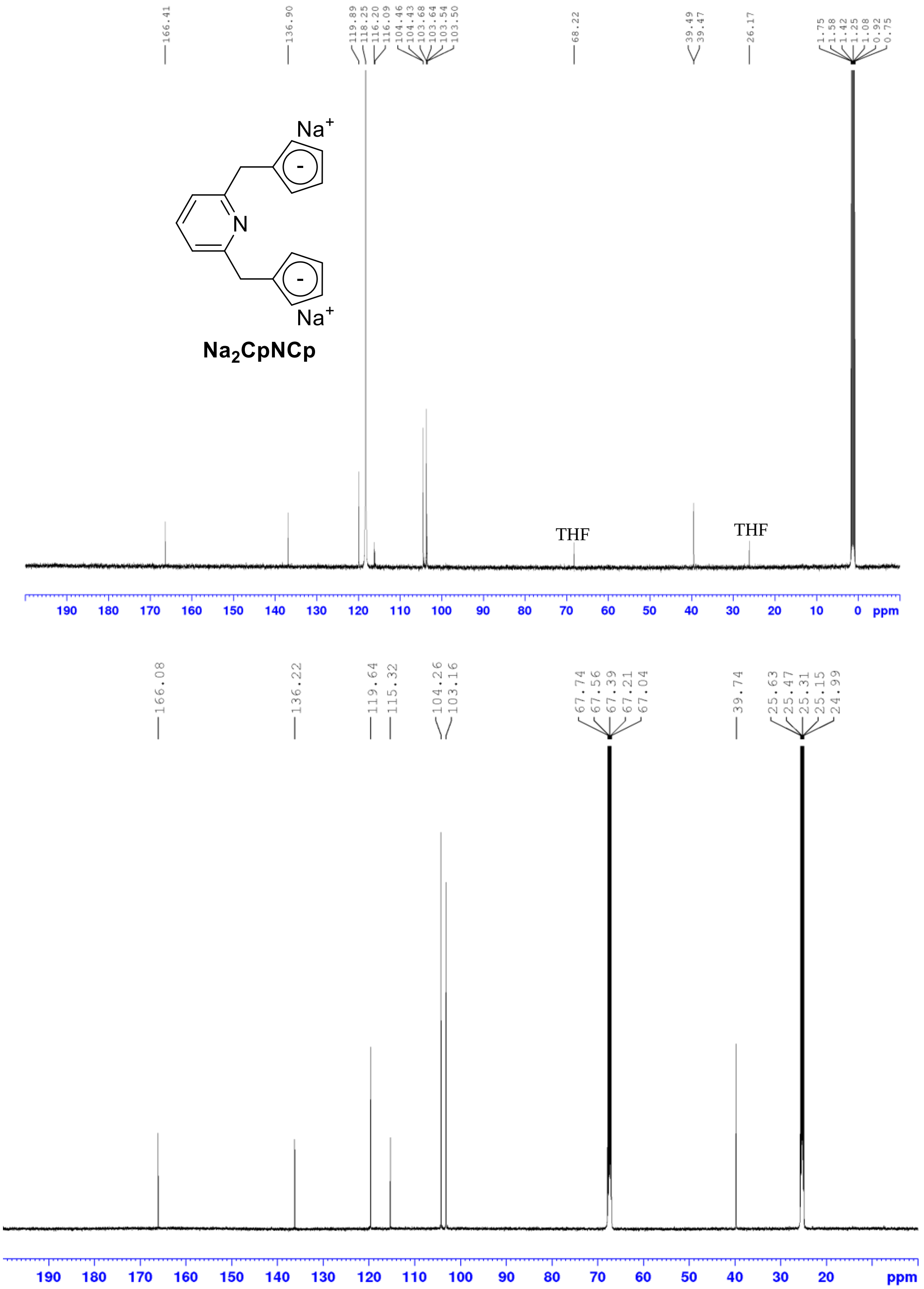

Supplementary Fig. 2.

$^{13}C\{^{1}H\}$ NMR spectrum (125.76 MHz, 298 K) of **Na_2CpNCp**. (**A**) In CD_3CN. Partially deuterated due to deuteration of Cp protons by CD_3CN. (**B**) In THF-d_8.

A

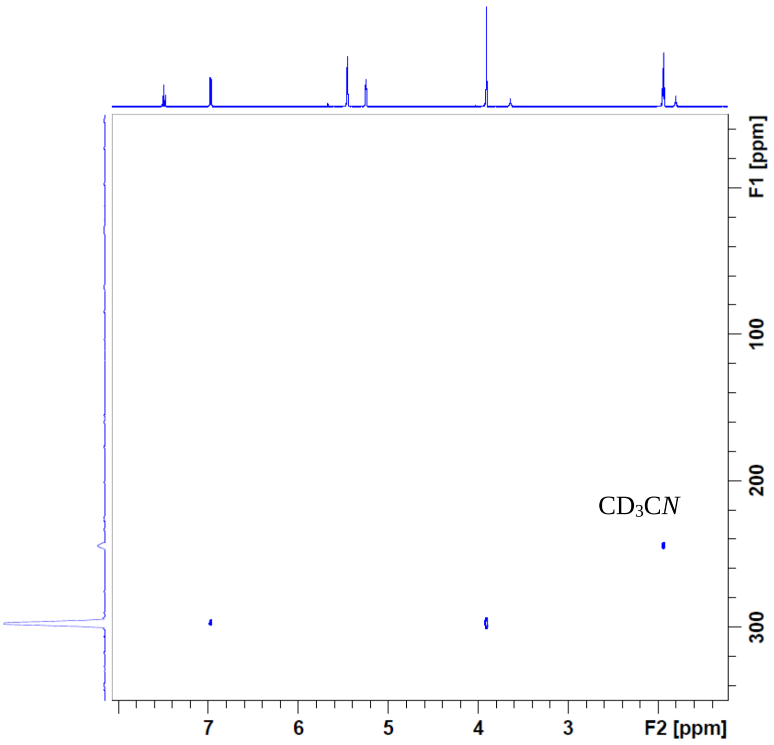

B

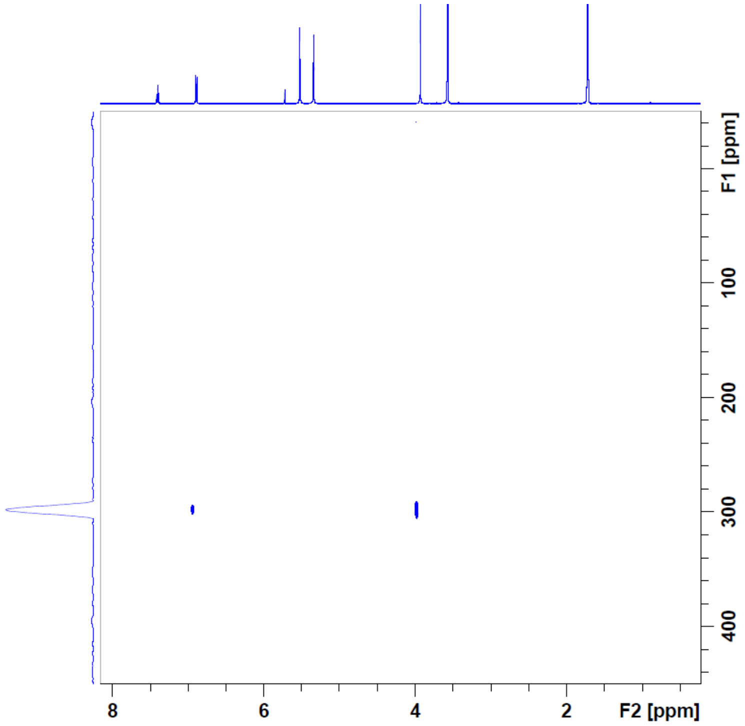

Supplementary Fig. 3.

^{1}H-^{15}N HMBC NMR spectrum (CD_3CN, 298 K) of **Na_2CpNCp**. (**A**) In CD_3CN. Partially deuterated due to deuteration of Cp protons by CD_3CN. (**B**) In THF-d_8.

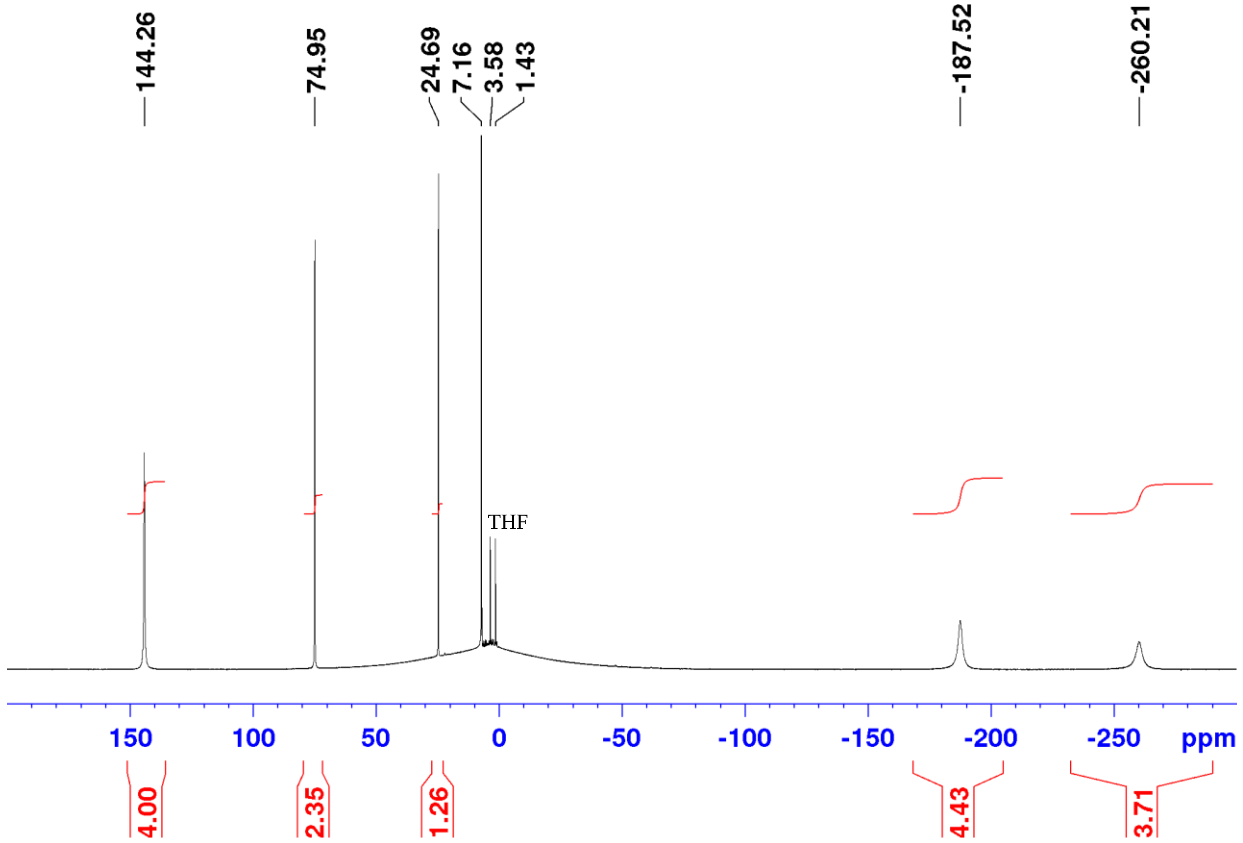

Supplementary Fig. 4.
^{1}H NMR spectrum (400.15 MHz, C_6D_6, 298 K) of **1**.

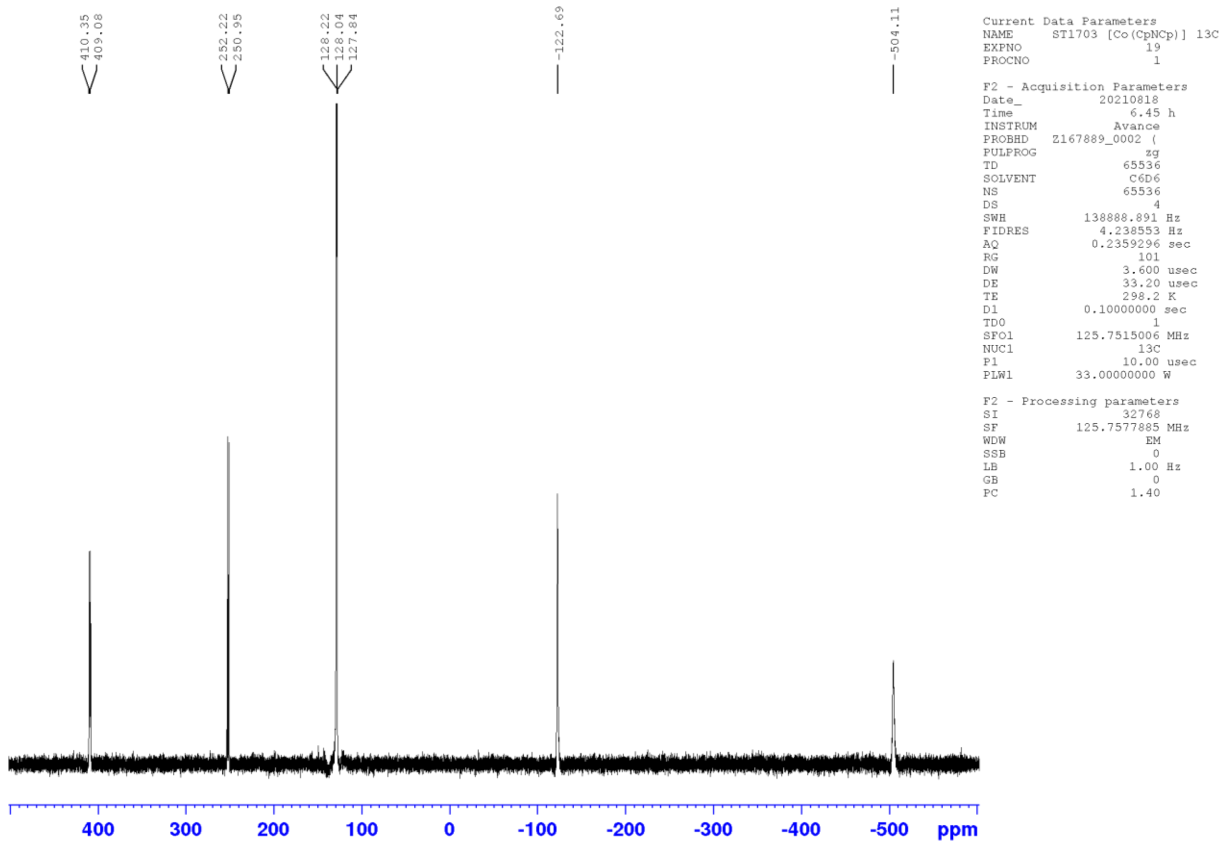

Supplementary Fig. 5.

Proton coupled ^{13}C NMR spectrum (125.76 MHz, C_6D_6, 298 K) of **1**.

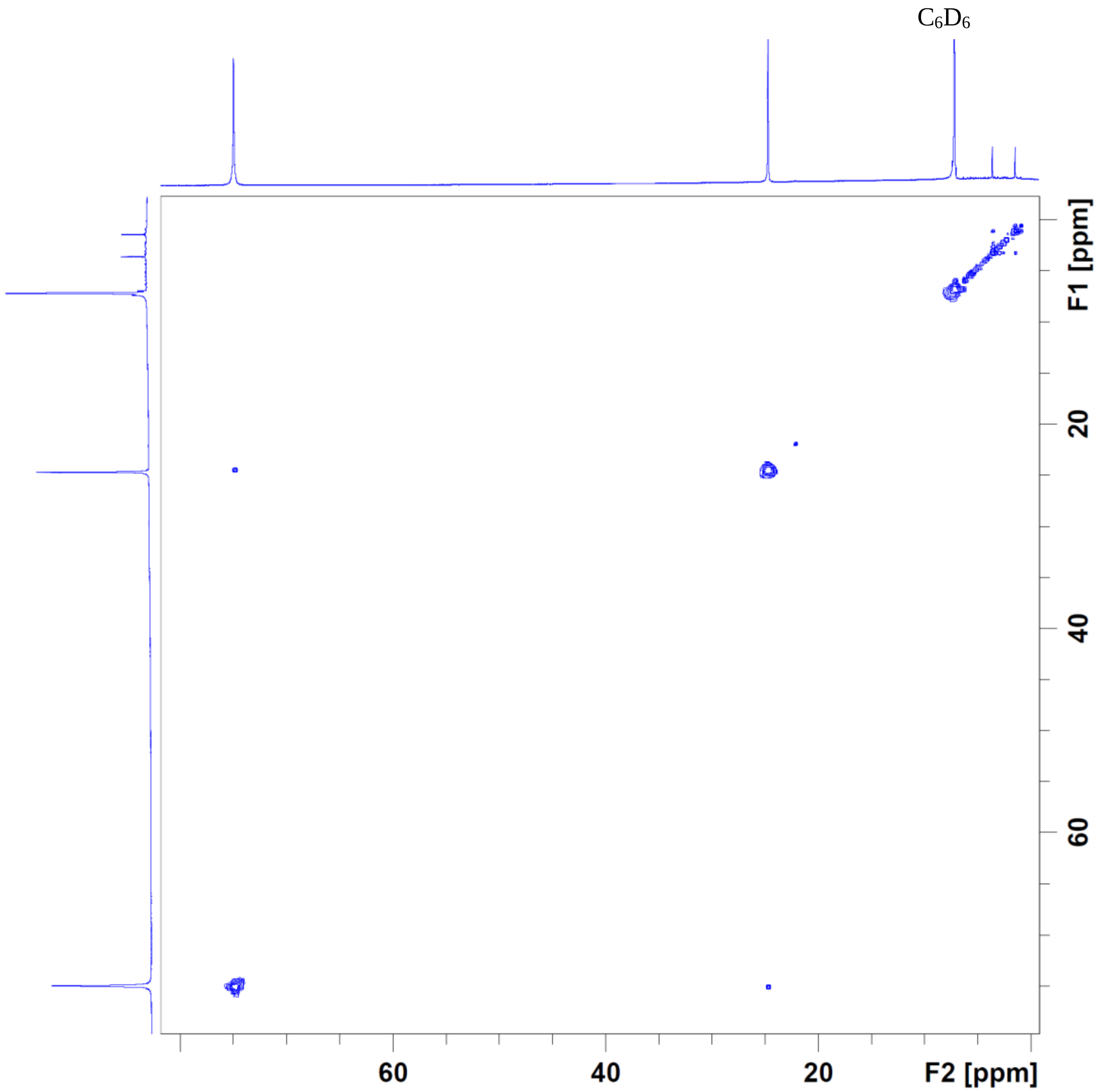

Supplementary Fig. 6.
^{1}H-^{1}H COSY NMR spectrum (400.13 MHz, C_6D_6, 298 K) of **1**.

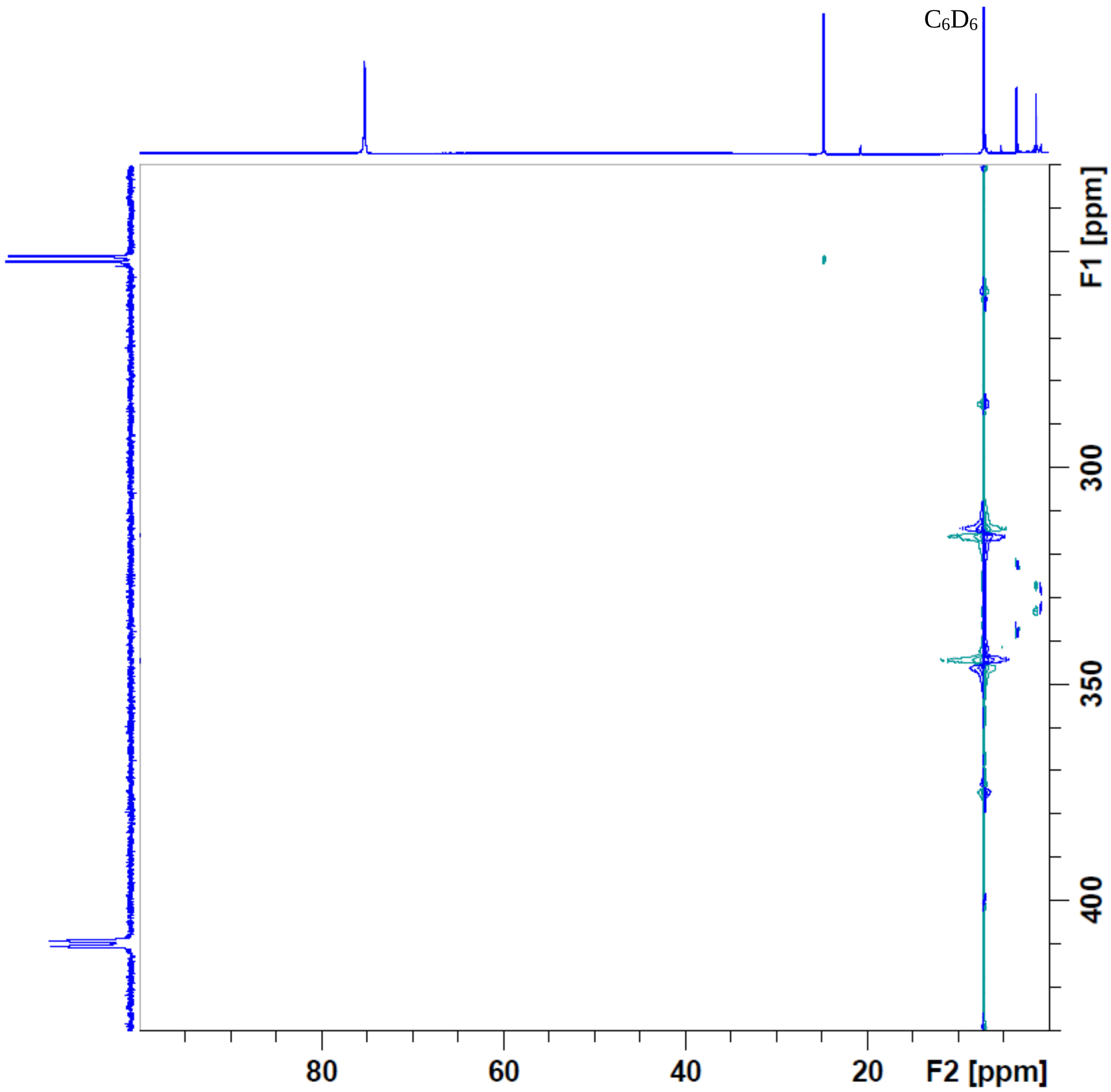

Supplementary Fig. 7.
^{1}H-^{13}C HSQC NMR spectrum (C_6D_6, 298 K) of **1**.

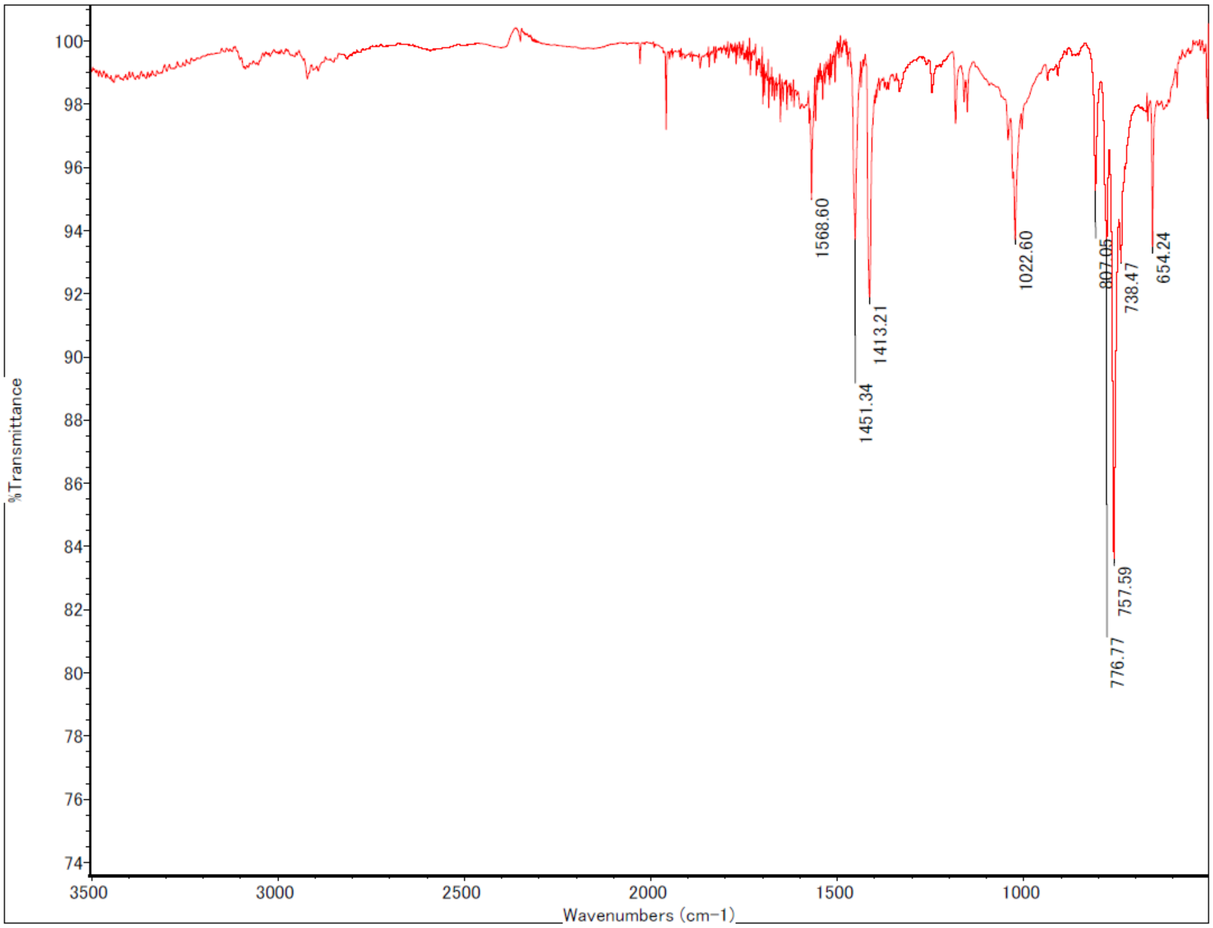

Supplementary Fig. 8.
FTIR spectrum (thin film) of **1**.

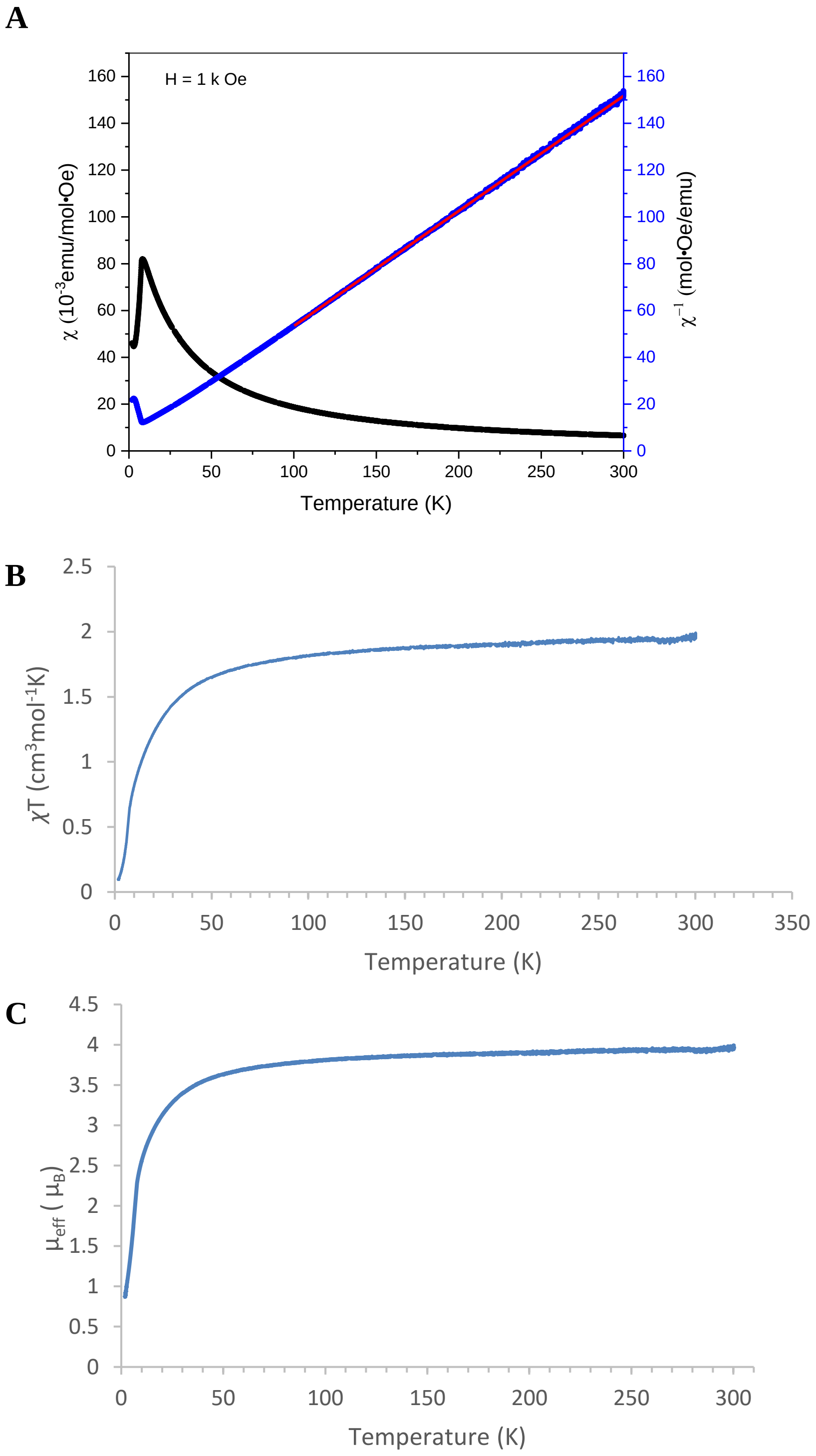

Supplementary Fig. 9.

VSM measurement of **1** at 2 -300 K under 1000 Oe. (**A**) Molar magnetic susceptibility (χ) vs. Temperature (T) (Black) and χ^{-1} vs. T (Blue) plots. The red solid line indicates the Curie-Weiss fitting. (**B**) χT vs. T plot. (**C**) Effective magnetic moment (μ_{eff}) vs. T plot.

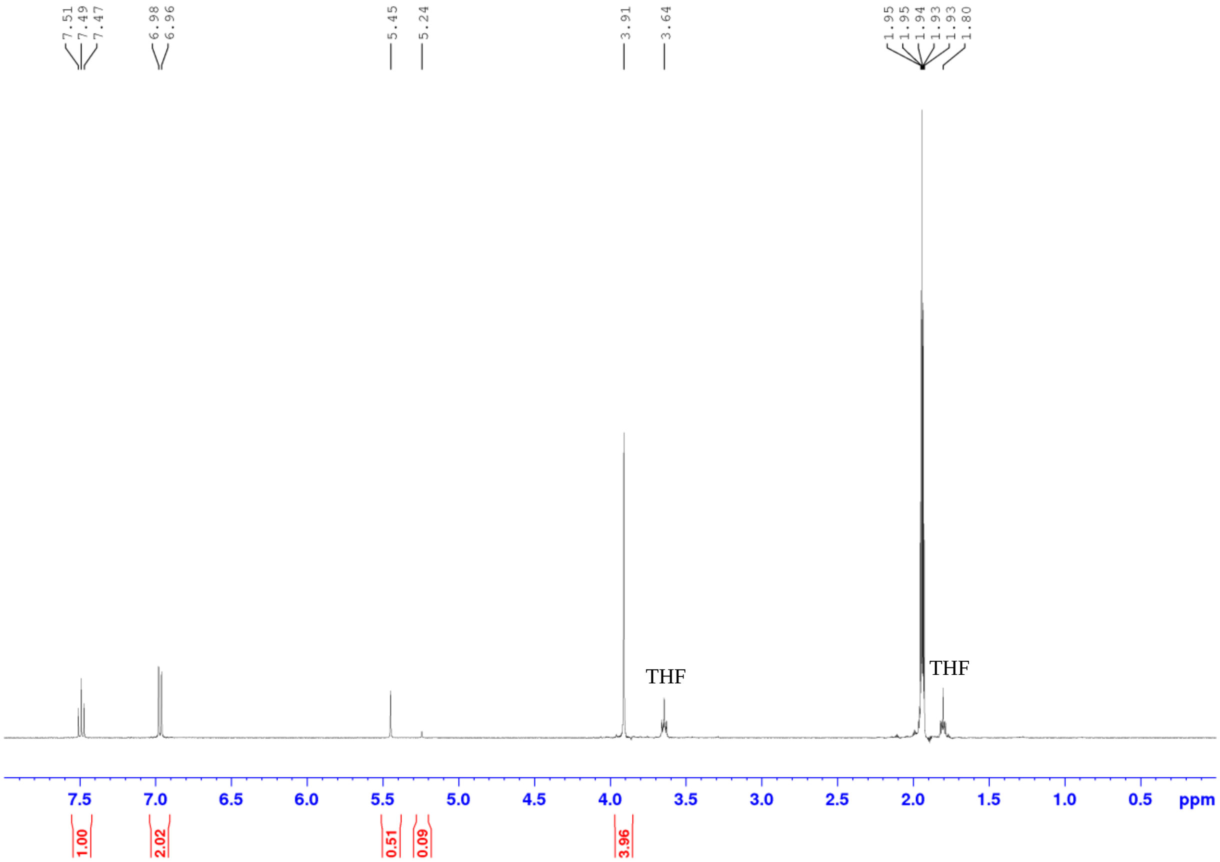

Supplementary Fig. 10.

^{1}H NMR spectrum (400.15 MHz, CD_3CN, 298 K) of **Na_2CpNCp-*d_8***.

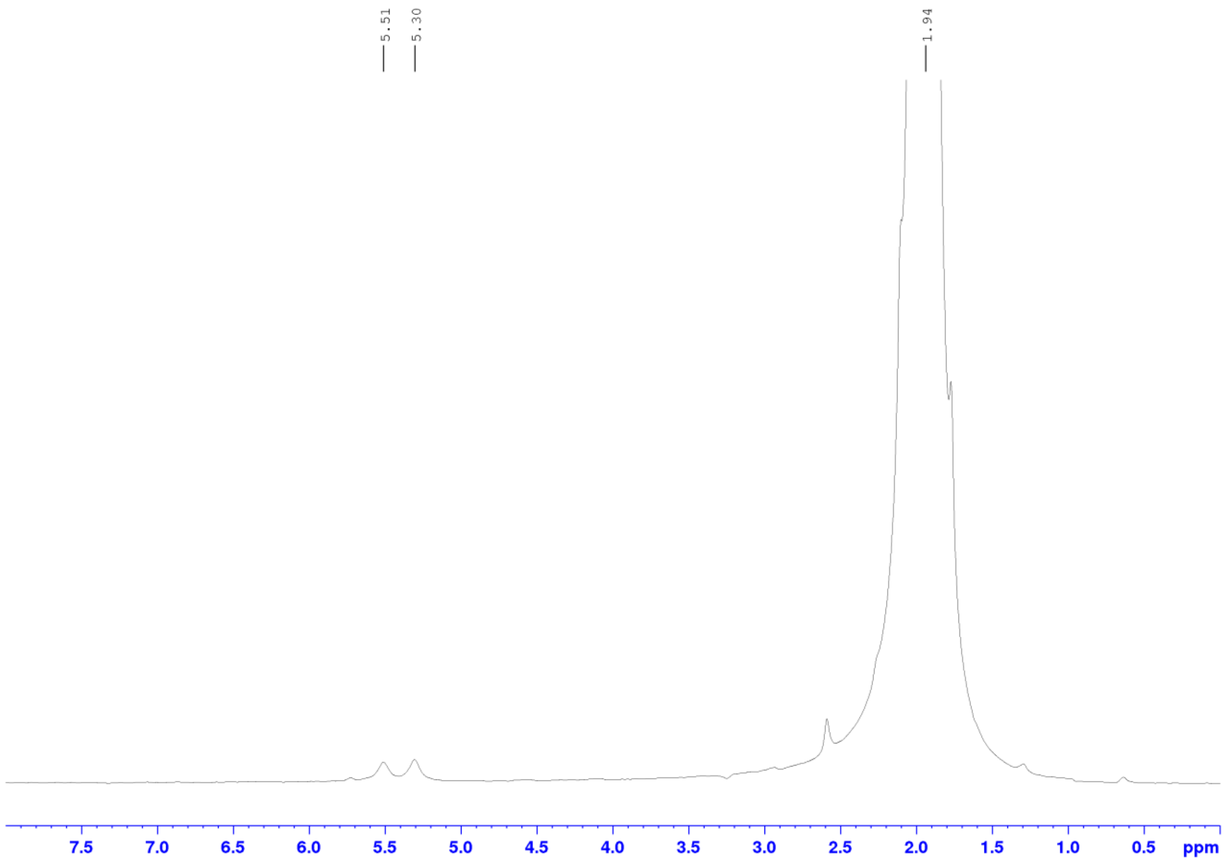

Supplementary Fig. 11.
^{2}H NMR spectrum (61.42 MHz, CD_3CN, 298 K) of **Na_2CpNCp-*d_8***.

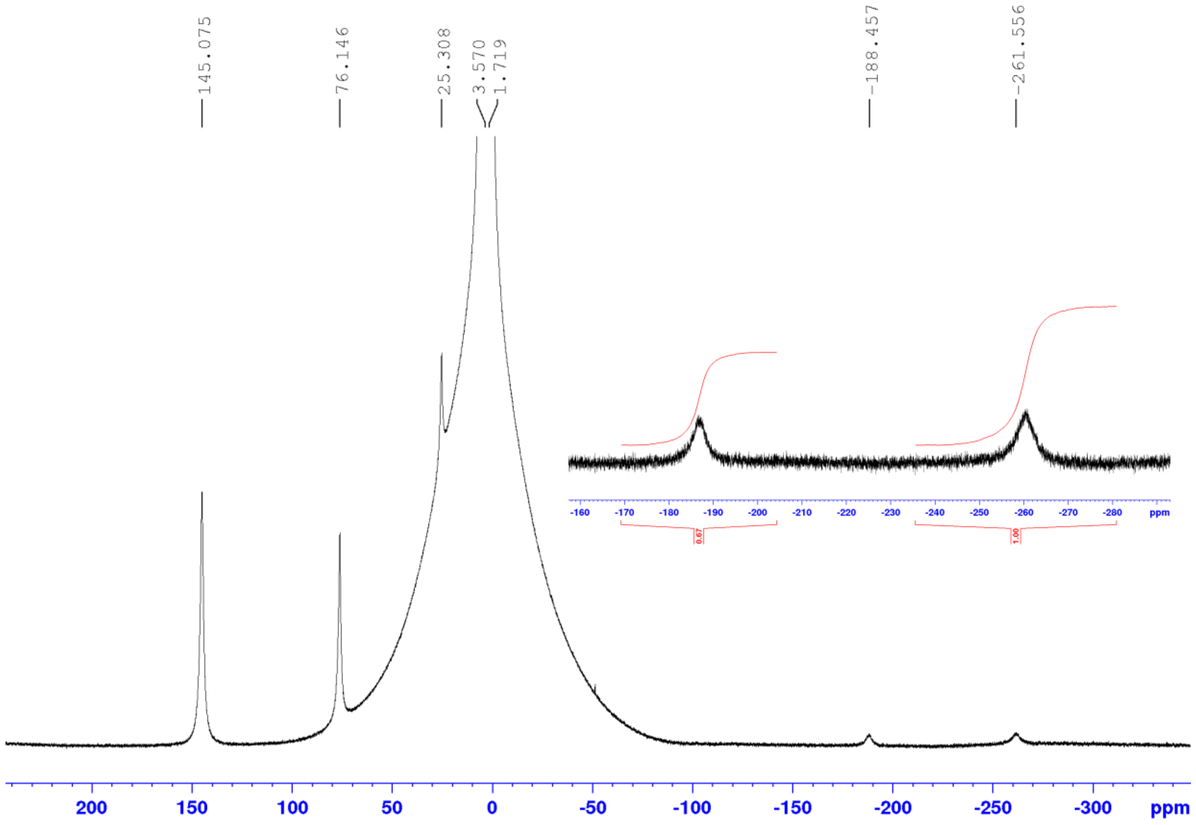

Supplementary Fig. 12.

^{1}H NMR spectra (400.15 MHz, THF-d_8, 298 K) of **1-d_8** (with 95 and 90% deuteration of 2,5-position and 3,4-position of cyclopentadienyl groups, respectively) showing change of signal intensity at -260.5 and -187.7 ppm. Inset: expansion of -160 to -290 ppm region showing ratio of the two signals from cyclopentadienyl groups.

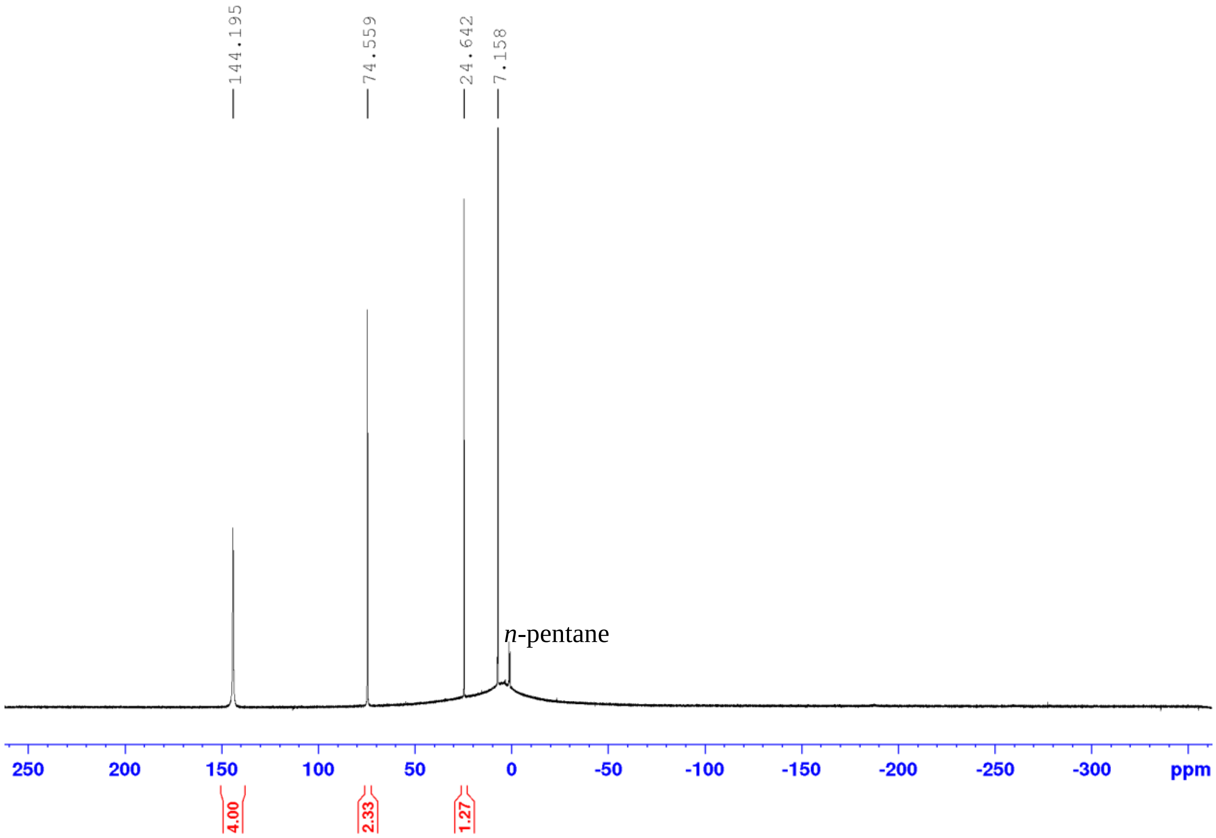

Supplementary Fig. 13.

^{1}H NMR spectra (400.15 MHz, C_6D_6, 298 K) of **1-*d₈*** (with 98% deuteration of 2,5-position and 3,4-position of cyclopentadienyl groups).

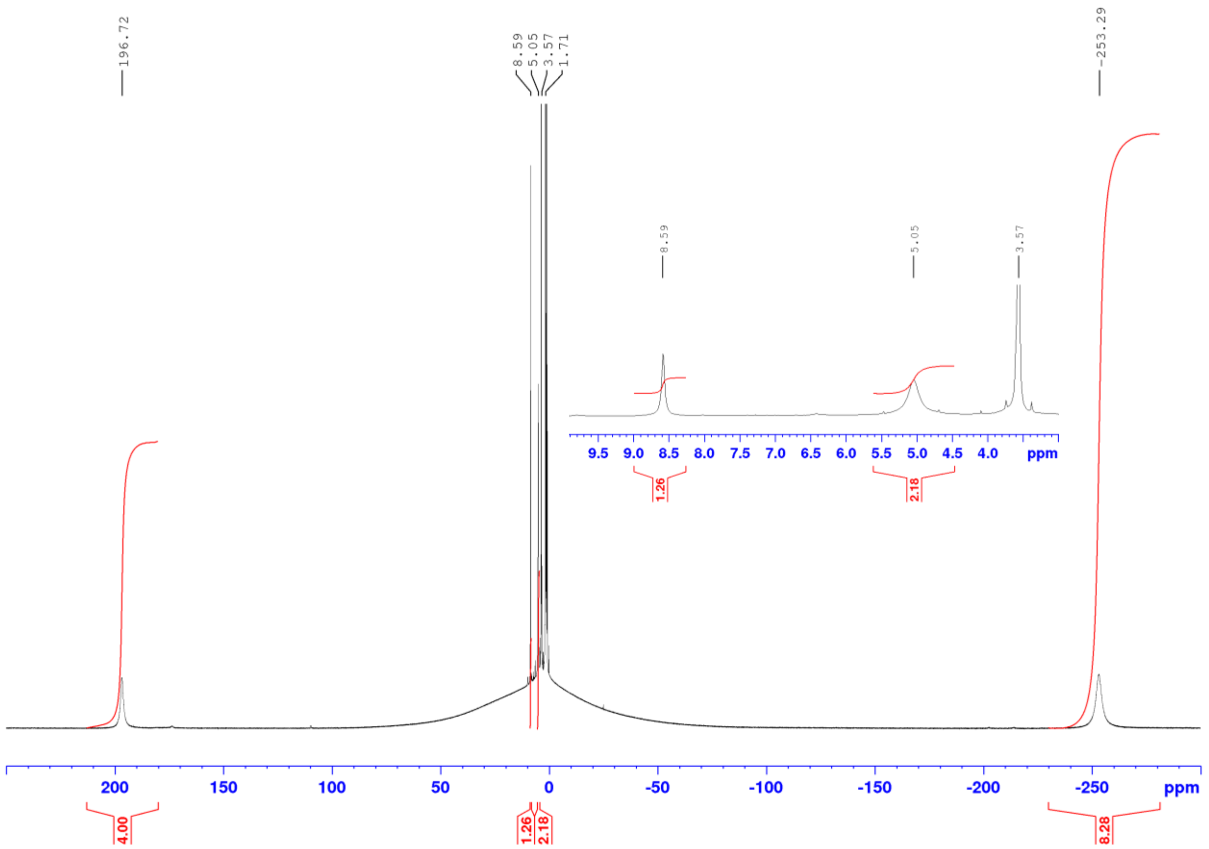

Supplementary Fig. 14.

^{1}H NMR spectrum (400.15 MHz, THF-d_8, 298 K) of **2**.

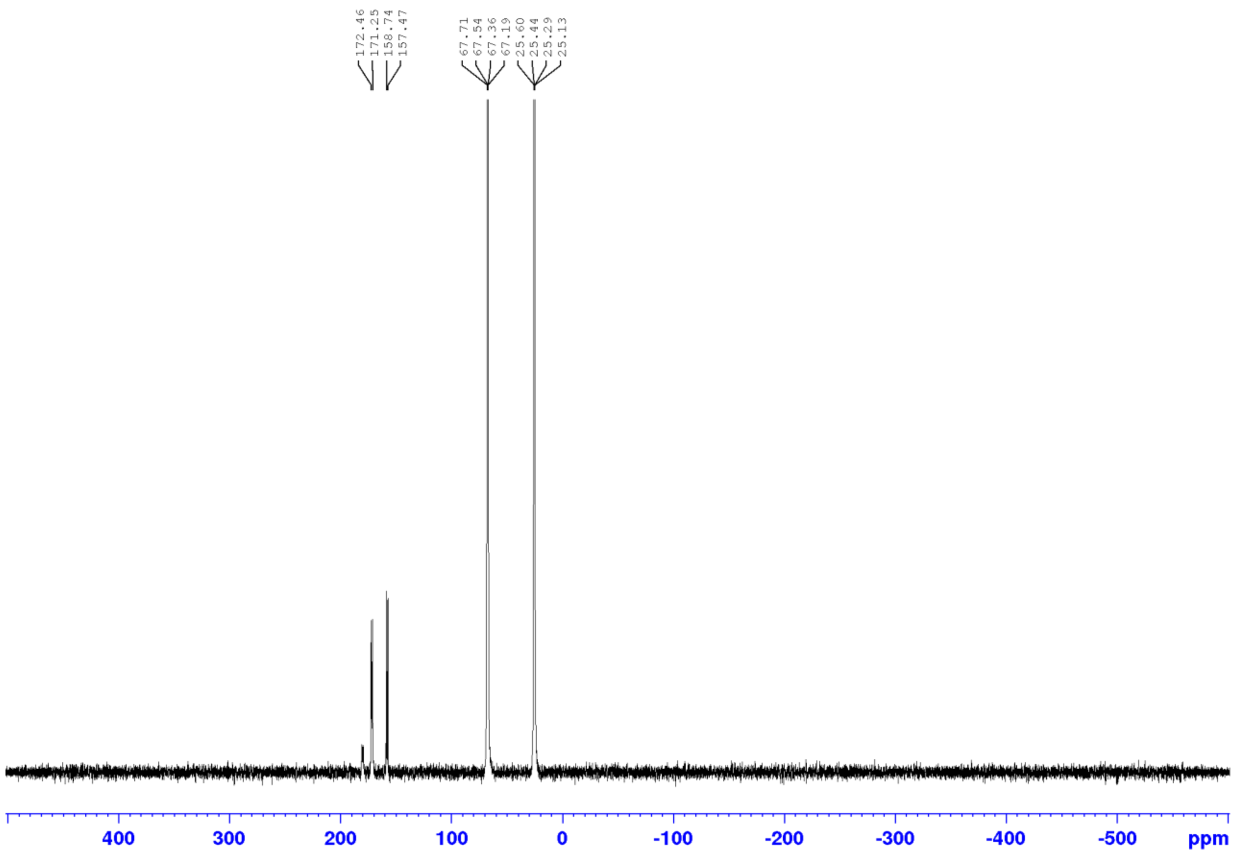

Supplementary Fig. 15.
Proton coupled ^{13}C NMR spectrum (125.76 MHz, THF-d_8, 298 K) of **2**.

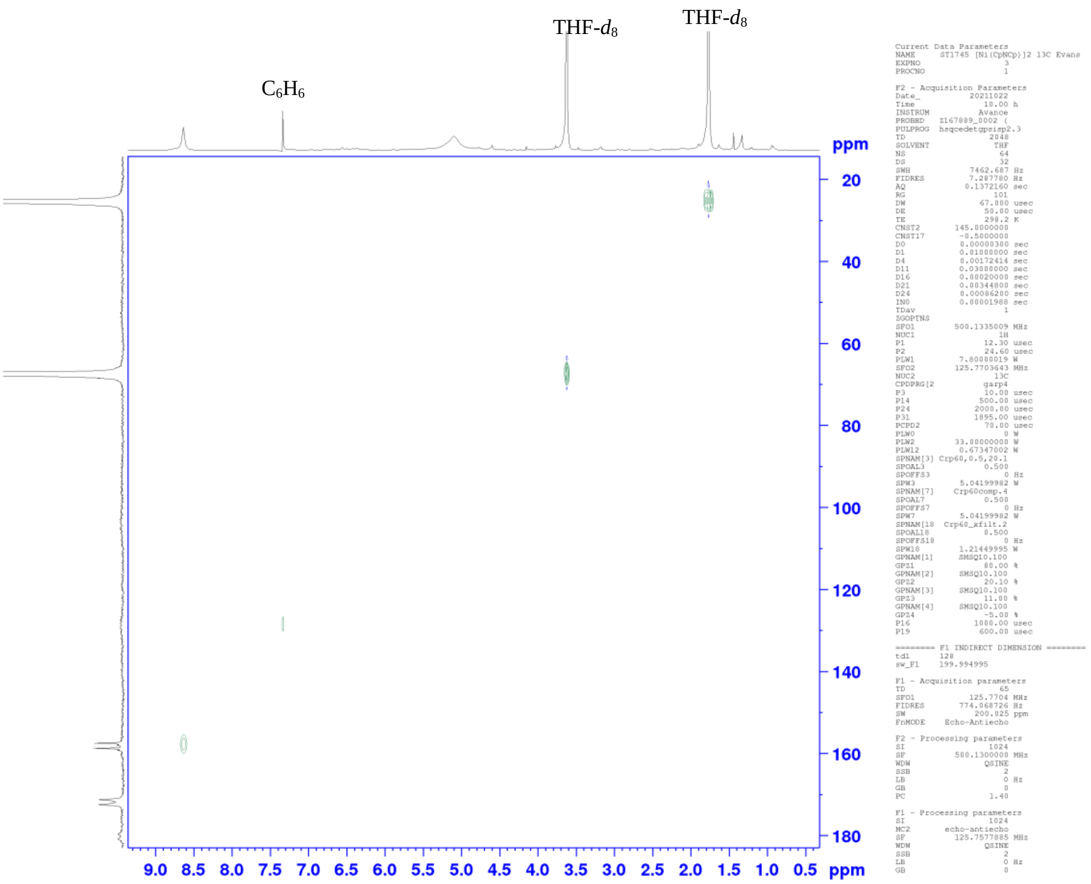

Supplementary Fig. 16.

^1H-^{13}C HSQC NMR spectrum (THF-d_8, 298 K) of **2**.

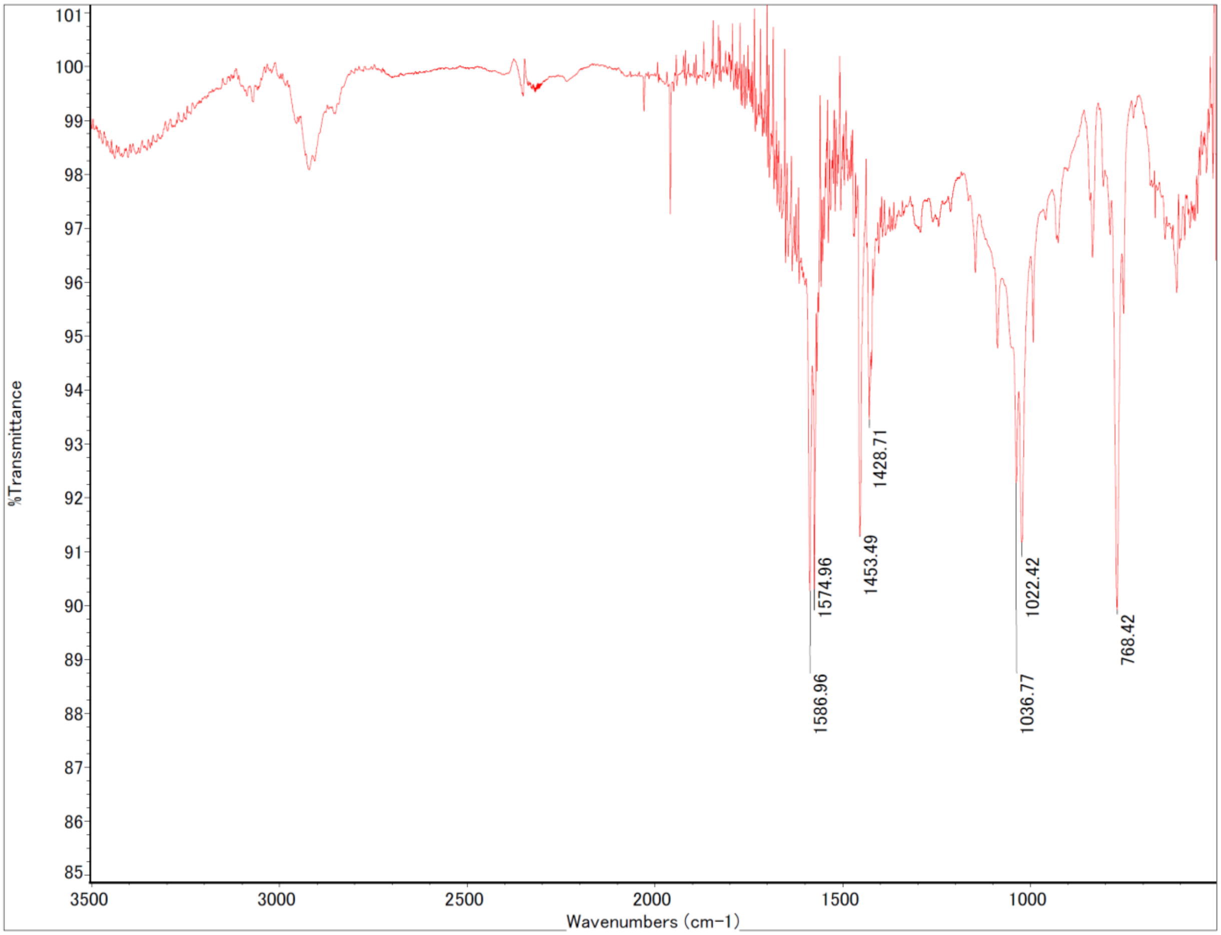

Supplementary Fig. 17.
FTIR spectrum (thin film) of **2**.

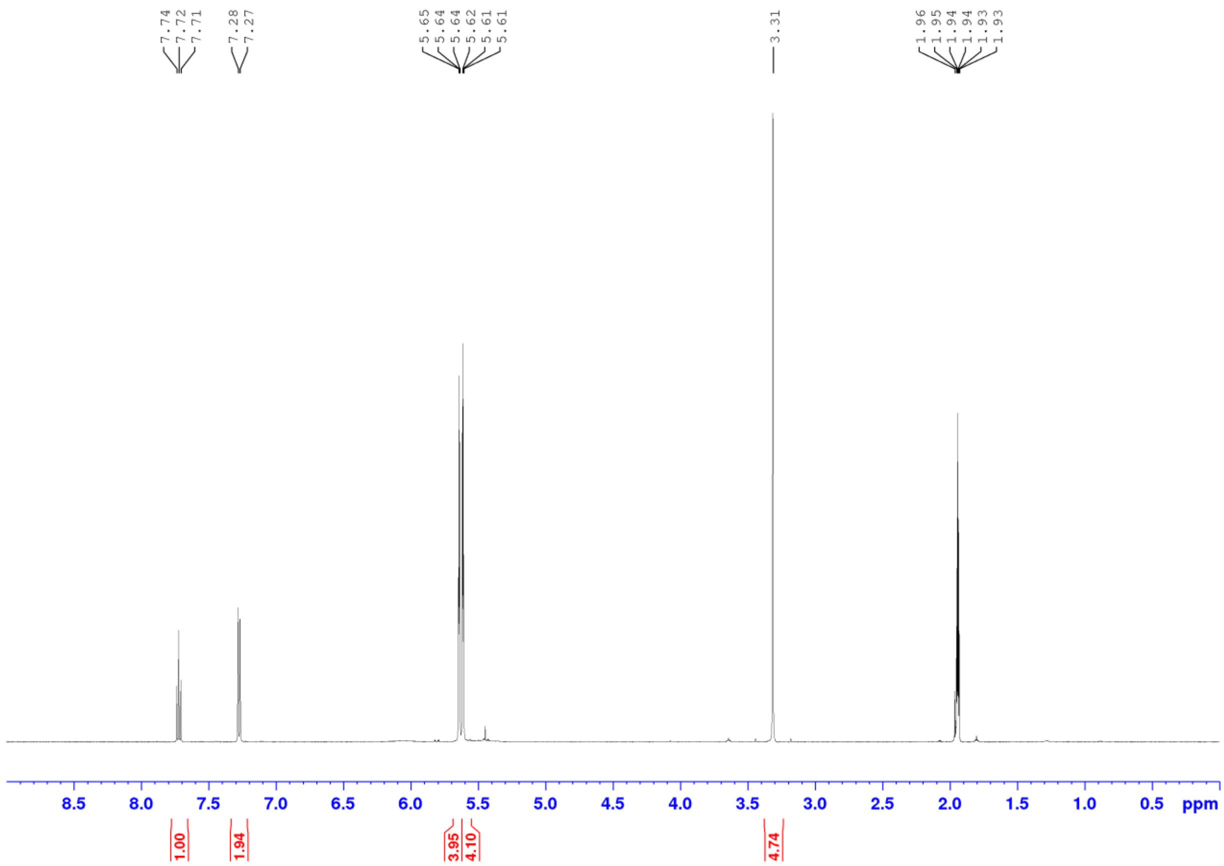

Supplementary Fig. 18.

^{1}H NMR spectrum (500.13 MHz, CD_3CN, 298 K) of **3**.

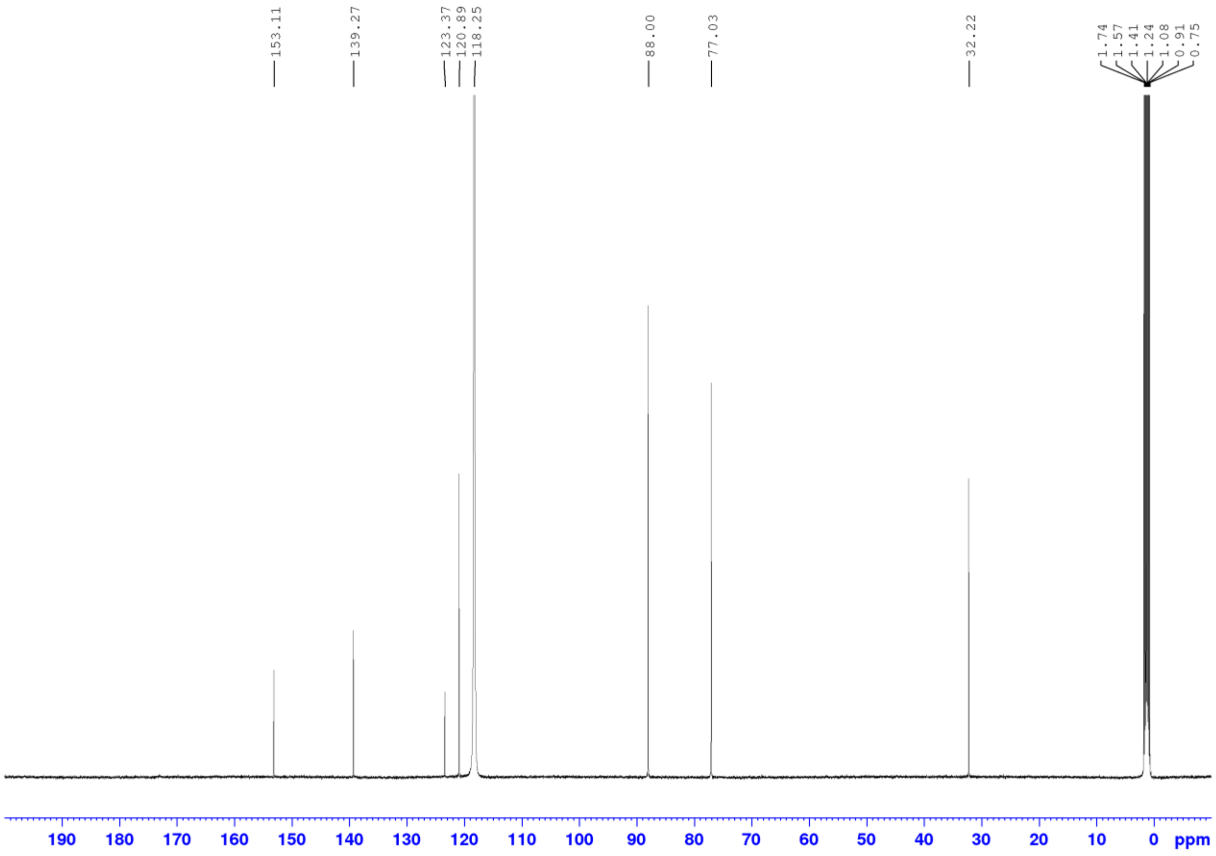

Supplementary Fig. 19.
$^{13}C\{^1H\}$ NMR spectrum (125.76 MHz, CD_3CN, 298 K) of **3**.

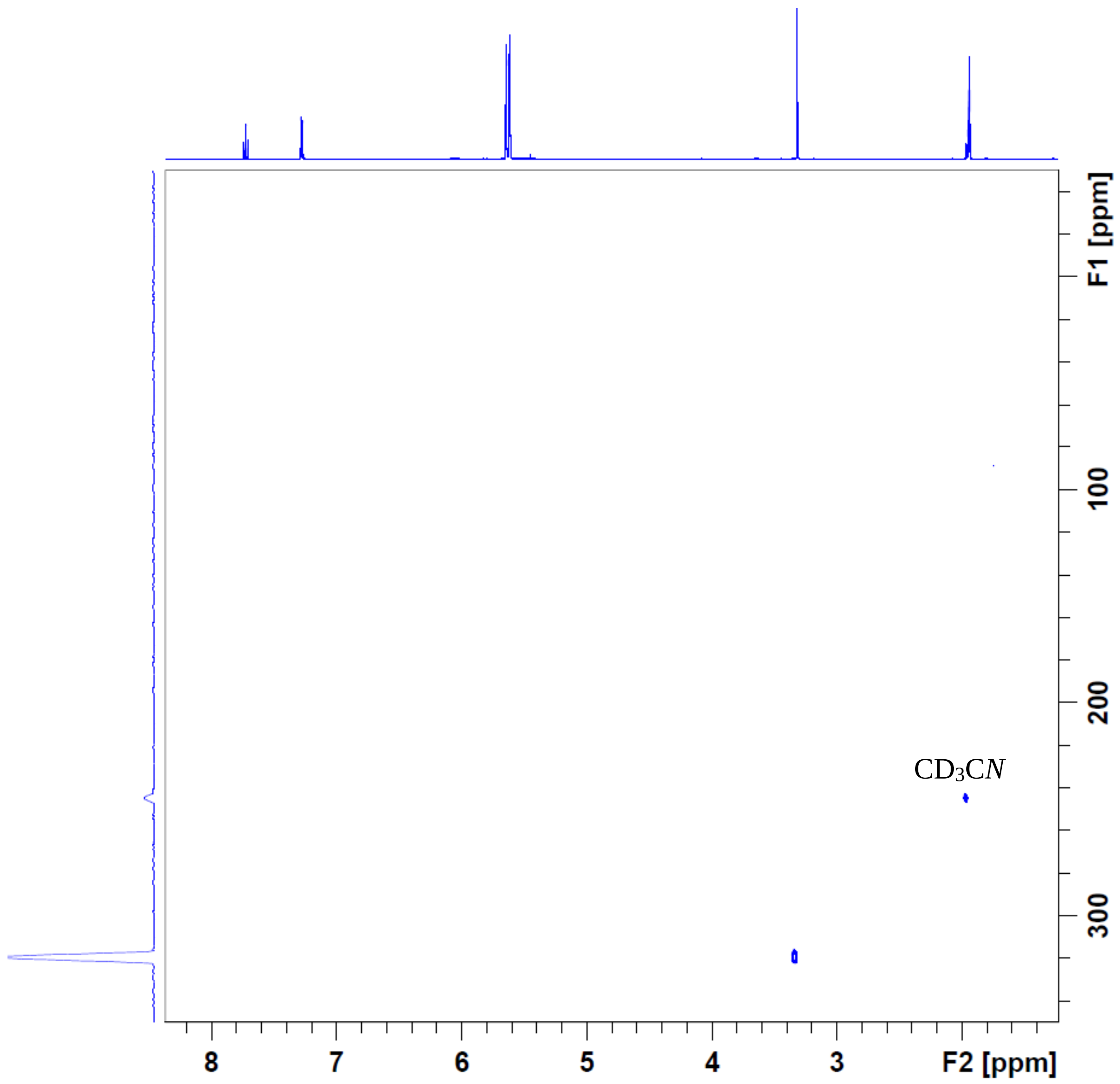

Supplementary Fig. 20.
^{1}H-^{15}N HMBC NMR spectrum (CD_3CN, 298 K) of **3**.

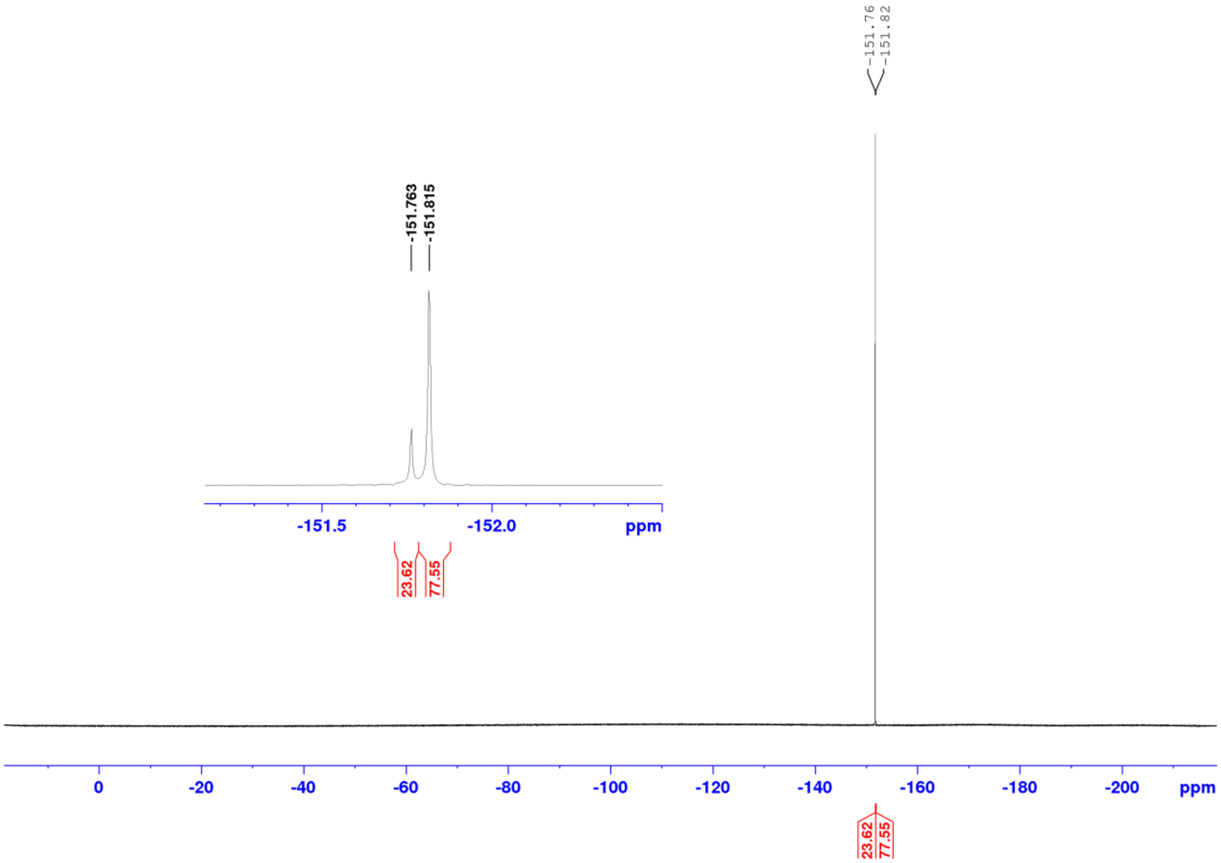

Supplementary Fig. 21.
^{19}F NMR spectrum (376.52 MHz, CD_3CN, 298 K) of **3**.

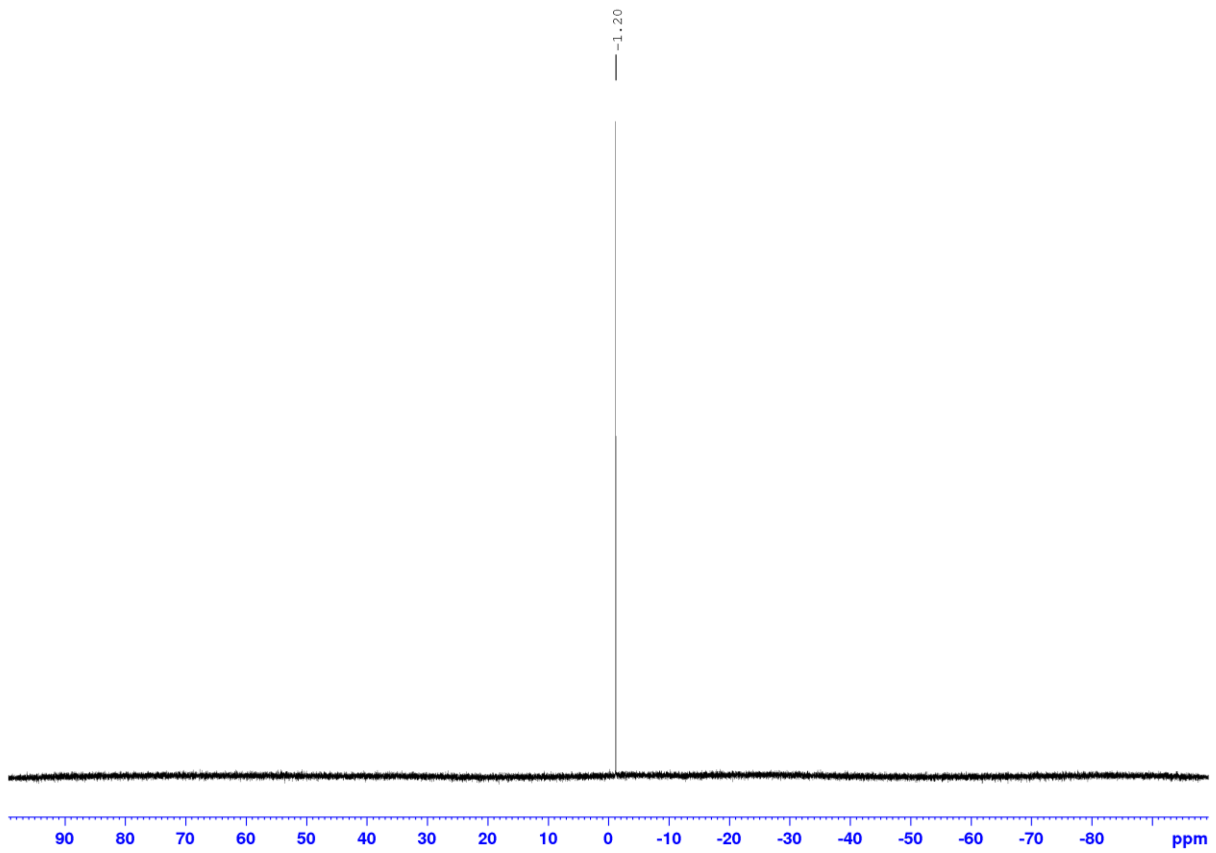

Supplementary Fig. 22.
^{11}B NMR spectrum (128.38 MHz, CD_3CN, 298 K) of **3**.

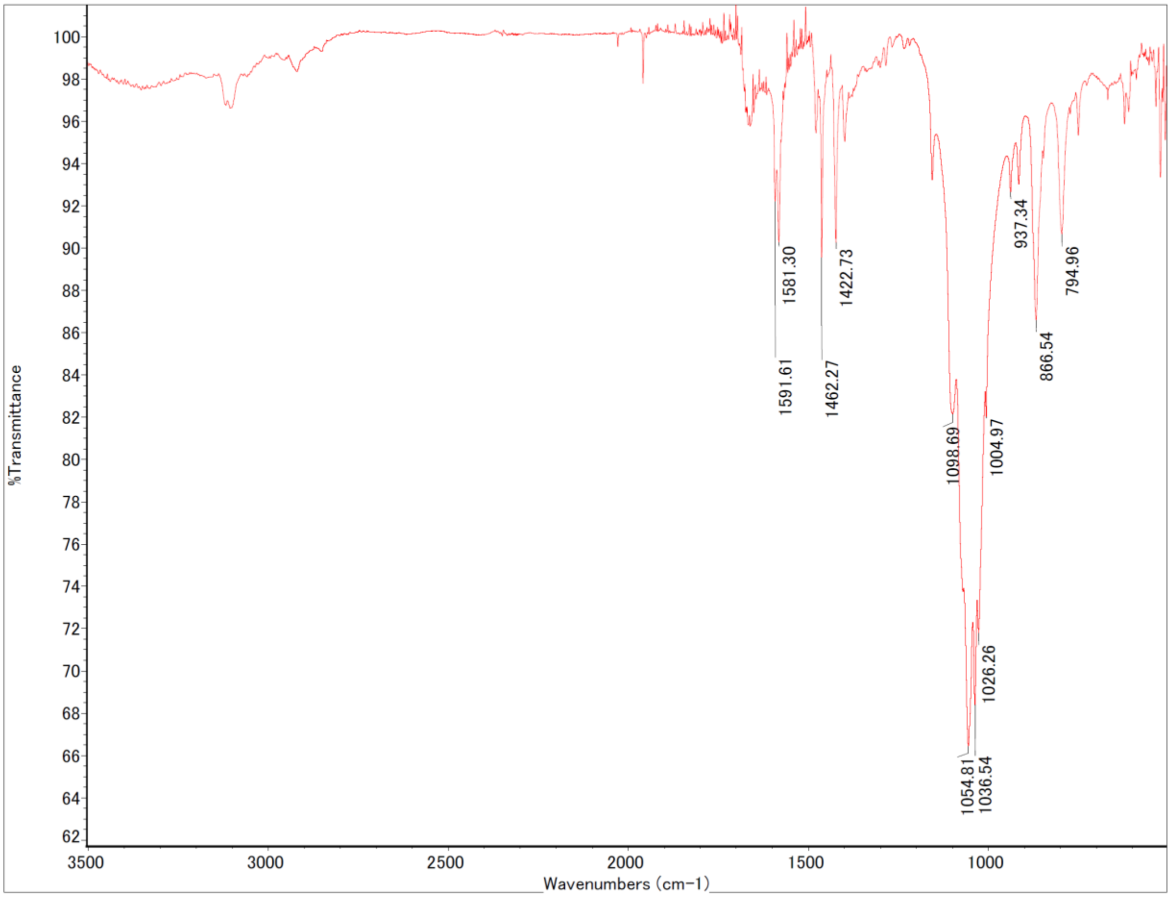

Supplementary Fig. 23.
FTIR spectrum (thin film) of **3**.

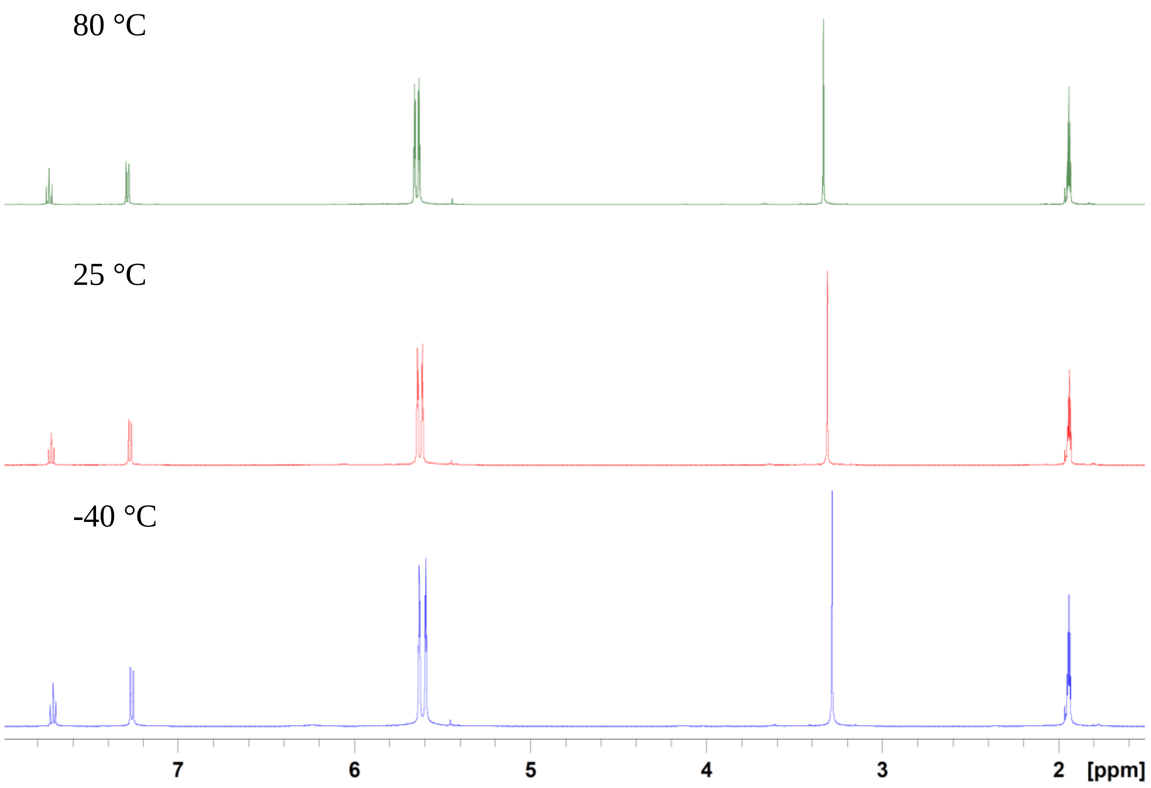

Supplementary Fig. 24.
Variable temperature ^{1}H NMR spectra (500.13 MHz, CD_3CN) of **3**.

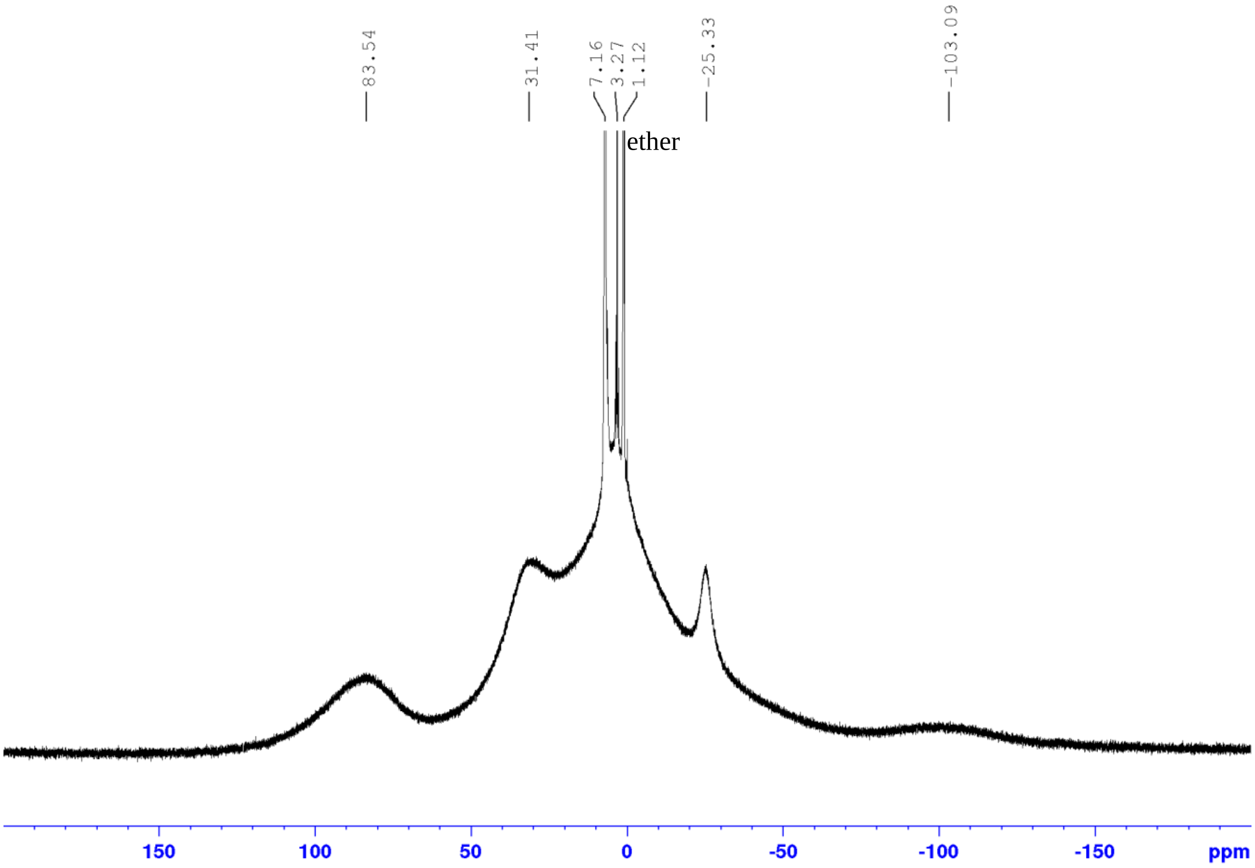

Supplementary Fig. 25.
^{1}H NMR spectrum (400.15 MHz, C_6D_6, 298 K) of **4**.

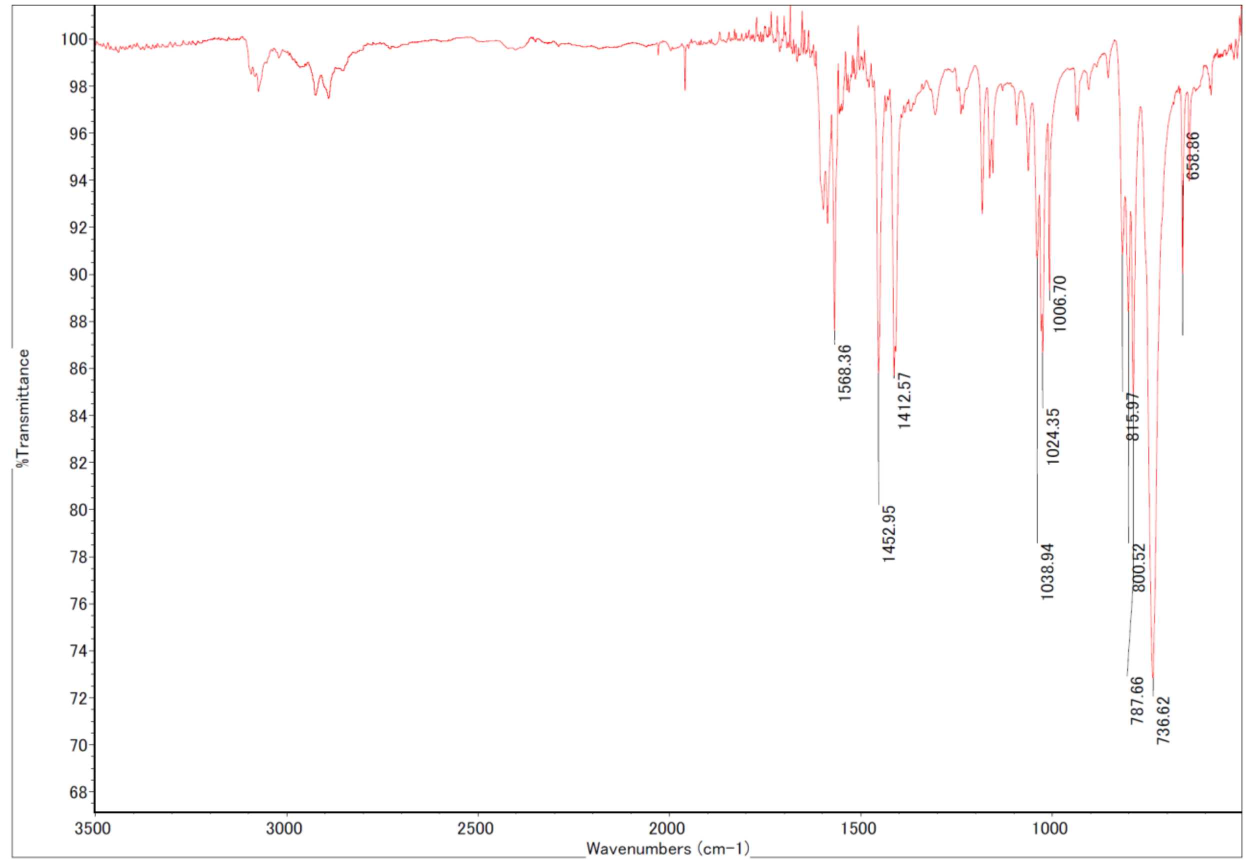

Supplementary Fig. 26.
FTIR spectrum (thin film) of **4**.

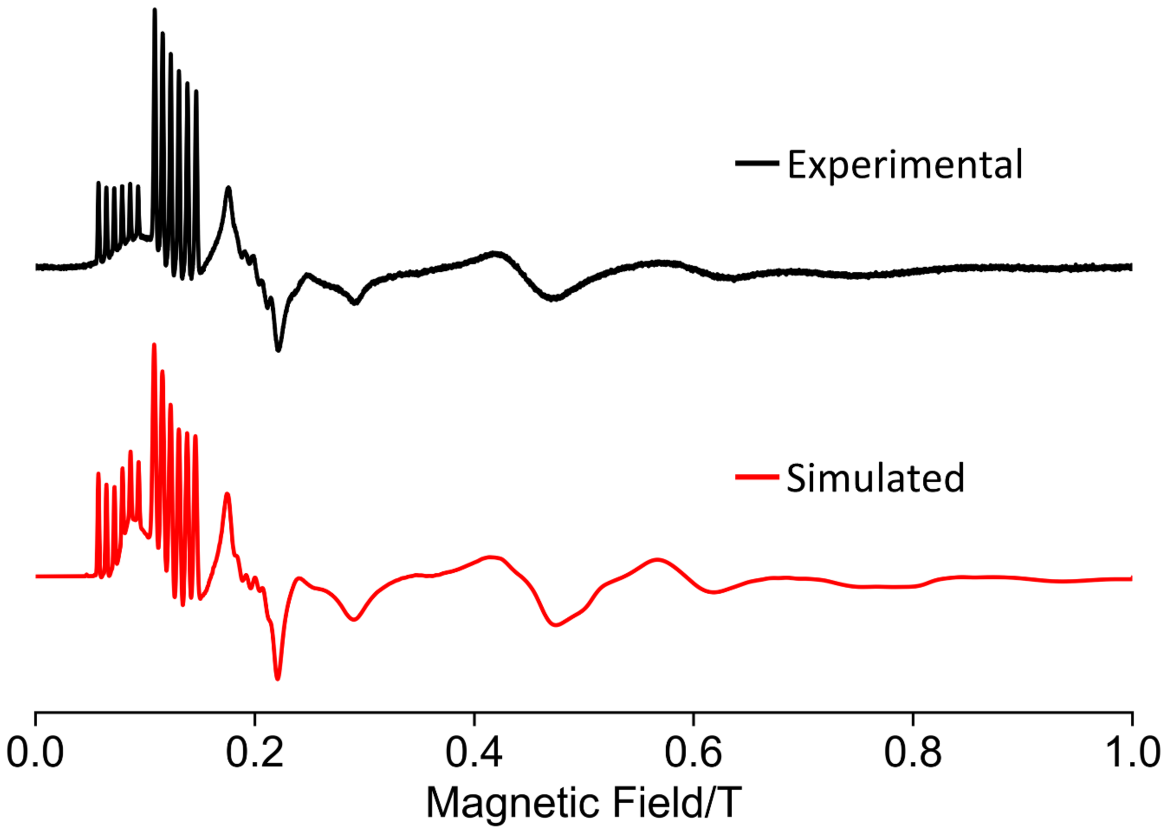

Supplementary Fig. 27.

Experimental (77 K, toluene glass) and simulated EPR spectra of **4** using S = 5/2 and D and E values. Simulation parameters: S = 5/2, g= [2.02, 1.95, 2.0], $A(^{55}Mn)$ = [220, 200, 210] MHz, D = 0.22 cm^{-1}, E = 0.039 cm^{-1}, linewidth = 1 mT, *D*Strain = [450, 80] MHz

3. EPR studies

EPR samples were prepared in a nitrogen glovebox using 5 mm outer diameter quartz EPR tubes sealed with rubber septums and parafilm. Continuous wave (cw) X-band EPR spectra of **1** were recorded using a JEOL JES-X330 X-band EPR spectrometer equipped with a liquid helium cooling system. The instrumental parameters employed were as follows: power: 1 mW; time constant: 100 ms; experimental frequency: 9.079 GHz; modulation amplitude: 40 and modulation width: ±0.06 mT.

Pulse EPR measurements were performed using a Bruker ESP380E spectrometer equipped with an Oxford liquid He-flow temperature controller. The spectrometer is customized with a TTL/ECL conversion unit and controlled by the SpecMan4EPR software [2]. Cw- and pulse-EPR spectra were simulated by using the EasySpin software [3,4].

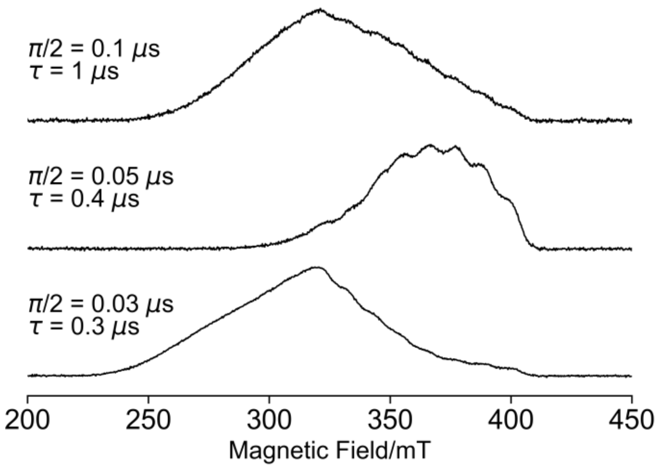

Supplementary Fig. 28.

Hahn-echo-detected EPR spectra of **1** observed at 4 K. Hahn-echo signal intensities were sensitive to the applied pulse length and pulse-duration time. The middle EPR spectrum dominantly includes the Z components of the spin system in the angular selective manner. Below 300mT, the intensity gradually decreases due to the fast phase-memory time T_2, indicating the difficulties of the detection pulse-EPR signals in the lower magnetic field range.

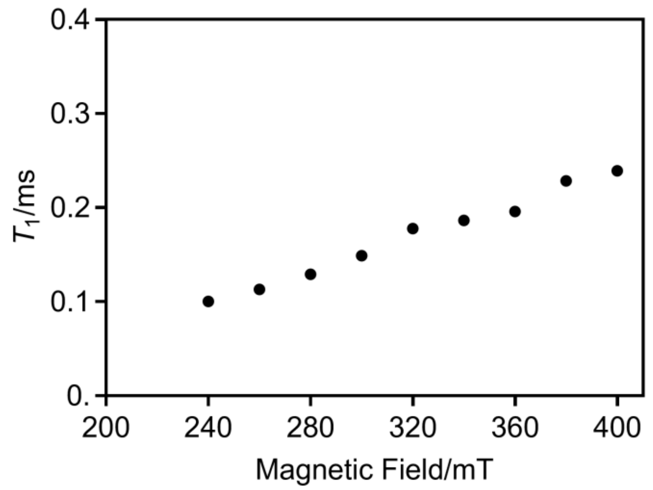

Supplementary Fig. 29.

Spin-lattice relaxation time T_1 of **1** observed at 4 K. The spin-lattice relaxation time T_1 was measured by the three-pulse inversion recovery method. The value of T_1 gradually decreases when the magnetic field going down, indicating the spin-orbit interaction on the Co ion contribute to the acceleration of T_1.

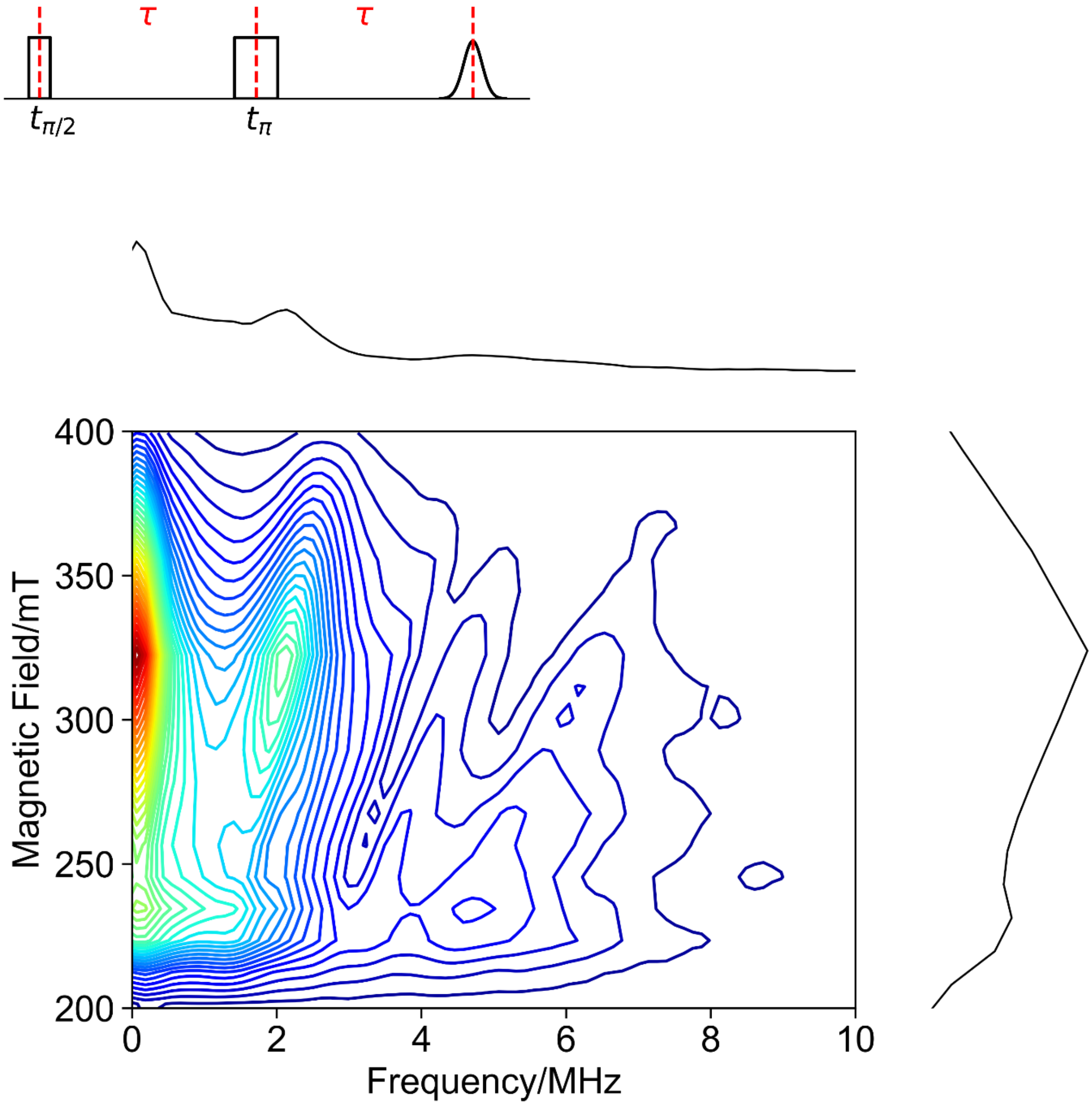

Supplementary Fig. 30.
Field-swept two-pulse ESEEM spectra of **1** observed at 4 K. Experimental setting: $t_{\pi/2} = 12$ ns and $t_\pi = 22$ ns.

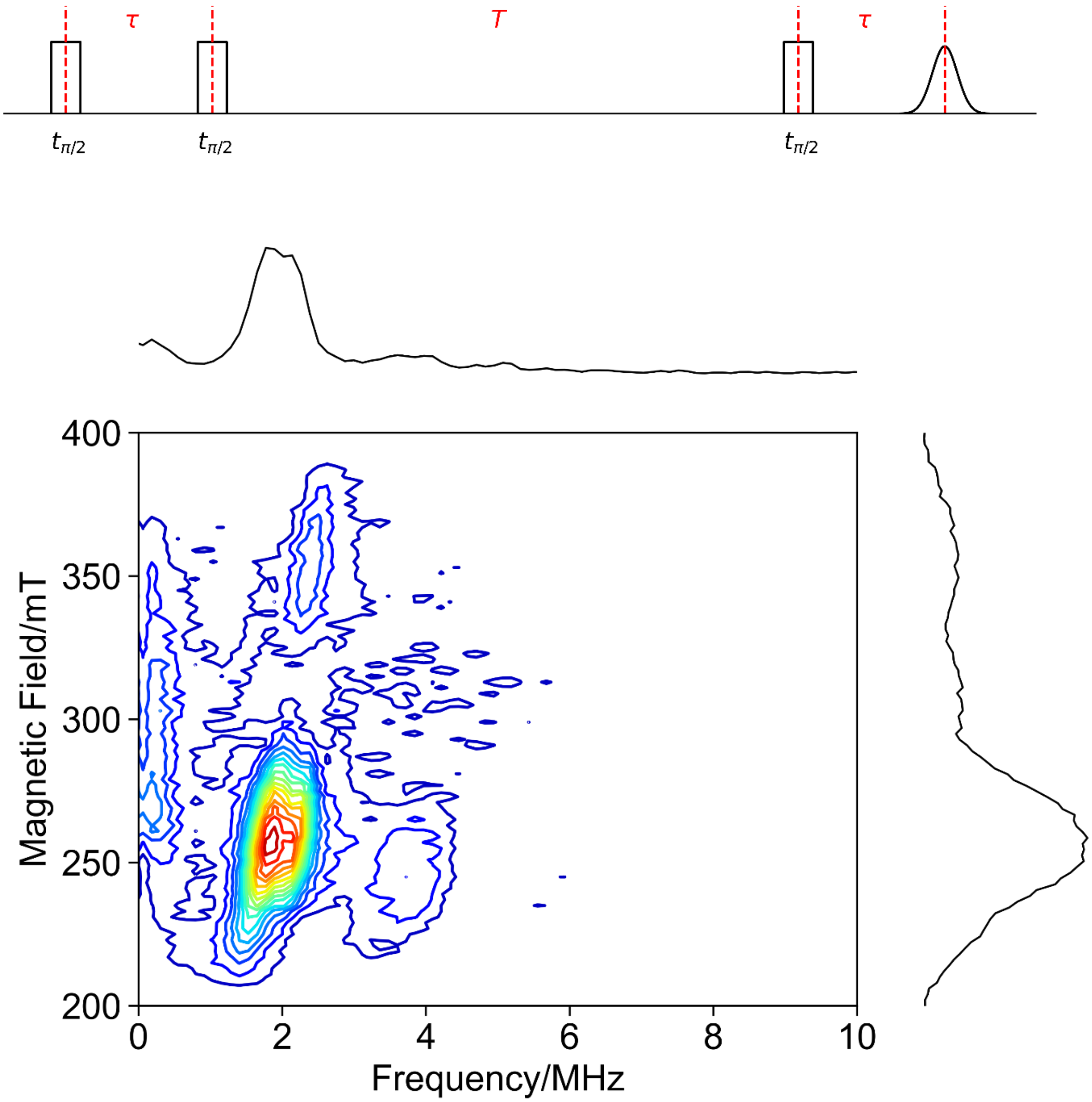

Supplementary Fig. 31.
Field-swept three-pulse ESEEM spectra of **1** observed at 4 K. Experimental setting: $t_{\pi/2} = 12$ ns, $t_{\pi} = 22$ ns, and $\tau = 476$ ns.

(a) Observed at 320 mT

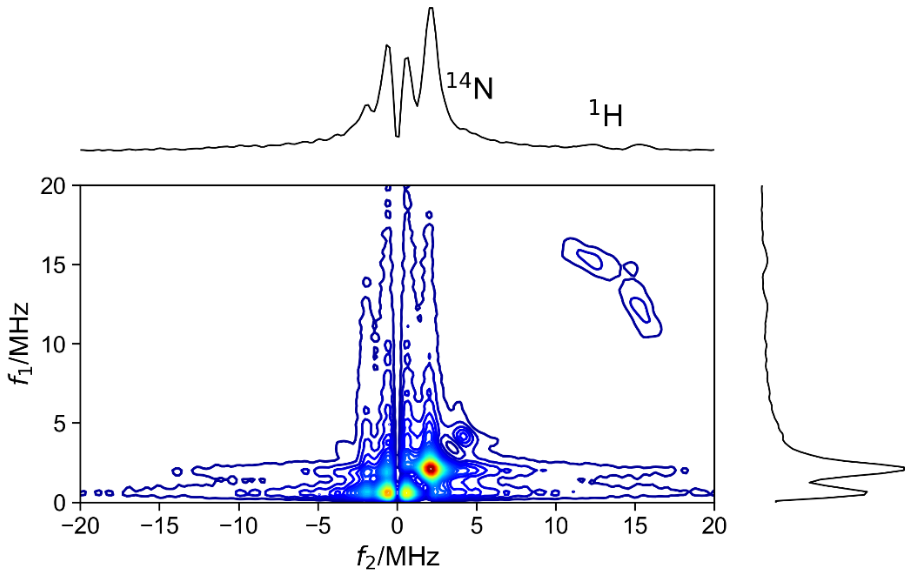

(b) Simulation

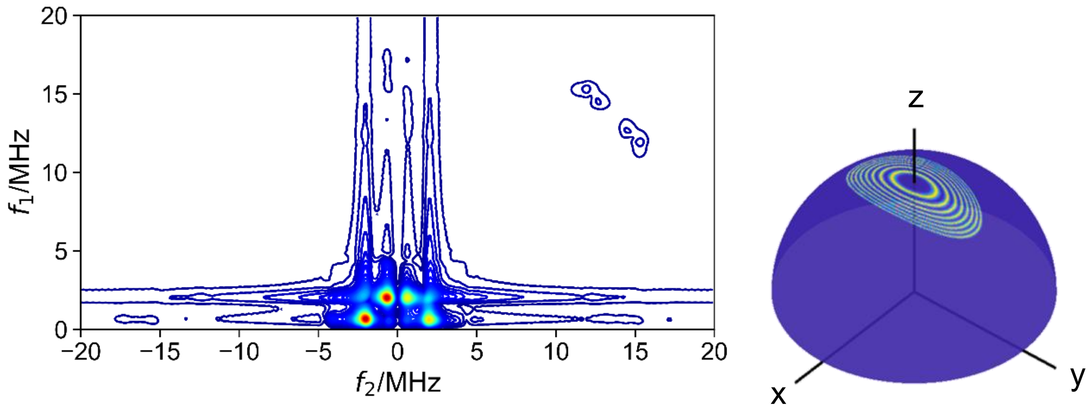

Supplementary Fig. 32.

Four-pulse HYSCORE spectra of **1** at 4 K. (a) Contour plots observed at 320 mT. (b) Simulated spectra using the EasySpin toolbox. Orientation selectivity at 340 mT was given on the right. Only two-protons in addition to ^{14}N nucelar spin were considered for the simulation because of the reduction of the size of spin-Hamiltonian.

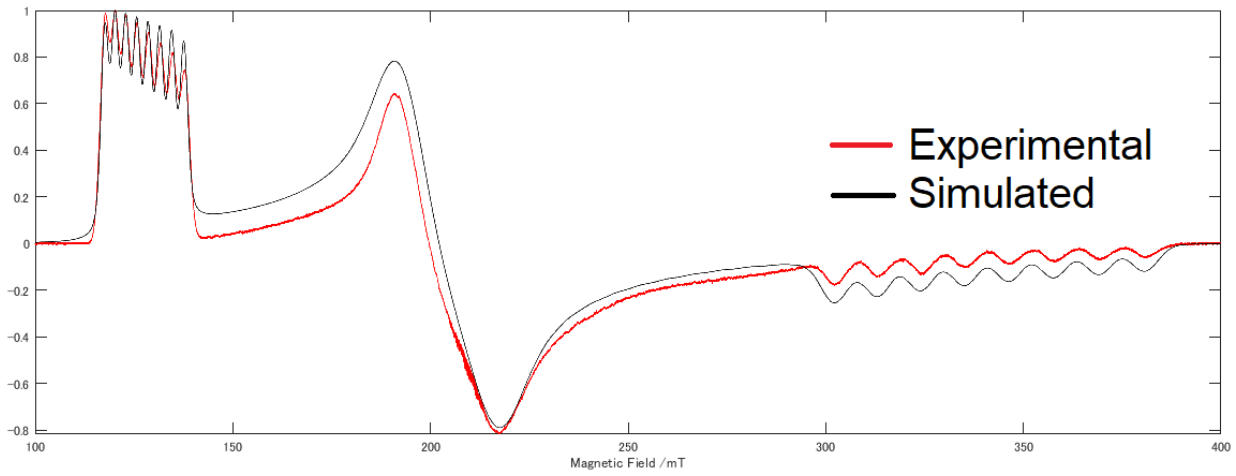

Supplementary Fig. 33.

Experimental (4.2 K, toluene glass) and simulated EPR spectra of **1** using S = 3/2 and D and E values (See Supplementary Table 1).

Supplementary Table 1.

Spin Hamiltonian parameters of **1**. Hyperfine coupling parameters of nitrogen atom and protons were determined from the observed HYSCORE spectra.

	Exp.(EPR)	Exp.(EPR)
S	1/2 (fictitious spin)	3/2
g_{xx}	5.077	2.10
g_{yy}	3.172	2.09
g_{zz}	1.896	2.04
D /cm^{-1}	–	>5
E /cm^{-1}	–	>0.79
E/D	–	-0.156
D_{xx} /cm^{-1}	–	< -2.45
D_{yy} /cm^{-1}	–	< -0.88
D_{zz} /cm^{-1}	–	>3.33
A_{xx}^{Co} /MHz	205	84
A_{yy}^{Co} /MHz	142	90
A_{zz}^{Co} /MHz	293	320
A_{xx}^{N} /MHz	1	–
A_{yy}^{N} /MHz	0.9	–
A_{zz}^{N} /MHz	3	–
$A_{x'x'}^{\mathrm{H(Cp)}}$ /MHz		–
$A_{y'y'}^{\mathrm{H(Cp)}}$ /MHz	$a_{\mathrm{iso}}^{\mathrm{H(Cp)}} \approx$ 3 ~ 5 /MHz[a]	–
$A_{z'z'}^{\mathrm{H(Cp)}}$ /MHz		–

[a] The magnitude of $a_{\mathrm{iso}}^{\mathrm{H(Cp)}}$ was estimated from the spread of the HYSCORE signals.

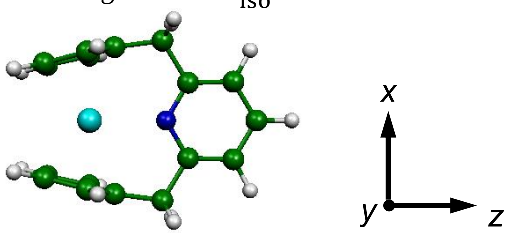

4. Quantum crystallography and SC-XRD studies

Supplementary Table 2.
List of crystallographically determined Co–N bond distances of Co^{II}PNP pincer complexes.

CCDC number	Co–N bond distance (Å)	DOI
1577859	2.154(3), 2.183(4)	10.1021/jacs.6b13346
1022626	2.252(2), 2.2703(19)	10.1021/ic5021725, 10.1039/C6DT03461F
2103902	2.0074(8)	10.1021/acs.organomet.1c00488
2103903	2.342(3), 2.343(2)	10.1021/acs.organomet.1c00488
1488153	2.1779(19)	10.1021/acscentsci.6b00283
1536880	1.974(8)	10.1021/acscentsci.6b00283
181873	2.097(5)	10.1002/1521-3749(200213)628:13<2839::AID-ZAAC2839>3.0.CO;2-9
181874	2.057(6)	10.1002/1521-3749(200213)628:13<2839::AID-ZAAC2839>3.0.CO;2-9
982447	2.018(3)	10.1039/C4SC00255E
982448	2.0831(17)	10.1039/C4SC00255E
2106382	2.0528(12)	10.1021/acs.organomet.1c00499

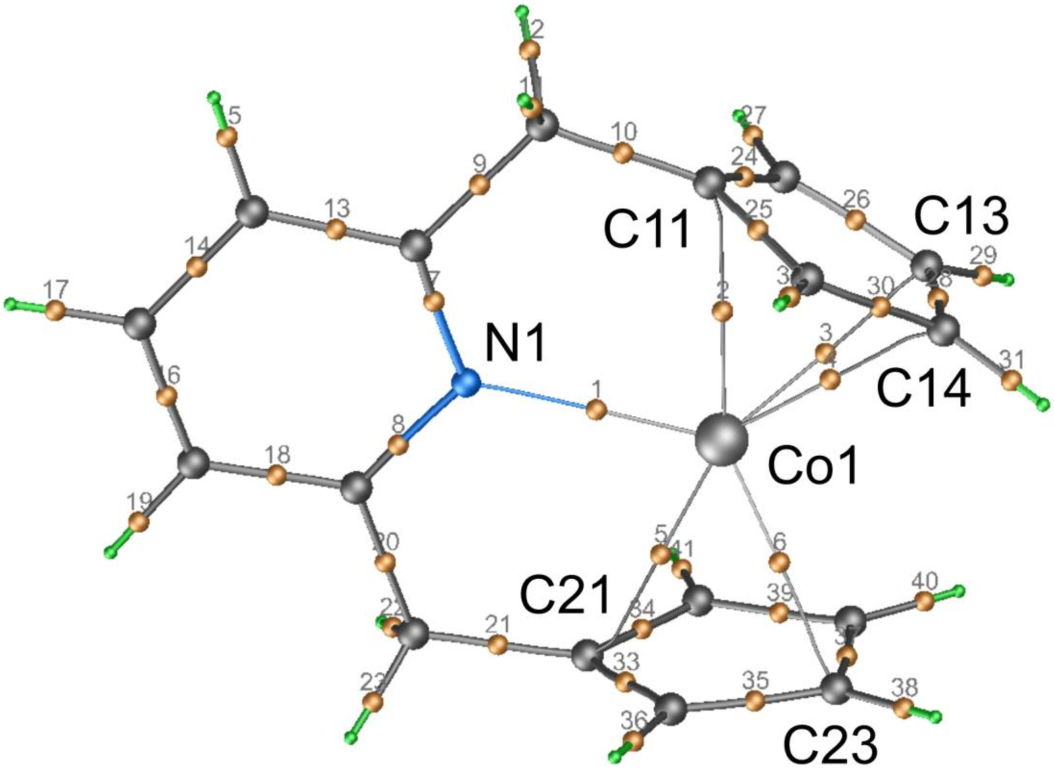

Supplementary Fig. 34.
Molecular graph for **1** in the crystal according to experimental multipole-derived electron density $\rho(\mathbf{r})$. Bond critical points (3, –1) are denoted by brown dots.

Summary of bond critical points (3, –1) for 1 in the crystal according to experimental multipole-derived electron density (WinXPRO)

--- kinetic energy approximation: Kinetic gradient energy (Kirzhnits, 1957) ---

CP number CP type Bond Function xa ya za xf yf zf
R

1 (3,-1) Co1 1 - N1 2 RHO 6.0910 2.0825 2.5351 0.8095 0.1209 0.2927
1.076

rho(r) = 0.057207 a.u. lambda_1 = -0.0619 a.u.

d2rho(r) = 0.219965 a.u. lambda_2 = -0.0572 a.u.

g(r) = 0.061047 a.u. lambda_3 = 0.3391 a.u.

v(r) = -0.067104 a.u. ellipticity = 0.0817
h(r) = -0.006056 a.u.

2 (3,-1) Co1 1 - C11 10 RHO 4.7315 2.0990 2.3556 0.6427 0.1219 0.2720 1.140
rho(r) = 0.054772 a.u. lambda_1 = -0.0475 a.u.
d2rho(r) = 0.152899 a.u. lambda_2 = -0.0080 a.u.
g(r) = 0.048164 a.u. lambda_3 = 0.2084 a.u.
v(r) = -0.058104 a.u. ellipticity = 4.9297
h(r) = -0.009940 a.u.

3 (3,-1) Co1 1 - C13 12 RHO 4.5167 1.9381 1.3438 0.5810 0.1126 0.1552 1.129
rho(r) = 0.054558 a.u. lambda_1 = -0.0434 a.u.
d2rho(r) = 0.167918 a.u. lambda_2 = -0.0106 a.u.
g(r) = 0.050520 a.u. lambda_3 = 0.2220 a.u.
v(r) = -0.059061 a.u. ellipticity = 3.0991
h(r) = -0.008541 a.u.

4 (3,-1) Co1 1 - C14 13 RHO 4.5088 2.5702 1.4237 0.5829 0.1493 0.1644 1.135
rho(r) = 0.053289 a.u. lambda_1 = -0.0428 a.u.
d2rho(r) = 0.167577 a.u. lambda_2 = -0.0041 a.u.
g(r) = 0.049597 a.u. lambda_3 = 0.2145 a.u.
v(r) = -0.057299 a.u. ellipticity = 9.3390
h(r) = -0.007702 a.u.

5 (3,-1) Co1 1 - C21 15 RHO 6.6702 2.3351 1.3028 0.8335 0.1356 0.1504 1.139
rho(r) = 0.054329 a.u. lambda_1 = -0.0478 a.u.
d2rho(r) = 0.155898 a.u. lambda_2 = -0.0072 a.u.

g(r) = 0.048359 a.u. lambda_3 = 0.2109 a.u.

v(r) = -0.057744 a.u. ellipticity = 5.6777

h(r) = -0.009385 a.u.

6 (3,-1) Co1 1 - C23 17 RHO 5.9712 2.7134 0.6573 0.7278 0.1576 0.0759 1.133

rho(r) = 0.053622 a.u. lambda_1 = -0.0451 a.u.

d2rho(r) = 0.161939 a.u. lambda_2 = -0.0086 a.u.

g(r) = 0.048883 a.u. lambda_3 = 0.2157 a.u.

v(r) = -0.057281 a.u. ellipticity = 4.2546

h(r) = -0.008398 a.u.

7 (3,-1) N1 2 - C2 4 RHO 6.2464 1.9383 4.2277 0.8888 0.1126 0.4882 0.821

rho(r) = 0.334632 a.u. lambda_1 = -0.7396 a.u.

d2rho(r) = -1.018242 a.u. lambda_2 = -0.6443 a.u.

g(r) = 0.293398 a.u. lambda_3 = 0.3657 a.u.

v(r) = -0.841357 a.u. ellipticity = 0.1479

h(r) = -0.547959 a.u.

8 (3,-1) N1 2 - C6 8 RHO 7.4592 1.8815 3.5348 1.0069 0.1093 0.4082 0.823

rho(r) = 0.334096 a.u. lambda_1 = -0.7416 a.u.

d2rho(r) = -1.031917 a.u. lambda_2 = -0.6495 a.u.

g(r) = 0.289885 a.u. lambda_3 = 0.3592 a.u.

v(r) = -0.837749 a.u. ellipticity = 0.1418

h(r) = -0.547864 a.u.

9 (3,-1) C1 3 - C2 4 RHO 5.2060 1.9769 4.6571 0.7815 0.1148 0.5378 0.747

rho(r) = 0.245882 a.u. lambda_1 = -0.4728 a.u.

d2rho(r) = -0.493361 a.u. lambda_2 = -0.4252 a.u.

g(r) = 0.194858 a.u. lambda_3 = 0.4046 a.u.

v(r) = -0.513057 a.u. ellipticity = 0.1120

h(r) = -0.318198 a.u.

10 (3,-1) C1 3 - C11 10 RHO 4.2055 2.0665 3.9644 0.6386 0.1200 0.4578 0.721

rho(r) = 0.262521 a.u. lambda_1 = -0.4824 a.u.

d2rho(r) = -0.581526 a.u. lambda_2 = -0.4760 a.u.

g(r) = 0.212115 a.u. lambda_3 = 0.3768 a.u.

v(r) = -0.569611 a.u. ellipticity = 0.0135

h(r) = -0.357496 a.u.

11 (3,-1) C1 3 - H1A 31 RHO 4.2542 2.5859 5.0280 0.6827 0.1502 0.5806 0.717

rho(r) = 0.251816 a.u. lambda_1 = -0.6252 a.u.

d2rho(r) = -0.591735 a.u. lambda_2 = -0.5765 a.u.

g(r) = 0.189697 a.u. lambda_3 = 0.6100 a.u.

v(r) = -0.527329 a.u. ellipticity = 0.0846

h(r) = -0.337631 a.u.

12 (3,-1) C1 3 - H1B 32 RHO 4.1776 1.4419 4.9561 0.6710 0.0837 0.5723 0.719

rho(r) = 0.251217 a.u. lambda_1 = -0.6246 a.u.

d2rho(r) = -0.595199 a.u. lambda_2 = -0.5752 a.u.

g(r) = 0.187978 a.u. lambda_3 = 0.6046 a.u.

v(r) = -0.524755 a.u. ellipticity = 0.0860

h(r) = -0.336778 a.u.

13 (3,-1) C2 4 - C3 5 RHO 6.2902 1.9047 5.2754 0.9317 0.1106 0.6092 0.677

rho(r) = 0.317740 a.u. lambda_1 = -0.6743 a.u.

d2rho(r) = -0.862852 a.u. lambda_2 = -0.5526 a.u.

g(r) = 0.280996 a.u. lambda_3 = 0.3641 a.u.

v(r) = -0.777705 a.u. ellipticity = 0.2202

h(r) = -0.496709 a.u.

14 (3,-1) C3 5 - C4 6 RHO 7.3119 1.8331 5.9167 1.0752 0.1065 0.6832 0.685

rho(r) = 0.326871 a.u. lambda_1 = -0.6973 a.u.

d2rho(r) = -0.902572 a.u. lambda_2 = -0.5773 a.u.

g(r) = 0.294916 a.u. lambda_3 = 0.3721 a.u.

v(r) = -0.815475 a.u. ellipticity = 0.2080

h(r) = -0.520559 a.u.

15 (3,-1) C3 5 - H3 28 RHO 6.2492 1.8590 6.5213 0.9717 0.1080 0.7531 0.721

rho(r) = 0.272059 a.u. lambda_1 = -0.7222 a.u.

d2rho(r) = -0.800326 a.u. lambda_2 = -0.6809 a.u.

g(r) = 0.194588 a.u. lambda_3 = 0.6028 a.u.

v(r) = -0.589257 a.u. ellipticity = 0.0607

h(r) = -0.394669 a.u.

16 (3,-1) C4 6 - C5 7 RHO 8.3644 1.8008 5.3151 1.1777 0.1046 0.6138 0.705

rho(r) = 0.327026 a.u. lambda_1 = -0.6969 a.u.

d2rho(r) = -0.899328 a.u. lambda_2 = -0.5741 a.u.

g(r) = 0.295808 a.u. lambda_3 = 0.3716 a.u.

v(r) = -0.816449 a.u. ellipticity = 0.2139

h(r) = -0.520640 a.u.

17 (3,-1) C4 6 - H4 29 RHO 8.3780 1.7765 6.5399 1.2234 0.1032 0.7552 0.712

rho(r) = 0.266403 a.u. lambda_1 = -0.6797 a.u.

d2rho(r) = -0.749195 a.u. lambda_2 = -0.6538 a.u.

g(r) = 0.191824 a.u. lambda_3 = 0.5842 a.u.

v(r) = -0.570948 a.u. ellipticity = 0.0396

h(r) = -0.379123 a.u.

18 (3,-1) C5 7 - C6 8 RHO 8.3327 1.8137 4.1065 1.1304 0.1053 0.4742 0.716

rho(r) = 0.317735 a.u. lambda_1 = -0.6764 a.u.

d2rho(r) = -0.862881 a.u. lambda_2 = -0.5491 a.u.

g(r) = 0.280980 a.u. lambda_3 = 0.3626 a.u.

v(r) = -0.777680 a.u. ellipticity = 0.2317

h(r) = -0.496700 a.u.

19 (3,-1) C5 7 - H5 30 RHO 9.4208 1.7669 4.7006 1.2801 0.1026 0.5428 0.720

rho(r) = 0.270231 a.u. lambda_1 = -0.7140 a.u.

d2rho(r) = -0.791284 a.u. lambda_2 = -0.6745 a.u.

g(r) = 0.192429 a.u. lambda_3 = 0.5972 a.u.

v(r) = -0.582680 a.u. ellipticity = 0.0585

h(r) = -0.390250 a.u.

20 (3,-1) C6 8 - C7 9 RHO 8.3578 1.8256 2.8594 1.0885 0.1060 0.3302 0.765

rho(r) = 0.249987 a.u. lambda_1 = -0.4846 a.u.

d2rho(r) = -0.517733 a.u. lambda_2 = -0.4360 a.u.

g(r) = 0.198549 a.u. lambda_3 = 0.4029 a.u.

v(r) = -0.526531 a.u. ellipticity = 0.1113

h(r) = -0.327982 a.u.

21 (3,-1) C7 9 - C21 15 RHO 8.2840 1.9727 1.6506 1.0363 0.1146 0.1906 0.730

rho(r) = 0.261519 a.u. lambda_1 = -0.4938 a.u.

d2rho(r) = -0.588563 a.u. lambda_2 = -0.4691 a.u.

g(r) = 0.208979 a.u. lambda_3 = 0.3744 a.u.

v(r) = -0.565098 a.u. ellipticity = 0.0528

h(r) = -0.356120 a.u.

22 (3,-1) C7 9 - H7A 33 RHO 8.9988 1.1445 2.1177 1.1374 0.0665 0.2445 0.718

rho(r) = 0.252709 a.u. lambda_1 = -0.6267 a.u.

d2rho(r) = -0.607083 a.u. lambda_2 = -0.5863 a.u.

g(r) = 0.188846 a.u. lambda_3 = 0.6059 a.u.

v(r) = -0.529463 a.u. ellipticity = 0.0688

h(r) = -0.340617 a.u.

23 (3,-1) C7 9 - H7B 34 RHO 9.2808 2.2495 2.2246 1.1745 0.1306 0.2569 0.719

rho(r) = 0.256520 a.u. lambda_1 = -0.6436 a.u.

d2rho(r) = -0.628620 a.u. lambda_2 = -0.5969 a.u.

g(r) = 0.192583 a.u. lambda_3 = 0.6119 a.u.

v(r) = -0.542320 a.u. ellipticity = 0.0782

h(r) = -0.349738 a.u.

24 (3,-1) C11 10 - C12 11 RHO 3.8471 1.5696 2.8387 0.5558 0.0912 0.3278 0.689

rho(r) = 0.296902 a.u. lambda_1 = -0.5993 a.u.

d2rho(r) = -0.737477 a.u. lambda_2 = -0.4775 a.u.

g(r) = 0.256482 a.u. lambda_3 = 0.3393 a.u.

v(r) = -0.697332 a.u. ellipticity = 0.2552

h(r) = -0.440851 a.u.

25 (3,-1) C11 10 - C15 14 RHO 3.8328 2.7199 2.8935 0.5561 0.1580 0.3341 0.704

rho(r) = 0.301581 a.u. lambda_1 = -0.6175 a.u.

d2rho(r) = -0.779266 a.u. lambda_2 = -0.4932 a.u.

g(r) = 0.259534 a.u. lambda_3 = 0.3314 a.u.

v(r) = -0.713885 a.u. ellipticity = 0.2519

h(r) = -0.454351 a.u.

26 (3,-1) C12 11 - C13 12 RHO 3.6145 1.2608 1.7185 0.4881 0.0732 0.1984 0.698

rho(r) = 0.296906 a.u. lambda_1 = -0.6010 a.u.

d2rho(r) = -0.727113 a.u. lambda_2 = -0.4842 a.u.

g(r) = 0.258217 a.u. lambda_3 = 0.3581 a.u.

v(r) = -0.698212 a.u. ellipticity = 0.2411

h(r) = -0.439995 a.u.

27 (3,-1) C12 11 - H12 20 RHO 3.8366 0.3113 2.5349 0.5437 0.0181 0.2927 0.738

rho(r) = 0.263584 a.u. lambda_1 = -0.6860 a.u.

d2rho(r) = -0.762034 a.u. lambda_2 = -0.6630 a.u.

g(r) = 0.184119 a.u. lambda_3 = 0.5869 a.u.

v(r) = -0.558746 a.u. ellipticity = 0.0347

h(r) = -0.374627 a.u.

28 (3,-1) C13 12 - C14 13 RHO 3.4475 2.2729 1.1033 0.4463 0.1320 0.1274 0.730

rho(r) = 0.299853 a.u. lambda_1 = -0.6139 a.u.

d2rho(r) = -0.772983 a.u. lambda_2 = -0.5089 a.u.

g(r) = 0.256869 a.u. lambda_3 = 0.3498 a.u.

v(r) = -0.706985 a.u. ellipticity = 0.2063

h(r) = -0.450115 a.u.

29 (3,-1) C13 12 - H13 21 RHO 3.3311 1.1426 0.4693 0.4097 0.0664 0.0542 0.737

rho(r) = 0.264939 a.u. lambda_1 = -0.6978 a.u.

d2rho(r) = -0.776290 a.u. lambda_2 = -0.6634 a.u.

g(r) = 0.184412 a.u. lambda_3 = 0.5849 a.u.

v(r) = -0.562897 a.u. ellipticity = 0.0519

h(r) = -0.378485 a.u.

30 (3,-1) C14 13 - C15 14 RHO 3.5862 3.1462 1.8081 0.4880 0.1827 0.2088 0.678

rho(r) = 0.300559 a.u. lambda_1 = -0.6322 a.u.

d2rho(r) = -0.795497 a.u. lambda_2 = -0.5019 a.u.

g(r) = 0.254632 a.u. lambda_3 = 0.3386 a.u.

v(r) = -0.708139 a.u. ellipticity = 0.2597

h(r) = -0.453506 a.u.

31 (3,-1) C14 13 - H14 22 RHO 3.3086 3.4334 0.6182 0.4124 0.1994 0.0714 0.739

rho(r) = 0.258982 a.u. lambda_1 = -0.6624 a.u.

d2rho(r) = -0.703950 a.u. lambda_2 = -0.6355 a.u.

g(r) = 0.184799 a.u. lambda_3 = 0.5939 a.u.

v(r) = -0.545586 a.u. ellipticity = 0.0423

h(r) = -0.360787 a.u.

32 (3,-1) C15 14 - H15 23 RHO 3.7779 3.9935 2.8041 0.5464 0.2319 0.3238 0.738

rho(r) = 0.258232 a.u. lambda_1 = -0.6562 a.u.

d2rho(r) = -0.699158 a.u. lambda_2 = -0.6354 a.u.

g(r) = 0.184140 a.u. lambda_3 = 0.5925 a.u.

v(r) = -0.543070 a.u. ellipticity = 0.0326

h(r) = -0.358930 a.u.

33 (3,-1) C21 15 - C22 16 RHO 7.6109 2.7821 0.9196 0.9306 0.1616 0.1062 0.702

rho(r) = 0.301537 a.u. lambda_1 = -0.6107 a.u.

d2rho(r) = -0.754915 a.u. lambda_2 = -0.4898 a.u.

g(r) = 0.263498 a.u. lambda_3 = 0.3456 a.u.

v(r) = -0.715724 a.u. ellipticity = 0.2467
h(r) = -0.452226 a.u.

34 (3,-1) C21 15 - C25 19 RHO 7.4991 1.6587 0.6676 0.9083 0.0963 0.0771 0.698
rho(r) = 0.303078 a.u. lambda_1 = -0.5962 a.u.
d2rho(r) = -0.764879 a.u. lambda_2 = -0.5105 a.u.
g(r) = 0.265158 a.u. lambda_3 = 0.3419 a.u.
v(r) = -0.721536 a.u. ellipticity = 0.1678
h(r) = -0.456378 a.u.

35 (3,-1) C22 16 - C23 17 RHO 6.8669 3.3827 0.2276 0.8180 0.1965 0.0263 0.706
rho(r) = 0.303259 a.u. lambda_1 = -0.6194 a.u.
d2rho(r) = -0.750876 a.u. lambda_2 = -0.4949 a.u.
g(r) = 0.267882 a.u. lambda_3 = 0.3634 a.u.
v(r) = -0.723483 a.u. ellipticity = 0.2516
h(r) = -0.455601 a.u.

36 (3,-1) C22 16 - H22 24 RHO 7.5756 4.0590 1.0581 0.9314 0.2357 0.1222 0.739
rho(r) = 0.259828 a.u. lambda_1 = -0.6731 a.u.
d2rho(r) = -0.726722 a.u. lambda_2 = -0.6435 a.u.
g(r) = 0.182650 a.u. lambda_3 = 0.5898 a.u.
v(r) = -0.546981 a.u. ellipticity = 0.0459
h(r) = -0.364331 a.u.

37 (3,-1) C23 17 - C24 18 RHO 6.2898 2.6060 -0.4491 0.7255 0.1513 -0.0519 0.712
rho(r) = 0.299613 a.u. lambda_1 = -0.6154 a.u.
d2rho(r) = -0.754547 a.u. lambda_2 = -0.4954 a.u.

g(r) = 0.259427 a.u. lambda_3 = 0.3562 a.u.

v(r) = -0.707490 a.u. ellipticity = 0.2422

h(r) = -0.448063 a.u.

38 (3,-1) C23 17 - H23 25 RHO 5.9795 3.8396 -0.5903 0.6839 0.2230 -0.0682 0.742

rho(r) = 0.267855 a.u. lambda_1 = -0.7060 a.u.

d2rho(r) = -0.793386 a.u. lambda_2 = -0.6807 a.u.

g(r) = 0.187341 a.u. lambda_3 = 0.5933 a.u.

v(r) = -0.573028 a.u. ellipticity = 0.0372

h(r) = -0.385687 a.u.

39 (3,-1) C24 18 - C25 19 RHO 6.6597 1.5556 -0.1900 0.7785 0.0903 -0.0219 0.672

rho(r) = 0.296975 a.u. lambda_1 = -0.6298 a.u.

d2rho(r) = -0.808882 a.u. lambda_2 = -0.5027 a.u.

g(r) = 0.244735 a.u. lambda_3 = 0.3236 a.u.

v(r) = -0.691691 a.u. ellipticity = 0.2530

h(r) = -0.446956 a.u.

40 (3,-1) C24 18 - H24 26 RHO 5.7669 1.6135 -1.0575 0.6420 0.0937 -0.1221 0.738

rho(r) = 0.263673 a.u. lambda_1 = -0.6768 a.u.

d2rho(r) = -0.750241 a.u. lambda_2 = -0.6614 a.u.

g(r) = 0.186258 a.u. lambda_3 = 0.5879 a.u.

v(r) = -0.560077 a.u. ellipticity = 0.0233

h(r) = -0.373819 a.u.

41 (3,-1) C25 19 - H25 27 RHO 7.2122 0.4524 0.2680 0.8601 0.0263 0.0310 0.741

rho(r) = 0.264241 a.u. lambda_1 = -0.6839 a.u.

d2rho(r) =	-0.754370 a.u.	lambda_2 =	-0.6666 a.u.
g(r) =	0.186689 a.u.	lambda_3 =	0.5961 a.u.
v(r) =	-0.561972 a.u.	ellipticity =	0.0258
h(r) =	-0.375282 a.u.		

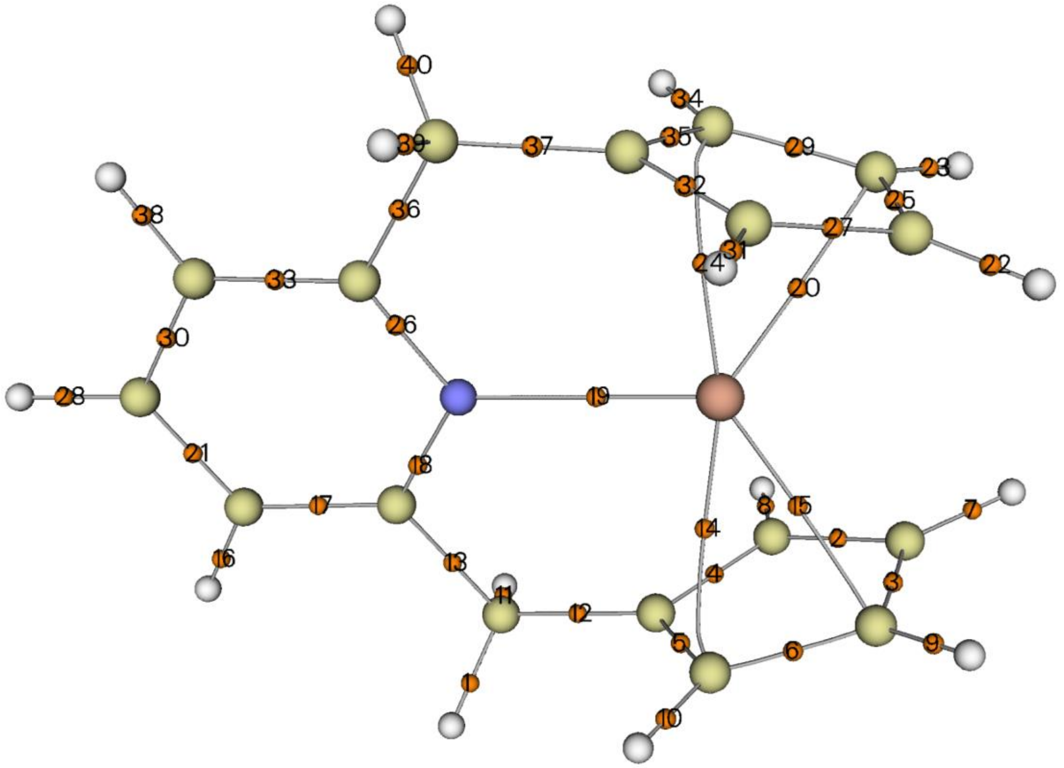

Supplementary Fig. 35.
Molecular graph for optimized free molecule **1** computed on the wave function (TPSSh-D4/def2-QZVPP//TPSS-D4/def2-TZVPP). Bond critical points (3, –1) are denoted by orange dots.

Summary of selected bond critical points (3, –1) for optimized free molecule 1 computed on the wave function (TPSSh-D4/def2-QZVPP//TPSS-D4/def2-TZVPP; Multiwfn)

--- Note: Unless otherwise specified, all units are in a.u. ---

---------------- CP 19, Type (3,-1) ----------------

Position (Bohr): 0.000000000000 0.000000000000 -0.156640723951

Position (Angstrom): 0.000000000000 0.000000000000 -0.082890701414

CP type: (3,-1)

Density of all electrons: 0.5530847476E-01

Density of Alpha electrons: 0.3086243734E-01

Density of Beta electrons: 0.2444603741E-01

Spin density of electrons: 0.6416399930E-02

Lagrangian kinetic energy G(r): 0.6516975498E-01

G(r) in X,Y,Z: 0.1494154661E-01 0.1024168132E-01 0.3998652706E-01

Hamiltonian kinetic energy K(r): 0.1057412719E-01

Potential energy density V(r): -0.7574388217E-01

Energy density E(r) or H(r): -0.1057412719E-01

Laplacian of electron density: 0.2183825112E+00

Electron localization function (ELF): 0.1204566662E+00

Localized orbital locator (LOL): 0.2627429123E+00

Local information entropy: 0.2898057478E-02

Interaction region indicator (IRI): 0.7545424911E-15

Reduced density gradient (RDG): 0.1000000000E+03

Reduced density gradient with promolecular approximation: 0.3001792361E+00

Sign(lambda2)*rho: -0.5530847476E-01

Sign(lambda2)*rho with promolecular approximation: -0.5514843839E-01

Corr. hole for alpha, ref.:   0.00000   0.00000   0.00000 : -0.2949731570E-01

Source function, ref.:   0.00000   0.00000   0.00000 : -0.1109438697E+00

Wavefunction value for orbital   1 : -0.1644948817E-05

Average local ionization energy (ALIE): 0.5082919793E+00

van der Waals potential (probe atom: C): 0.1393337186E+06 kcal/mol

Delta-g (under promolecular approximation): 0.7811389436E-01

Delta-g (under Hirshfeld partition): 0.1087159858E+00

User-defined real space function: 0.1000000000E+01

ESP from nuclear charges: 0.3886443015E+02

ESP from electrons: -0.3859484929E+02

Total ESP: 0.2695808595E+00 a.u. (0.7335668E+01 eV, 0.1691647E+03 kcal/mol)

Note: Below information are for electron density

Components of gradient in x/y/z are:

-0.8652466189E-18 -0.6185127326E-18 -0.3122502257E-16

Norm of gradient is: 0.3124313115E-16

Components of Laplacian in x/y/z are:
-0.4184040001E-01 -0.6116618530E-01 0.3213890965E+00
Total: 0.2183825112E+00

Hessian matrix:
-0.4184040001E-01 -0.1995579364E-02 -0.4694154682E-17
-0.1995579364E-02 -0.6116618530E-01 -0.1527592410E-16
-0.4694154682E-17 -0.1527592410E-16 0.3213890965E+00
Eigenvalues of Hessian: -0.6137009717E-01 -0.4163648815E-01 0.3213890965E+00
Eigenvectors (columns) of Hessian:
-0.1016524828E+00 0.9948199700E+00 -0.1293064423E-16
-0.9948199700E+00 -0.1016524828E+00 -0.4207946973E-16
-0.4317592890E-16 0.8586180535E-17 0.1000000000E+01
Determinant of Hessian: 0.8212247719E-03
Ellipticity of electron density: 0.473950
eta index: 0.190953

----------------   CP   14,   Type (3,-1)   ----------------
Position (Bohr):   -0.838848726099   -1.926953209989   1.551861949728
Position (Angstrom):   -0.443899629246   -1.019699725203   0.821209978263
CP type: (3,-1)
Density of all electrons: 0.5494507897E-01
Density of Alpha electrons: 0.2766406649E-01
Density of Beta electrons: 0.2728101248E-01
Spin density of electrons: 0.3830540134E-03
Lagrangian kinetic energy G(r): 0.5355879896E-01
G(r) in X,Y,Z: 0.1777170802E-01 0.2406653056E-01 0.1172056037E-01
Hamiltonian kinetic energy K(r): 0.1206242233E-01
Potential energy density V(r): -0.6562122129E-01
Energy density E(r) or H(r): -0.1206242233E-01

Laplacian of electron density: 0.1659855065E+00

Electron localization function (ELF): 0.1543673818E+00

Localized orbital locator (LOL): 0.2986032696E+00

Local information entropy: 0.2881414910E-02

Interaction region indicator (IRI): 0.1629063972E-14

Reduced density gradient (RDG): 0.1000000000E+03

Reduced density gradient with promolecular approximation: 0.6981086980E-01

Sign(lambda2)*rho: -0.5494507897E-01

Sign(lambda2)*rho with promolecular approximation: -0.6269266814E-01

Corr. hole for alpha, ref.:   0.00000   0.00000   0.00000 : -0.2694495415E-03

Source function, ref.:   0.00000   0.00000   0.00000 : -0.5055988403E-02

Wavefunction value for orbital   1 : 0.2388046829E-05

Average local ionization energy (ALIE): 0.4734915596E+00

van der Waals potential (probe atom: C): 0.1658305945E+06 kcal/mol

Delta-g (under promolecular approximation): 0.9960010560E-01

Delta-g (under Hirshfeld partition): 0.1082247506E+00

User-defined real space function: 0.1000000000E+01

ESP from nuclear charges: 0.3776961093E+02

ESP from electrons: -0.3751817665E+02

Total ESP: 0.2514342873E+00 a.u. (0.6841875E+01 eV, 0.1577775E+03 kcal/mol)

Note: Below information are for electron density

Components of gradient in x/y/z are:

-0.4857225733E-16 0.3469446952E-17 0.4597017211E-16

Norm of gradient is: 0.6696684230E-16

Components of Laplacian in x/y/z are:

0.3839748047E-01 0.1559121167E+00 -0.2832409061E-01

Total: 0.1659855065E+00

Hessian matrix:

0.3839748047E-01 0.1002490334E+00 -0.4527647405E-02

0.1002490334E+00 0.1559121167E+00 0.3442952295E-01

-0.4527647405E-02 0.3442952295E-01 -0.2832409061E-01

Eigenvalues of Hessian: -0.4706226487E-01 -0.3447010877E-02 0.2164947823E+00

Eigenvectors (columns) of Hessian:

-0.4772285498E+00 0.7326917968E+00 -0.4851964985E+00

0.3708430866E+00 -0.3326445701E+00 -0.8670772717E+00

-0.7966983848E+00 -0.5937257961E+00 -0.1129662016E+00

Determinant of Hessian: 0.3512067962E-04

Ellipticity of electron density: 12.653065

eta index: 0.217383

---------------- CP 15, Type (3,-1) ----------------

Position (Bohr): -0.827355286483 -1.491619215454 3.030327128097

Position (Angstrom): -0.437817562927 -0.789330896164 1.603580057770

CP type: (3,-1)

Density of all electrons: 0.5617052917E-01

Density of Alpha electrons: 0.2845788515E-01

Density of Beta electrons: 0.2771264402E-01

Spin density of electrons: 0.7452411310E-03

Lagrangian kinetic energy G(r): 0.5405357003E-01

G(r) in X,Y,Z: 0.1678862909E-01 0.1922180739E-01 0.1804313355E-01

Hamiltonian kinetic energy K(r): 0.1282038786E-01

Potential energy density V(r): -0.6687395789E-01

Energy density E(r) or H(r): -0.1282038786E-01

Laplacian of electron density: 0.1649327287E+00

Electron localization function (ELF): 0.1624201079E+00

Localized orbital locator (LOL): 0.3044239514E+00

Local information entropy: 0.2937474236E-02

Interaction region indicator (IRI): 0.2024978629E-14

Reduced density gradient (RDG): 0.1000000000E+03

Reduced density gradient with promolecular approximation: 0.7118390864E-01

Sign(lambda2)*rho: -0.5617052917E-01

Sign(lambda2)*rho with promolecular approximation: -0.6357423065E-01

Corr. hole for alpha, ref.:   0.00000   0.00000   0.00000 : -0.7137733889E-06

Source function, ref.:   0.00000   0.00000   0.00000 : -0.3774348832E-02

Wavefunction value for orbital   1 : 0.2479466118E-05

Average local ionization energy (ALIE): 0.4753041637E+00

van der Waals potential (probe atom: C): 0.2012261673E+06 kcal/mol

Delta-g (under promolecular approximation): 0.1026687584E+00

Delta-g (under Hirshfeld partition): 0.1116724154E+00

User-defined real space function: 0.1000000000E+01

ESP from nuclear charges: 0.3701607775E+02

ESP from electrons: -0.3675246736E+02

Total ESP: 0.2636103835E+00 a.u. (0.7173203E+01 eV, 0.1654182E+03 kcal/mol)

Note: Below information are for electron density

Components of gradient in x/y/z are:

0.3122502257E-16 -0.4727121472E-16 -0.6375108774E-16

Norm of gradient is: 0.8528640550E-16

Components of Laplacian in x/y/z are:

0.2347789279E-01 0.8645623907E-01 0.5499859685E-01

Total: 0.1649327287E+00

Hessian matrix:

0.2347789279E-01 0.7778360513E-01 -0.4854245258E-01

0.7778360513E-01 0.8645623907E-01 -0.1070159803E+00

-0.4854245258E-01 -0.1070159803E+00 0.5499859685E-01

Eigenvalues of Hessian: -0.4394592621E-01 -0.1179858727E-01 0.2206772422E+00

Eigenvectors (columns) of Hessian:

0.4128777843E+00 0.8088365805E+00 -0.4187067246E+00

-0.7121907015E+00 0.1501523937E-03 -0.7019860271E+00

-0.5677291079E+00 0.5880334714E+00 0.5761078862E+00

Determinant of Hessian: 0.1144211160E-03

Ellipticity of electron density: 2.724677

eta index: 0.199141

---------------- CP 20, Type (3,-1) ----------------

Position (Bohr): 0.827355286483 1.491619215454 3.030327128097

Position (Angstrom): 0.437817562927 0.789330896164 1.603580057770

CP type: (3,-1)

Density of all electrons: 0.5617052917E-01

Density of Alpha electrons: 0.2845788515E-01

Density of Beta electrons: 0.2771264402E-01

Spin density of electrons: 0.7452411310E-03

Lagrangian kinetic energy G(r): 0.5405357003E-01

G(r) in X,Y,Z: 0.1678862909E-01 0.1922180739E-01 0.1804313355E-01

Hamiltonian kinetic energy K(r): 0.1282038786E-01

Potential energy density V(r): -0.6687395789E-01

Energy density E(r) or H(r): -0.1282038786E-01

Laplacian of electron density: 0.1649327287E+00

Electron localization function (ELF): 0.1624201079E+00

Localized orbital locator (LOL): 0.3044239514E+00

Local information entropy: 0.2937474236E-02

Interaction region indicator (IRI): 0.1252346040E-14

Reduced density gradient (RDG): 0.1000000000E+03

Reduced density gradient with promolecular approximation: 0.7118390864E-01

Sign(lambda2)*rho: -0.5617052917E-01

Sign(lambda2)*rho with promolecular approximation: -0.6357423065E-01

Corr. hole for alpha, ref.: 0.00000 0.00000 0.00000 : -0.7137733889E-06

Source function, ref.:   0.00000   0.00000   0.00000 : -0.3774348832E-02

Wavefunction value for orbital   1 : 0.2479466118E-05

Average local ionization energy (ALIE): 0.4753041637E+00

van der Waals potential (probe atom: C): 0.2012261673E+06 kcal/mol

Delta-g (under promolecular approximation): 0.1026687584E+00

Delta-g (under Hirshfeld partition): 0.1116724154E+00

User-defined real space function: 0.1000000000E+01

ESP from nuclear charges: 0.3701607775E+02

ESP from electrons: -0.3675246736E+02

Total ESP: 0.2636103835E+00 a.u. (0.7173203E+01 eV, 0.1654182E+03 kcal/mol)

Note: Below information are for electron density

Components of gradient in x/y/z are:

-0.1734723476E-16 0.4076600169E-16 -0.2862293735E-16

Norm of gradient is: 0.5274529353E-16

Components of Laplacian in x/y/z are:

0.2347789279E-01 0.8645623907E-01 0.5499859685E-01

Total: 0.1649327287E+00

Hessian matrix:

0.2347789279E-01 0.7778360513E-01 0.4854245258E-01

0.7778360513E-01 0.8645623907E-01 0.1070159803E+00

0.4854245258E-01 0.1070159803E+00 0.5499859685E-01

Eigenvalues of Hessian: -0.4394592621E-01 -0.1179858727E-01 0.2206772422E+00

Eigenvectors (columns) of Hessian:

-0.4128777843E+00 -0.8088365805E+00 0.4187067246E+00

0.7121907015E+00 -0.1501523937E-03 0.7019860271E+00

-0.5677291079E+00 0.5880334714E+00 0.5761078862E+00

Determinant of Hessian: 0.1144211160E-03

Ellipticity of electron density: 2.724677

eta index: 0.199141

---------------- CP   24,   Type (3,-1)   ----------------

Note: Unless otherwise specified, all units are in a.u.
Position (Bohr):   0.838848726099   1.926953209989   1.551861949728
Position (Angstrom):   0.443899629246   1.019699725203   0.821209978263
CP type: (3,-1)
Density of all electrons: 0.5494507897E-01
Density of Alpha electrons: 0.2766406649E-01
Density of Beta electrons: 0.2728101248E-01
Spin density of electrons: 0.3830540134E-03
Lagrangian kinetic energy G(r): 0.5355879896E-01
G(r) in X,Y,Z: 0.1777170802E-01 0.2406653056E-01 0.1172056037E-01
Hamiltonian kinetic energy K(r): 0.1206242233E-01
Potential energy density V(r): -0.6562122129E-01
Energy density E(r) or H(r): -0.1206242233E-01
Laplacian of electron density: 0.1659855065E+00
Electron localization function (ELF): 0.1543673818E+00
Localized orbital locator (LOL): 0.2986032696E+00
Local information entropy: 0.2881414910E-02
Interaction region indicator (IRI): 0.1254862404E-14
Reduced density gradient (RDG): 0.1000000000E+03
Reduced density gradient with promolecular approximation: 0.6981086980E-01
Sign(lambda2)*rho: -0.5494507897E-01
Sign(lambda2)*rho with promolecular approximation: -0.6269266814E-01
Corr. hole for alpha, ref.:   0.00000   0.00000   0.00000 : -0.2694495415E-03
Source function, ref.:   0.00000   0.00000   0.00000 : -0.5055988403E-02
Wavefunction value for orbital   1 : 0.2388046829E-05
Average local ionization energy (ALIE): 0.4734915596E+00
van der Waals potential (probe atom: C): 0.1658305945E+06 kcal/mol
Delta-g (under promolecular approximation): 0.9960010560E-01
Delta-g (under Hirshfeld partition): 0.1082247506E+00
User-defined real space function: 0.1000000000E+01
ESP from nuclear charges: 0.3776961093E+02
ESP from electrons: -0.3751817665E+02
Total ESP: 0.2514342873E+00 a.u. (0.6841875E+01 eV, 0.1577775E+03 kcal/mol)

Note: Below information are for electron density

Components of gradient in x/y/z are:
0.4336808690E-16 0.1647987302E-16 0.2255140519E-16
Norm of gradient is: 0.5158432952E-16

Components of Laplacian in x/y/z are:
 0.3839748047E-01 0.1559121167E+00 -0.2832409061E-01
Total: 0.1659855065E+00

Hessian matrix:
 0.3839748047E-01 0.1002490334E+00 0.4527647405E-02
 0.1002490334E+00 0.1559121167E+00 -0.3442952295E-01
 0.4527647405E-02 -0.3442952295E-01 -0.2832409061E-01
Eigenvalues of Hessian: -0.4706226487E-01 -0.3447010877E-02 0.2164947823E+00
Eigenvectors (columns) of Hessian:
 0.4772285498E+00 0.7326917968E+00 -0.4851964985E+00
-0.3708430866E+00 -0.3326445701E+00 -0.8670772717E+00
-0.7966983848E+00 0.5937257961E+00 0.1129662016E+00
Determinant of Hessian: 0.3512067962E-04
Ellipticity of electron density: 12.653065
eta index: 0.217383

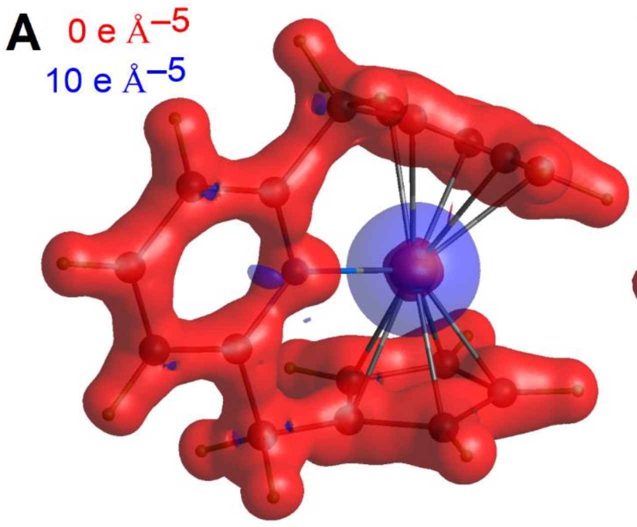

Supplementary Fig. 36.
Isosurfaces of the Laplacian of electron density $\nabla^2\rho(\mathbf{r})$ of **1** according to the diffraction (**A**) and theoretical (**B**) data. Reactive surfaces (0 e Å^{-5}) are shown in red and isosurfaces at 10 e Å^{-5} are shown in blue.

Detailed description of crystal structure determination

The X-ray diffraction experiments for **1-3** were performed on a Bruker D8 Venture diffractometer equipped with a PHOTON II CPAD detector, an IμS 3.0 microfocus X-ray source (Mo *K*α radiation). The X-ray diffraction data for the single crystal **4** were collected on a Rigaku XtaLab PRO instrument equipped with a PILATUS3 R 200K hybrid pixel array detector and MicroMax^{TM}-003 microfocus X-ray tube (Mo *K*α). Data were collected according to recommended strategies in a φ/ω (**1-3**) or ω (**4**) scan mode at 100 K. Final cell constants were determined by the global refinement of reflections from the complete data set. Images were indexed and integrated using the *APEX*4 v2021.10-0 (**1-3**) or CrysAlisPro 1.171.42.93a (**4**) data reduction package. The data were corrected for systematic errors and absorption: Numerical absorption correction based on a multifaceted crystal model and empirical absorption correction based on spherical harmonics according to the point group symmetry using equivalent reflections.

All structures were solved by the intrinsic phasing approach using *SHELXT*-2018/2 and refined by the full-matrix least-squares on F^2 using *SHELXL*-2018/3 [5,6]. Calculations were mainly performed using the *WinGX*-2021.3 suite of programs [7]. Non-hydrogen atoms were refined anisotropically. Hydrogen atoms were inserted at the calculated positions and refined as riding atoms. The disorder, if present, was resolved using free variables and reasonable restraints on geometry and anisotropic displacement parameters.

The multipole refinement of **1** was performed within the Hansen–Coppens multipole formalism as implemented in the *MoPro* software package[8]. The total electron density is considered as a superposition of pseudo-atomic electron densities, expressed as a sum of spherical core, spherical valence, and deformation valence contributions (*37*):

$$\rho_\mu(\boldsymbol{r}) = P_c\rho_c(r) + P_v\kappa^3\rho_v(\kappa r) + \sum_{l=0}^{l_{\max}} \kappa'^3 R_l(\kappa' r) \sum_{m=-l}^{+l} P_{lm}Y_{lm}(\boldsymbol{r}/r),$$

where P_c, P_v, and P_{lm} are the core, monopole, and multipole populations, and κ and κ' are the atomic spherical and deformation valence expansion/contraction parameters, respectively. $R_l$are the Slater-type radial functions. Y_{lm} are the atom-centered real spherical harmonics. Core and spherical valence scattering factors derived from the relativistic analytical wave functions of Su and Coppens were utilized [9]. The exponential Slater-type radial functions with radial function parameters n_l = 4, 4, 4, 4 for Co, n_l = 2, 2, 3, 4 for N and C, n_2 = 1 for H-atoms and the values of orbital exponents ζ_{Co} = 7.6496, ζ_N = 3.8106, ζ_C = 3.1303, and ζ_H = 2.0000 were used. The

multipole refinement was performed against F with reflections that satisfy the $I > 1.5\sigma(I)$ condition. A reciprocal resolution $\sin(\theta_{\max}/\lambda)$ of the data was 1.26 Å^{-1}. The function minimized in the least-squares procedure was $\sum w(|F_o| - k|F_c|)^2$ with weight w equal to $1/(3.4293\sigma^2(F_o^2))$. The first and fourth scale factors were refined. The unit cell electroneutrality constraint was imposed during the whole refinement procedure. The C–H bond distances, κ- and κ'-parameters for hydrogen pseudo-atoms, and κ'-parameters for all pseudo-atoms were constrained to the theoretical values obtained from the DFT-optimized periodic structure. The same κ'-parameter was used for all multipole levels of each pseudo-atom; P_{00} was fixed at zero. Multipole expansion was truncated at the hexadecapolar level ($l_{\max}$ = 4) for the Co1, N1, C11-C15, and C21-C25 pseudo-atoms and octupolar level ($l_{\max}$ = 3) for the C1-C7 pseudo-atoms. For each H-pseudo-atom, only a monopole P_v and a bond-oriented dipole P_{10} were refined. The anharmonic atomic motion of Co1 was modeled using the Gram–Charlier expansion of temperature factors up to the tensors of 4th rank. A block refinement of the Gram–Charlier coefficients was applied by analogy with the procedure described earlier [10]. Anisotropic displacement parameters for the H-atoms were calculated using the *SHADE*3 algorithm and inserted multiple times between the refinement steps until no further change was achieved [11]. The analysis of multipole-derived electron density was carried out in *WinXPRO*[12,13] by analogy with the published procedures[14,15]. The experimental results were compared with the theoretical ones calculated for the optimized free molecule; for this, the Multiwfn program was applied [15-17].

Deposition numbers CCDC 2220152 (**1**), 2220153 (**1**, multipole model), 2220154 (**2**), 2220155 (**3**), and 2220156 (**4**) contain supplementary crystallographic data for this paper. These data are provided free of charge by the joint Cambridge Crystallographic Data Centre and Fachinformationszentrum Karlsruhe Access Structures service www.ccdc.cam.ac.uk/structures.

Crystallographic data for **1**.

$C_{17}H_{15}CoN$, dark orange prism (0.412 × 0.212 × 0.140 mm^3), formula weight 292.23 g mol^{-1}; monoclinic, $P2_1/n$ (No. 14), a = 8.4801(5) Å, b = 17.2192(9) Å, c = 9.0543(5) Å, β = 106.975(2)°, V = 1264.51(12) Å^3, Z = 4, Z' = 1, T = 100.0(5) K, d_{calc} = 1.535 g cm^{-3}, μ(Mo Kα) = 1.339 mm^{-1}, F(000) = 604; T_{max}/T_{min} = 0.7626/0.4933; 319624 reflections were collected (2.366° ≤ θ ≤ 63.578°, index ranges: –20 ≤ h ≤ 21, –43 ≤ k ≤ 43 and –22 ≤ l ≤ 22), 20842 of which were unique, R_{int} = 0.0370, R_σ = 0.0181; completeness to θ of 63.578° 98.3 %. The refinement of 172 parameters with no restraints converged to $R1$ = 0.0292 and $wR2$ = 0.0759 for 18336 reflections with $I > 2\sigma(I)$ and

$R1$ = 0.0345 and $wR2$ = 0.0780 for all data with goodness-of-fit S = 1.148 and residual electron density ρ_{max}/ρ_{min} = 1.720 and –0.938 e Å^{-3}, rms 0.092 e Å^{-3}; max shift/e.s.d. in the last cycle 0.003. The following final agreement factors were obtained after multipole refinement against 19426 observed reflections $I > 1.5\sigma(I)$: R = 0.0196, wR = 0.0275, S = 1.009, and ρ_{max}/ρ_{min} = +0.218 and –0.272 e Å^{-3}, rms = 0.076 e Å^{-3}; max shift/e.s.d. in the last cycle –0.005.

Crystallographic data for **2**.

$C_{42}H_{46}N_2Ni_2O_2$, green prism (0.491 × 0.278 × 0.218 mm^3), formula weight 728.23 g mol^{-1}; triclinic, $P\overline{1}$ (No. 2), a = 12.6705(4) Å, b = 13.1789(4) Å, c = 13.4512(5) Å, α = 90.3632(6)°, β = 115.9550(6)°, γ = 117.9045(6)°, V = 1720.58(10) Å^3, Z = 2, Z' = ½ + ½ = 1, T = 100(2) K, d_{calc} = 1.406 g cm^{-3}, μ(Mo Kα) = 1.134 mm^{-1}, F(000) = 768; T_{max}/T_{min} = 0.7636/0.4492; 476867 reflections were collected (1.949° ≤ θ ≤ 47.381°, index ranges: –26 ≤ h ≤ 26, –27 ≤ k ≤ 27 and –27 ≤ l ≤ 27), 31721 of which were unique, R_{int} = 0.0747, R_σ = 0.0410; completeness to θ of 47.381° 99.1 %. The refinement of 479 parameters with 240 restraints converged to $R1$ = 0.0434 and $wR2$ = 0.1116 for 26703 reflections with $I > 2\sigma(I)$ and $R1$ = 0.0526 and $wR2$ = 0.1182 for all data with goodness-of-fit S = 1.074 and residual electron density ρ_{max}/ρ_{min} = 1.408 and –0.946 e Å^{-3}, rms 0.118 e Å^{-3}; max shift/e.s.d. in the last cycle 0.002.

Crystallographic data for **3**.

$C_{17}H_{15}BCoF_4N$, blue plank (0.281 × 0.223 × 0.078 mm^3), formula weight 379.04 g mol^{-1}; triclinic, $P1$ (No. 1), a = 8.6458(4) Å, b = 9.3663(4) Å, c = 10.7279(5) Å, α = 98.3482(14)°, β = 113.6046(13)°, γ = 102.7401(13)°, V = 749.49(6) Å^3, Z = 2, Z' = 2, T = 100(2) K, d_{calc} = 1.680 g cm^{-3}, μ(Mo Kα) = 1.187 mm^{-1}, F(000) = 384; T_{max}/T_{min} = 0.537827/0.008426; 90884 unique reflections were collected (2.310° ≤ θ ≤ 36.506°, index ranges: –14 ≤ h ≤ 14, –15 ≤ k ≤ 15 and –17 ≤ l ≤ 17), R_σ = 0.2994; completeness to θ of 36.506° 99.6 %. The refinement of 434 parameters with 3 restraints converged to $R1$ = 0.0661 and $wR2$ = 0.1603 for 54444 reflections with $I > 2\sigma$(I) and $R1$ = 0.0797 and $wR2$ = 0.1642 for all data with goodness-of-fit S = 1.095 and residual electron density ρ_{max}/ρ_{min} = 1.601 and –1.216 e Å^{-3}, rms 0.121 e Å^{-3}; max shift/e.s.d. in the last cycle 0.000. Absolute structure parameter is –0.081(9). Volume fraction of the minor twin component is 0.385(1).

Crystallographic data for **4**.

$C_{17}H_{15}MnN$, yellow prism (0.442 × 0.162 × 0.151 mm^3), formula weight 288.24 g mol^{-1}; orthorhombic, $P2_12_12_1$ (No. 19), a = 5.9934(2) Å, b = 7.8302(3) Å, c = 27.5628(8) Å, V =

1293.52(8) $Å^3$, $Z = 4$, $Z' = 1$, $T = 100(2)$ K, $d_{calc} = 1.480$ g cm^{-3}, μ(Mo $K\alpha$) = 1.003 mm^{-1}, $F(000) = 596$; $T_{max}/T_{min} = 1.000/0.515$; 18320 reflections were collected (2.704° ≤ θ ≤ 34.320°, index ranges: $-9 \le h \le 9$, $-12 \le k \le 11$, and $-41 \le l \le 43$), 4906 of which were unique, $R_{int} = 0.0292$, $R_\sigma = 0.0322$; completeness to θ of 34.320° 93.8 %. The refinement of 172 parameters with no restraints converged to $R1 = 0.0290$ and $wR2 = 0.0656$ for 4624 reflections with $I > 2\sigma(I)$ and $R1 = 0.0317$ and $wR2 = 0.0666$ for all data with goodness-of-fit $S = 1.053$ and residual electron density $\rho_{max}/\rho_{min} = 0.422$ and -0.414 e $Å^{-3}$, rms 0.070 e $Å^{-3}$; max shift/e.s.d. in the last cycle 0.001. Absolute structure parameter is 0.001(6).

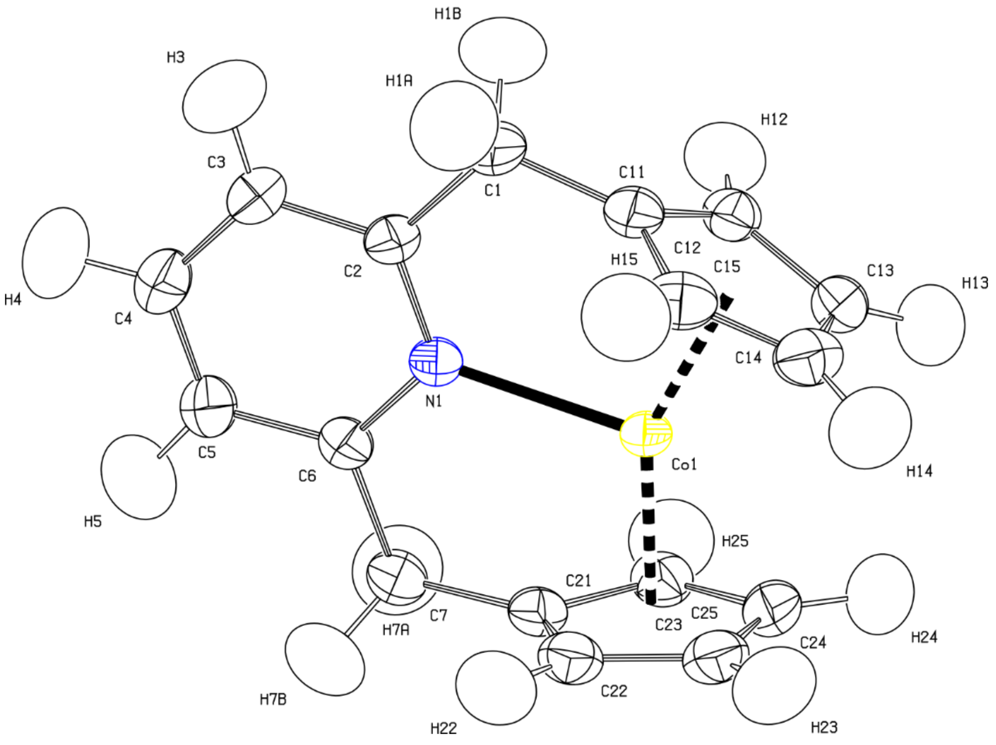

Supplementary Fig. 37.

ORTEP at 80 % probability anisotropic displacement ellipsoids for compound **1** according to high-resolution SC-XRD data (multipole model). Selected interatomic distances [Å]: Co1–N1 2.1998(3), Co1–C11 2.3292(2), Co1–C12 2.2971(2), Co1–C13 2.2783(3), Co1–C14 2.2893(3), Co1–C15 2.3186(2), Co1–C21 2.3194(2), Co1–C22 2.3115(2), Co1–C23 2.2985(2), Co1–C24 2.2849(2), Co1–C25 2.2940(2).

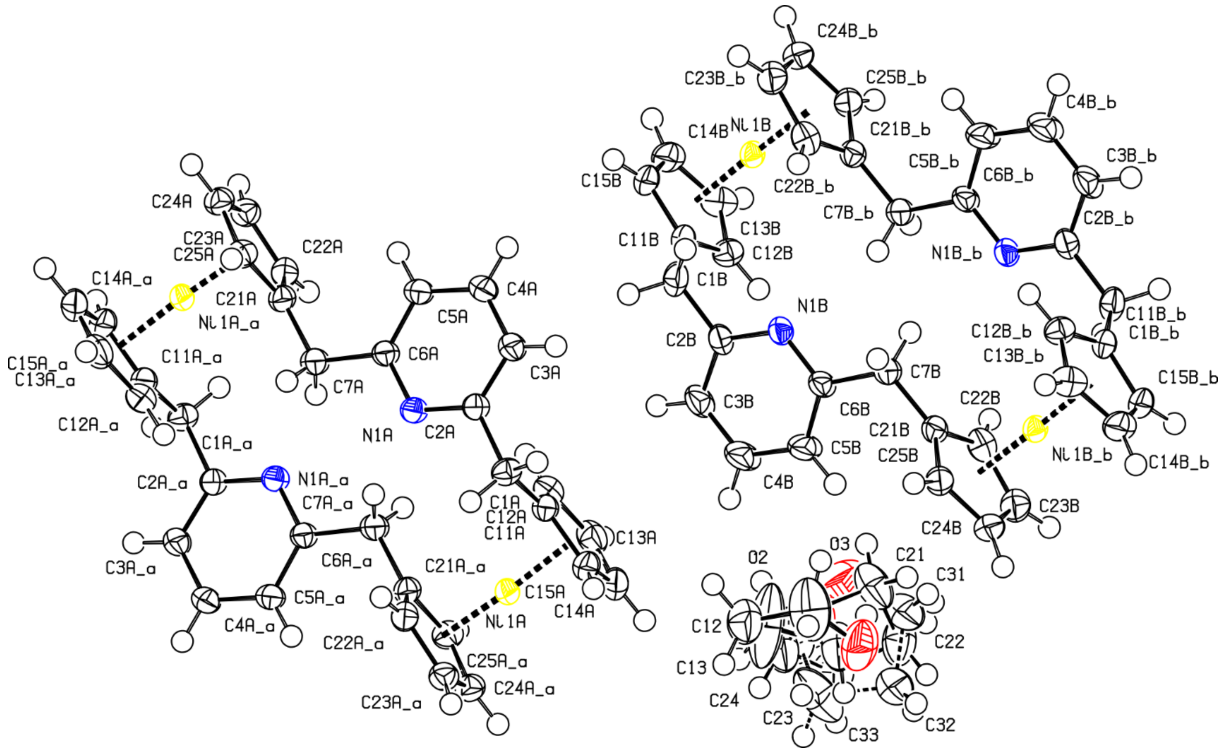

Supplementary Fig. 38.

ORTEP at 80 % probability anisotropic displacement ellipsoids of non-hydrogen atoms for compound **2** according to SC-XRD data. Selected interatomic distances [Å]: Ni1A–C11A 2.2242(7), Ni1A–C12A 2.1752(7), Ni1A–C13A 2.1466(8), Ni1A–C14A 2.1619(8), Ni1A–C15A 2.2024(7), Ni1A–C21A_a 2.1689(6), Ni1A–C22A_a 2.1601(7) Ni1A–C23A_a 2.1778(7), Ni1A–C24A_a 2.1962(7) Ni1A–C25A_a 2.1881(7), Ni1B–C11B 2.1939(7), Ni1B–C12B 2.1903(7), Ni1B C13B 2.1737(7), Ni1B–C14B 2.1690(7), Ni1B–C15B 2.1744(7), Ni1B–C21B_b 2.1706(6), Ni1B–C22B_b 2.1681(7), Ni1B–C23B_b 2.1812(8), Ni1B–C24B_b 2.1864(8), Ni1B–C25B_b 2.1771(7).

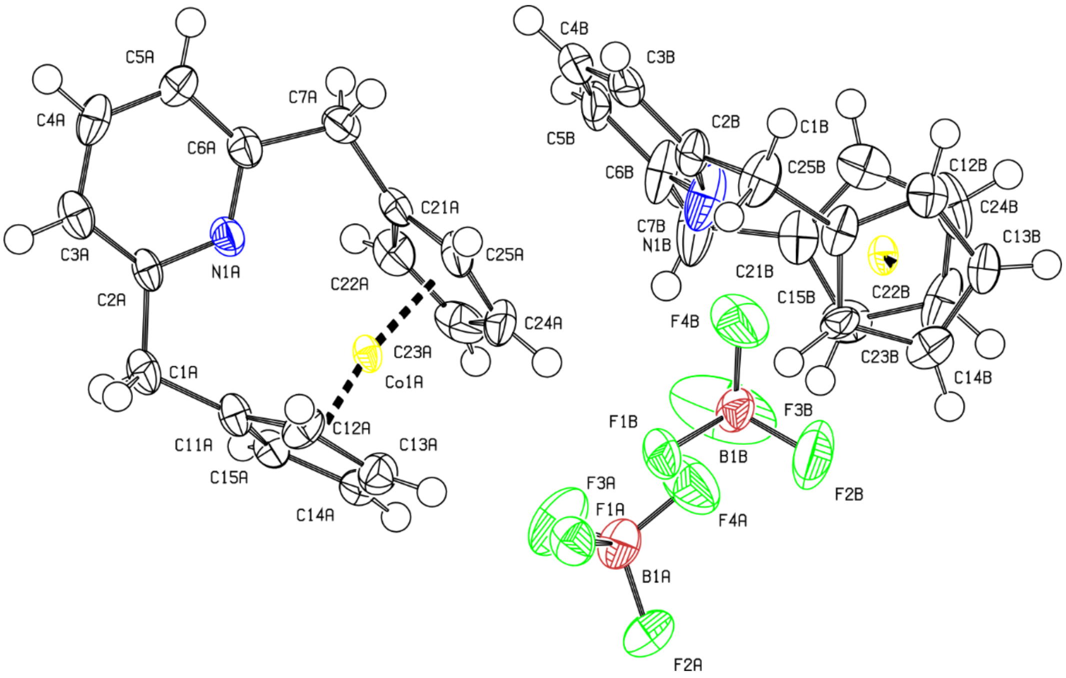

Supplementary Fig. 39.

ORTEP at 80 % probability anisotropic displacement ellipsoids of non-hydrogen atoms for compound **3** according to SC-XRD data. Selected interatomic distances [Å]: Co1A–C11A 2.221(5), Co1A–C12A 2.114(8), Co1A–C13A 2.017(6), Co1A–C14A 2.012(6), Co1A–C15A 2.102(6), Co1A–C21A 2.206(5), Co1A–C22A 2.099(6), Co1A–C23A 2.017(6), Co1A–C24A 2.011(6), Co1A–C25A 2.078(6), Co1B–C11B 2.197(5), Co1B–C12B 2.093(6), Co1B–C13B 2.017(5), Co1B–C14B 2.005(6), Co1B–C15B 2.081(7), Co1B–C21B 2.180(6), Co1B–C22B 2.066(6), Co1B–C23B 2.020(6), Co1B–C24B 2.037(8), Co1B–C25B 2.105(6), Co1A···N1A 2.745(4), Co1B···N1B 2.790(5).

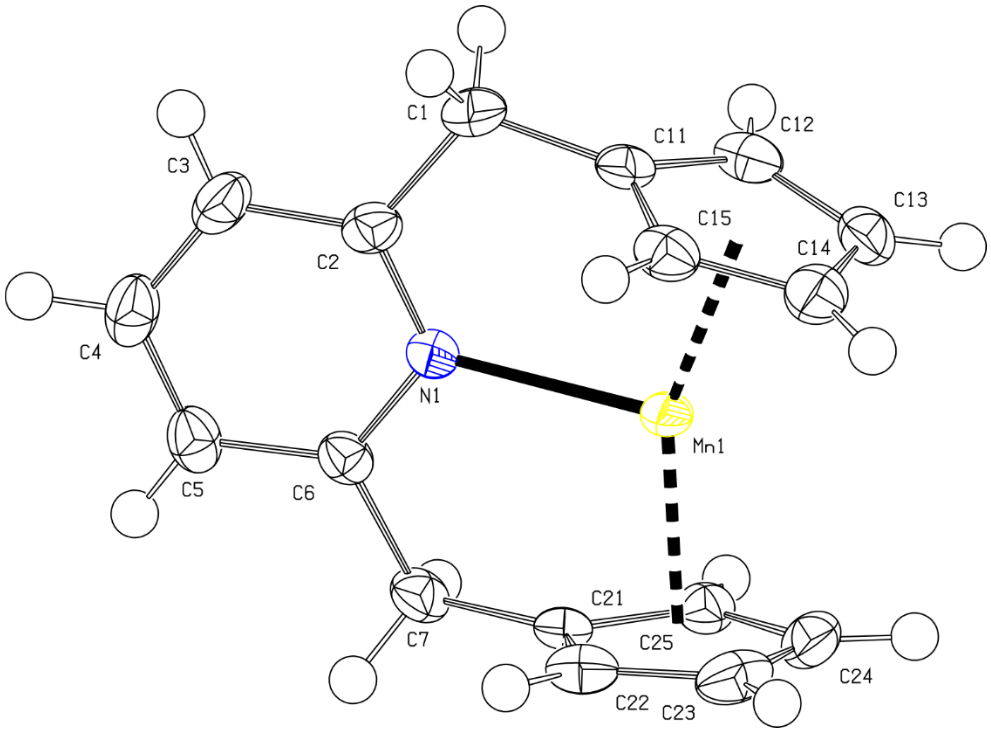

Supplementary Fig. 40.

ORTEP at 80 % probability anisotropic displacement ellipsoids of non-hydrogen atoms for compound **4** according to SC-XRD data. Selected interatomic distances [Å]: Mn1–N1 2.2709(14), Mn1–C11 2.4074(15), Mn1–C12 2.4524(15), Mn1–C13 2.4897(17), Mn1–C14 2.4547(17), Mn1–C15 2.3877(17), Mn1–C21 2.3737(16), Mn1–C22 2.4733(16), Mn1–C23 2.5559(17), Mn1–C24 2.4936(18), Mn1–C25 2.3643(17).

5. DFT studies

Test Computations.

Adding two electrons to the 19 electron configuration of cobaltocene gives either S = 1/2 or S = 3/2. Therefore, we initially optimized the molecular structure derived from SC-XRD in the doublet and quartet state. We considered a diverse set of dispersion-corrected (D4 unless otherwise noted) DFT levels such as GFN2-xTB [18,19], B97-3c [20], BP86 [21-23], TPSS, M06-L [24,25] (using D3) [26], TPSSh [27-29], and PBE0 [30,31]. Optimizations in the doublet state result in scission of the Co−N bond at any level to afford a local minimum structure with formal 19-electrons (**1’**). In the quartet state, the 21 valence electron complex with a Co−N bond is the minimum structure. The computed Co−Cp_{cent} bond lengths agree favorably with those obtained by single crystal X-ray diffraction at all levels of theory (Supplementary Fig. 41A). However, for some functionals, the optimized Co−N bond differs notably from the experimentally determined one (Supplementary Fig. 41A). The influence of scalar-relativistic effects on the bond lengths is minor and BP86 and TPSS perform best. We, therefore, considered the non-relativistic TPSS level to be suitable for structure optimizations. Then, we carried out single-point energy computations to calculate the Gibbs free energy difference between the isomers **1** (S = 3/2, formal 21-electrons) and **1’** (S = 1/2, formal 19-electrons). At the TPSS level, **1’** is 3.0/1.9 kcal/mol lower in energy at the scalar-relativistic/non-relativistic level, respectively. However, at the TPSSh level, **1** is favored by 3.8/5.0 kcal/mol at the scalar-relativistic/non-relativistic level, respectively. Therefore, we used the non-relativistic TPSSh-D4/def2-QZVPP//TPSS-D4/def2-TZVPP level of theory for computations of the electronic structure of **1** and **1’**.

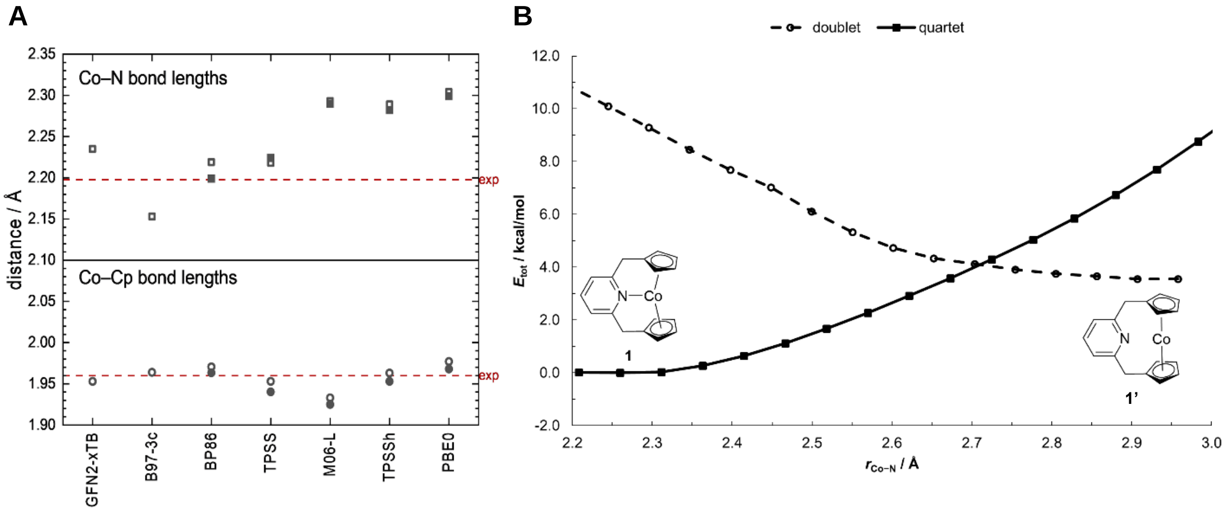

Supplementary Fig. 41.

(**A**) Comparison between computed and experimental bond lengths determined by SCXRD. Hollow and filled symbols refer to non-relativistic and scalar-relativistic (ZORA) computations. The (ZORA-)BP86 and ZORA-TPSS optimized structures are distorted from C_2 symmetry and the given values were averaged. BP86, TPSS, M06-L, TPSSh, PBE0 were used in combination with the def2-TZVPP basis set and are dispersion-corrected (D4 except for M06-L using D3). (**B**) Relaxed potential energy surface scan of the Co−N bond at the TPSS-D4/def2-TZVPP level with subsequent single-point energies at the TPSSh-D4/def2-QZVPP level.

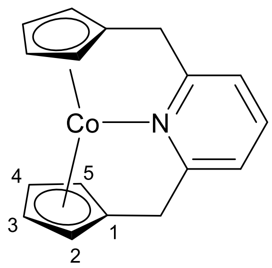

Supplementary Table 3.

Computed delocalization indices (DIs), Wiberg bond indices (WBIs), natural charges with summed hydrogen atoms (charge), and natural spin densities (spin density) at the TPSSh-D4/def2-QZVPP//TPSS-D4/def2-TZVPP level of theory.

	Co bound to		Totals by atom		
	DI[a]	WBI[b]	WBI	Charge[c]	Spin Density
Co	6.39		1.85	0.96	2.09
N	0.74	0.23	3.24	−0.49	0.09
C1	0.47	0.16	4.01	−0.13	0.14
C2	0.51	0.13	3.95	−0.11	0.04
C3	0.53	0.17	3.95	−0.08	0.11
C4	0.54	0.15	3.95	−0.08	0.09
C5	0.53	0.15	3.95	−0.12	0.04

[a] remaining DIs < 0.08. [b] For comparison, the WBI of Co−C in cobaltocene is 0.22(5). [c] The net charge attributed to the entire Cp and pyridine parts is −0.52 and +0.01, respectively.

The positive charge of the cobalt is neutralized mainly by the negatively charged Cp parts, while the pyridine part is neutrally charged. The charge distribution over the Cp ligands is uniform (−0.08 to −0.13 for the individual C atoms with summed H atoms).

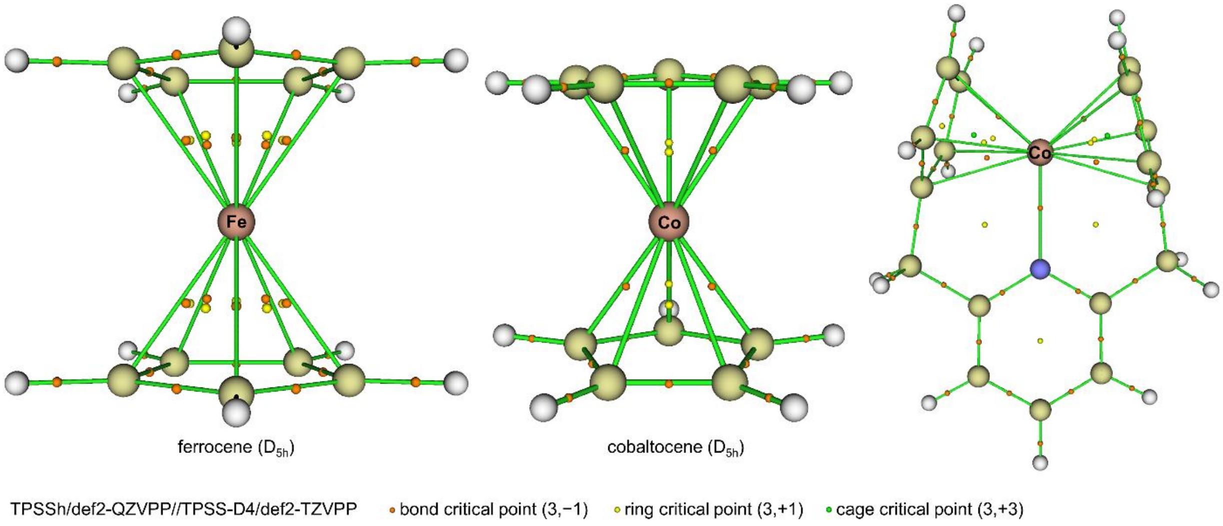

Supplementary Fig. 42.
Comparison of computed critical points for ferrocene, cobaltocene, and **1**.

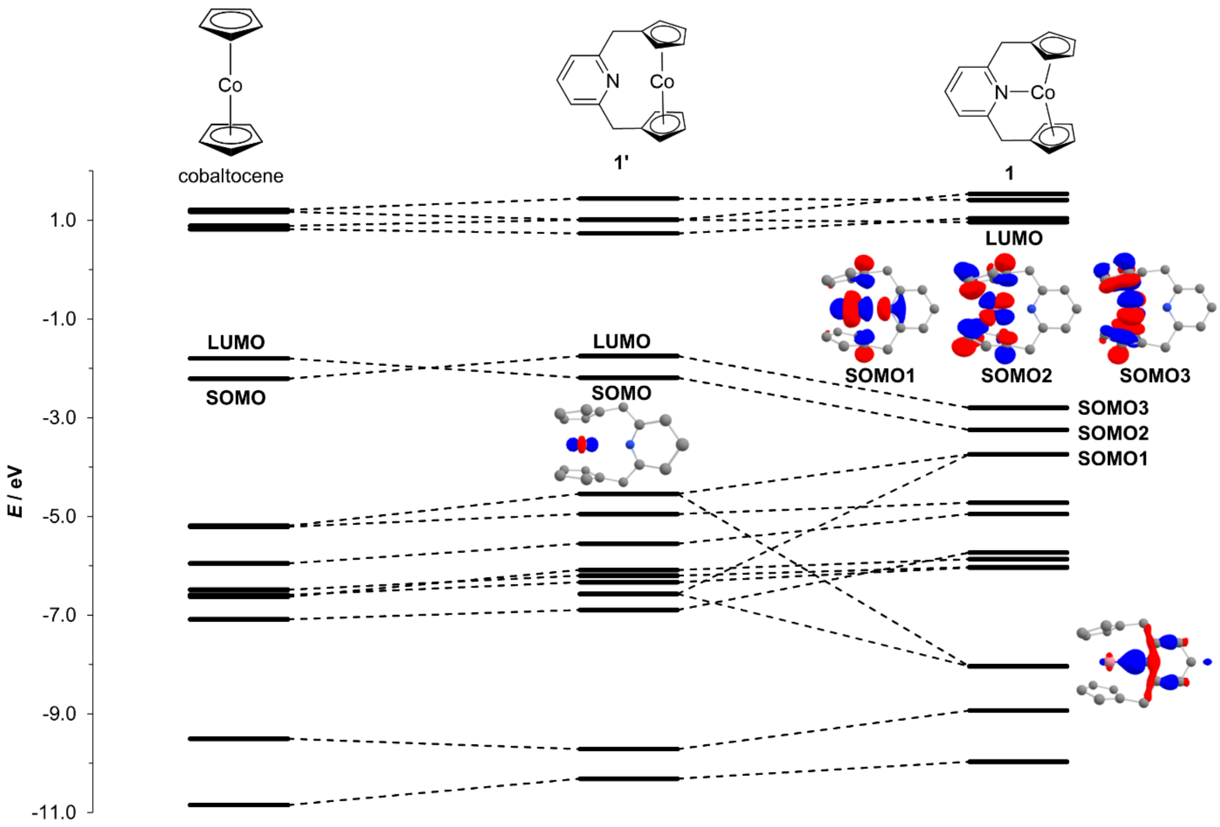

Supplementary Fig. 43.

Comparison of MO diagram of cobaltocene, **1'**, and **1** derived from canonical KS-QROs computed at TPSSh-D4/def2-QZVPP//TPSS-D4/def2-TZVPP level. Orbital occupation is not depicted for clarity (all MOs up to the SOMO level are doubly occupied, the SOMOs themselves are singly occupied, and MOs equal to/beyond the LUMOs are unoccupied).

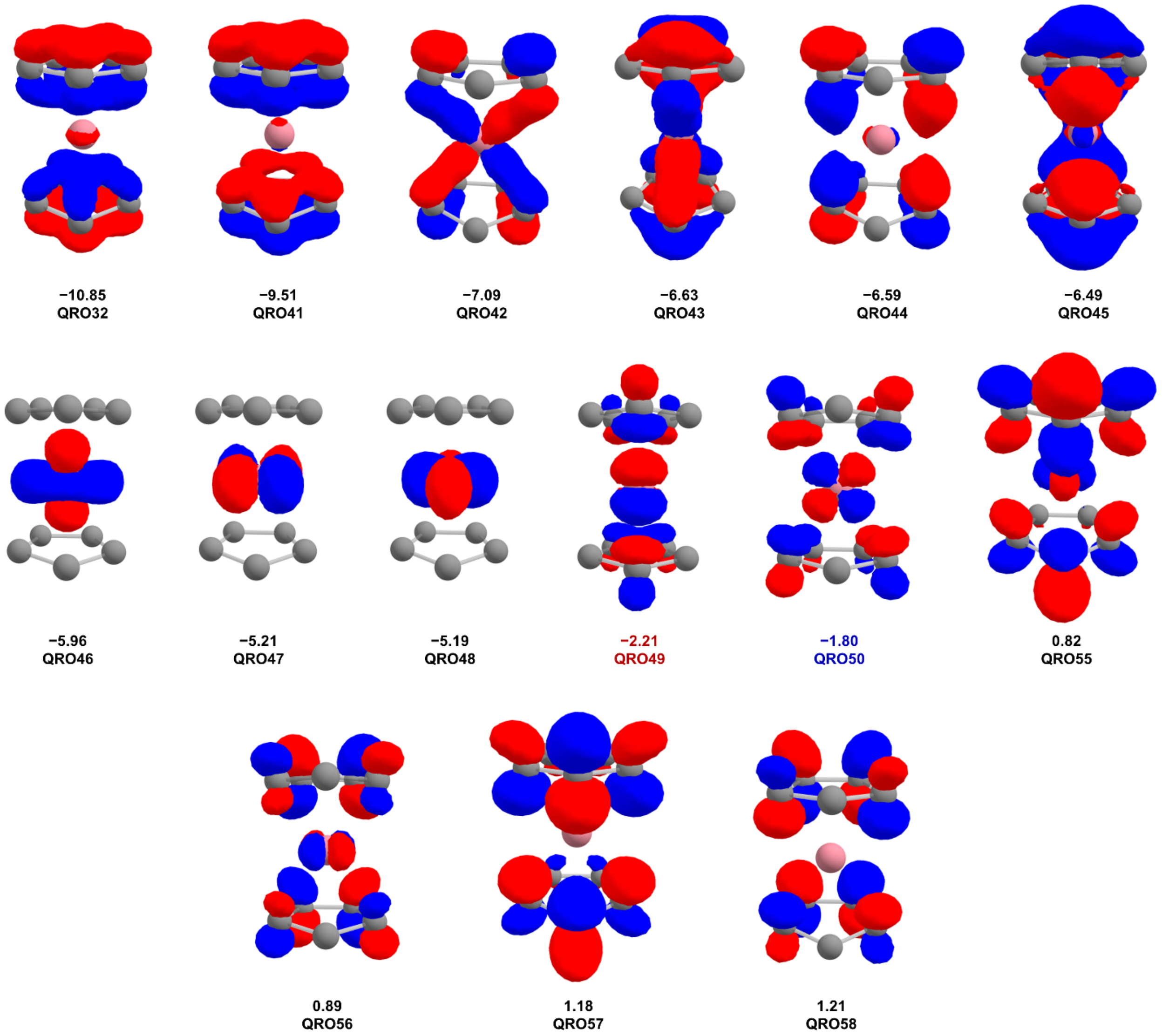

Supplementary Fig. 44.

List of considered canonical KS-QROs of cobaltocene (isovalues 0.05-0.07). All energies (in eV) refer to TPSSh-D4/def2-QZVPP//TPSS-D4/def2-TZVPP level of theory. SOMO and LUMO levels are highlighted in red and blue, respectively.

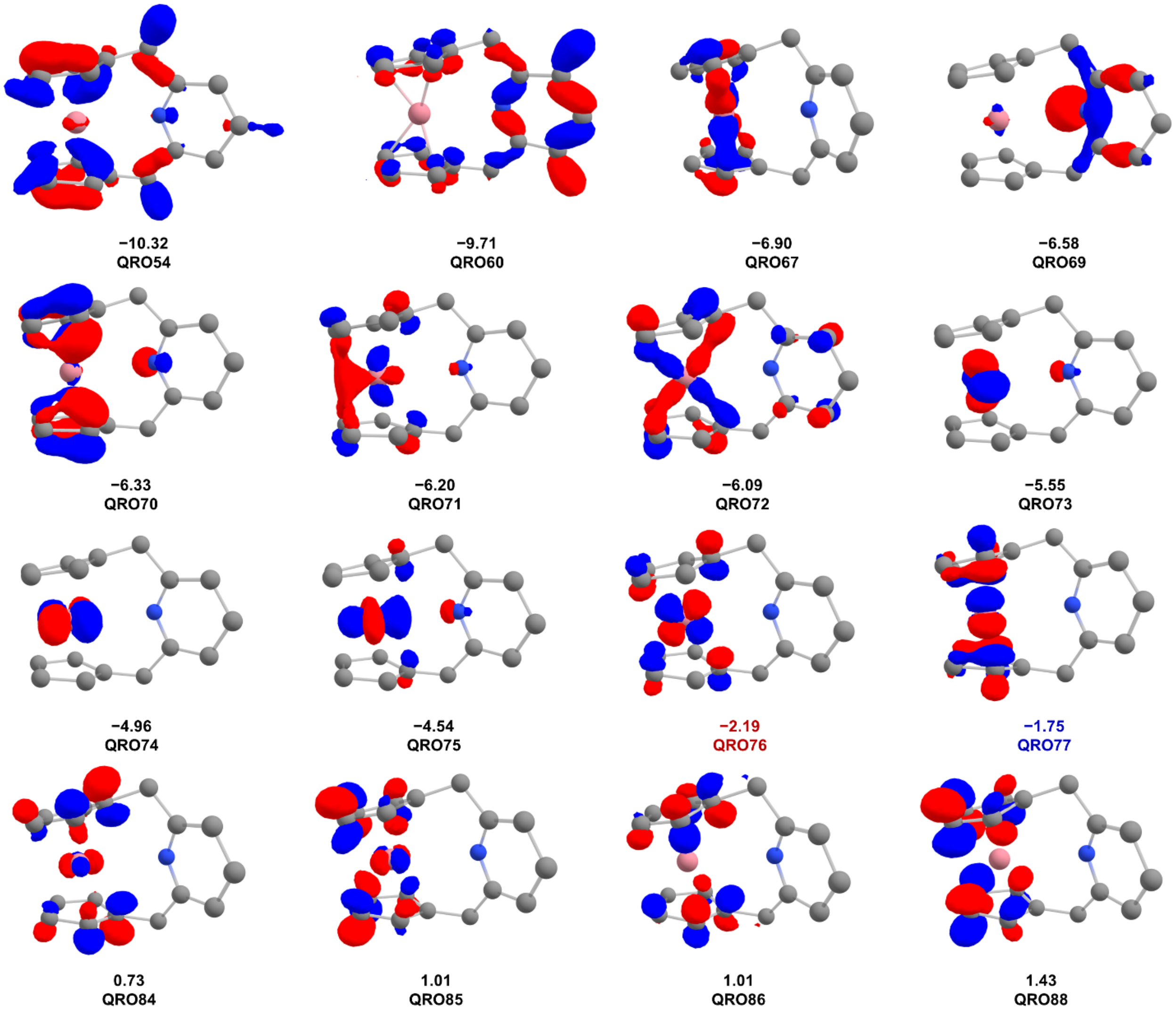

Supplementary Fig. 45.

List of considered canonical KS-QROs of **1’** (isovalues 0.05-0.07). All energies (in eV) refer to TPSSh-D4/def2-QZVPP//TPSS-D4/def2-TZVPP level of theory. SOMO and LUMO levels are highlighted in red and blue, respectively.

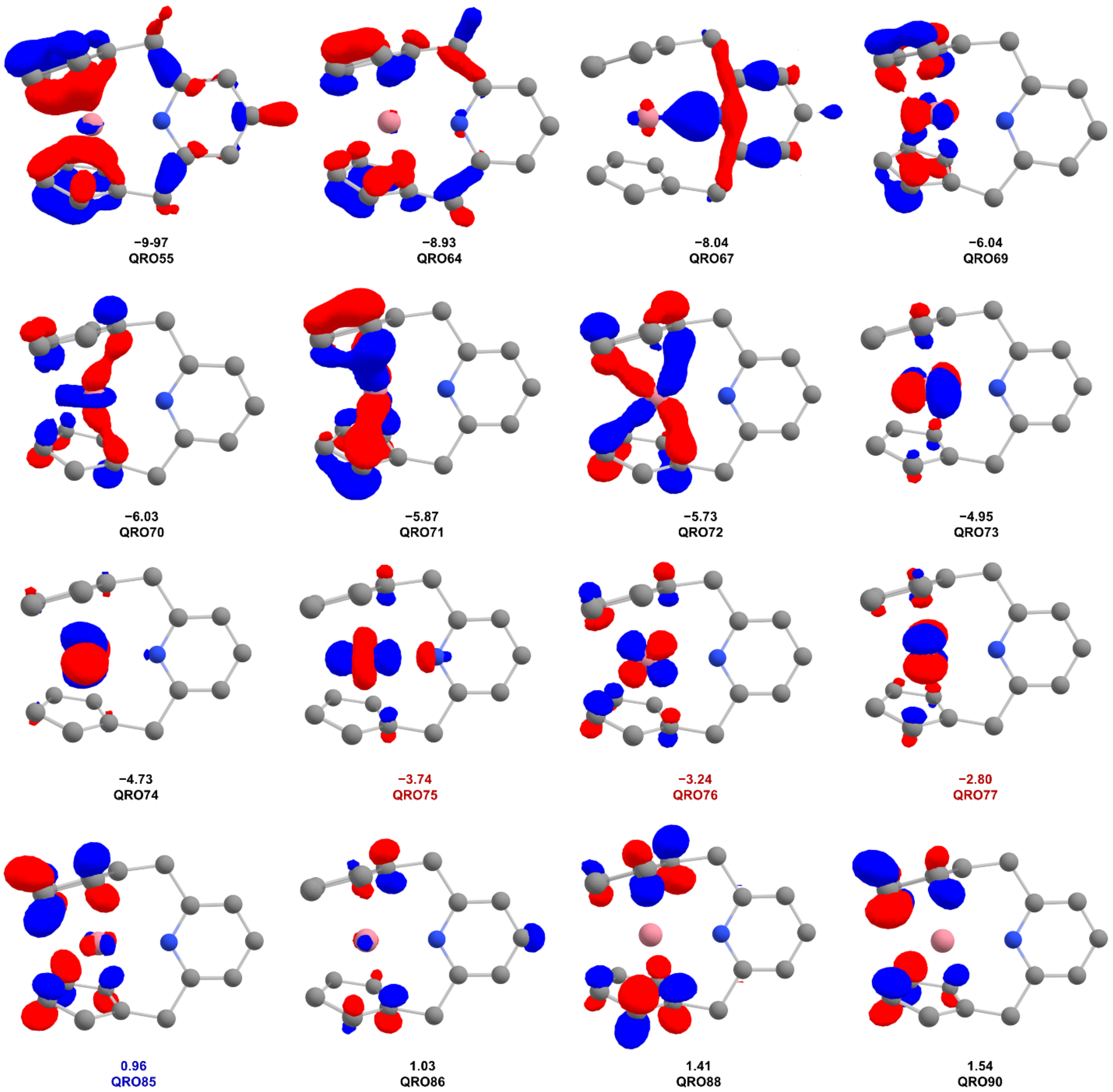

Supplementary Fig. 46.

List of considered canonical KS-QROs of **1** (isovalues 0.05-0.07). All energies (in eV) refer to TPSSh-D4/def2-QZVPP//TPSS-D4/def2-TZVPP level of theory. SOMO and LUMO levels are highlighted in red and blue, respectively.

Atomic Coordinates and Energies

1 (*S* = 3/2, TPSS-D4/def2-TZVPP)

Co	0.000000000	0.000000000	-0.935251000
N	0.000000000	0.000000000	1.282319000
C	1.143916000	-0.207321000	1.972294000
C	2.211065000	-0.196449000	-0.283849000
C	-2.001603000	-1.092628000	-0.863246000
H	-2.107618000	-2.041841000	-0.354768000
C	1.625521000	-0.500844000	-2.480821000
H	1.378658000	-0.966227000	-3.425126000
C	-2.211065000	0.196449000	-0.283849000
C	1.175946000	-0.206057000	3.365862000
H	2.119920000	-0.371316000	3.876559000
C	1.965516000	-1.178373000	-1.279549000
H	2.028412000	-2.249304000	-1.139009000
C	1.646986000	0.899526000	-2.222576000
H	1.428507000	1.679755000	-2.938780000
C	-1.143916000	0.207321000	1.972294000
C	2.001603000	1.092628000	-0.863246000
H	2.107618000	2.041841000	-0.354768000
C	-1.646986000	-0.899526000	-2.222576000
H	-1.428508000	-1.679755000	-2.938780000
C	-1.965516000	1.178373000	-1.279549000
H	-2.028412000	2.249304000	-1.139009000
C	2.407733000	-0.461125000	1.171653000
H	3.209450000	0.161894000	1.588648000
H	2.712670000	-1.503180000	1.331117000
C	0.000000000	0.000000000	4.077822000
H	0.000000000	0.000000000	5.163798000
C	-1.175946000	0.206057000	3.365862000
H	-2.119920000	0.371316000	3.876559000
C	-1.625521000	0.500844000	-2.480821000
H	-1.378658000	0.966227000	-3.425126000
C	-2.407733000	0.461125000	1.171653000
H	-3.209450000	-0.161894000	1.588648000
H	-2.712670000	1.503180000	1.331117000

E	-2094.916099124146
E [TPSS-D4/def2-QZVPP]	-2094.981507853812
E [TPSSh-D4/def2-QZVPP]	-2094.867629341072
ZPVE	0.26572184
E corr	0.28200563

H corr	0.28294984
G corr	0.22270417

1’ (*S* = 1/2, TPSS-D4/def2-TZVPP)

Co	-0.545575000	1.069513000	0.000000000
N	0.100917000	-1.827089000	0.000000000
C	0.728305000	-2.040814000	1.154648000
C	-0.328899000	0.018179000	1.982889000
C	0.620222000	1.063049000	-1.746762000
H	1.696978000	0.949498000	-1.773321000
C	-0.078980000	2.316332000	1.621574000
H	0.376718000	3.287746000	1.489163000
C	-0.328899000	0.018179000	-1.982888000
C	2.000560000	-2.614666000	1.206726000
H	2.498731000	-2.780622000	2.157420000
C	0.620222000	1.063049000	1.746762000
H	1.696977000	0.949499000	1.773322000
C	-1.460007000	2.025142000	1.614545000
H	-2.267762000	2.733169000	1.489594000
C	0.728305000	-2.040815000	-1.154648000
C	-1.606381000	0.595514000	1.774170000
H	-2.548069000	0.062975000	1.813548000
C	-0.078979000	2.316332000	-1.621573000
H	0.376719000	3.287746000	-1.489163000
C	-1.606381000	0.595514000	-1.774170000
H	-2.548069000	0.062975000	-1.813548000
C	-0.013274000	-1.419662000	2.317629000
H	-0.950479000	-1.960417000	2.489509000
H	0.579155000	-1.481820000	3.237914000
C	2.627481000	-2.930163000	0.000000000
H	3.620051000	-3.371677000	0.000000000
C	2.000560000	-2.614666000	-1.206726000
H	2.498731000	-2.780623000	-2.157420000
C	-1.460007000	2.025142000	-1.614545000
H	-2.267761000	2.733169000	-1.489594000
C	-0.013274000	-1.419662000	-2.317629000
H	0.579155000	-1.481820000	-3.237914000
H	-0.950480000	-1.960417000	-2.489509000

E	-2094.921124683756
E [TPSS-D4/def2-QZVPP]	-2094.986819876332
E [TPSSh-D4/def2-QZVPP]	-2094.861997515120

ZPVE	0.26617832
E corr	0.28182702
H corr	0.28277123
G corr	0.22501506

Cobaltocene

Co	0.002057000	0.000000000	0.000000000
C	-0.995117000	0.705111000	1.719649000
C	-0.995117000	0.705111000	-1.719649000
H	-1.863452000	1.348789000	-1.704217000
C	0.381255000	-1.143409000	1.678866000
H	0.708559000	-2.174367000	1.656581000
C	0.381255000	1.143409000	-1.678866000
C	-0.995117000	-0.705111000	1.719649000
H	-1.863452000	-1.348789000	1.704216000
C	1.228043000	0.000000000	1.744128000
H	2.308440000	0.000000000	1.732076000
C	0.381255000	1.143409000	1.678866000
H	0.708559000	2.174367000	1.656581000
C	-0.995117000	-0.705111000	-1.719649000
H	-1.863452000	-1.348789000	-1.704216000
C	1.228043000	0.000000000	-1.744128000
H	2.308440000	0.000000000	-1.732076000
H	-1.863452000	1.348789000	1.704216000
C	0.381255000	-1.143409000	-1.678866000
H	0.708559000	-2.174367000	-1.656581000
H	0.708559000	2.174367000	-1.656581000

6. Supplementary references

1 Paolucci, G. *et al.* New dinuclear bis(cyclopentadienyl)lanthanoid chlorides containing η^5-C_5H_4 ligands linked by a metal-coordinated 2,6-dimethylenepyridyl unit. *J. Organomet. Chem.* **471**, 97-104, doi: 10.1016/0022-328X(94)88112-X (1994).

2 Epel, B., Gromov, I., Stoll, S., Schweiger, A. & Goldfarb, D. Spectrometer manager: A versatile control software for pulse EPR spectrometers. *Concepts Magn. Reson. B: Magn. Reson. Eng.* **26B**, 36-45, doi: 10.1002/cmr.b.20037 (2005).

3 Stoll, S. & Schweiger, A. EasySpin, a comprehensive software package for spectral simulation and analysis in EPR. *J. Magn. Reson.* **178**, 42-55, doi: 10.1016/j.jmr.2005.08.013 (2006).

4 Stoll, S. & Britt, R. D. General and efficient simulation of pulse EPR spectra. *Phys. Chem. Chem. Phys.* **11**, 6614-6625, doi:10.1039/B907277B (2009).

5 Sheldrick, G. SHELXT - Integrated space-group and crystal-structure determination. *Acta Crystallogr. A* **71**, 3-8, doi:doi:10.1107/S2053273314026370 (2015).

6 Sheldrick, G. Crystal structure refinement with SHELXL. *Acta Crystallogr. C* **71**, 3-8, doi:doi:10.1107/S2053229614024218 (2015).

7 Farrugia, L. J. WinGX and ORTEP for Windows: an update. *J. Appl. Crystallogr.* **45**, 849-854, doi: 10.1107/S0021889812029111 (2012).

8 Jelsch, C., Guillot, B., Lagoutte, A. & Lecomte, C. Advances in protein and small-molecule charge-density refinement methods using MoPro. *J. Appl. Crystallogr.* **38**, 38-54, doi:doi:10.1107/S0021889804025518 (2005).

9 Su, Z. & Coppens, P. Relativistic X-ray elastic scattering factors for neutral atoms Z = 1–54 from multiconfiguration Dirac–Fock wavefunctions in the 0–12$Å^{-1}$ sinθ/λ range, and six-Gaussian analytical expressions in the 0–6$Å^{-1}$ range. Erratum. *Acta Crystallogr. A* **54**, 357-357, doi: 10.1107/S010876739800124X (1998).

10 Shteingolts, S. A. *et al.* On the transfer of theoretical multipole parameters for restoring static electron density and revealing and treating atomic anharmonic motion. Features of chemical bonding in crystals of an isocyanuric acid derivative. *Acta Crystallogr. B* **77**, 871-891, doi:doi:10.1107/S2052520621009690 (2021).

11 Madsen, A. SHADE web server for estimation of hydrogen anisotropic displacement parameters. *J. Appl. Crystallogr.* **39**, 757-758, doi:doi:10.1107/S0021889806026379 (2006).

12 Stash, A. I. & Tsirelson, V. G. Developing WinXPRO: a software for determination of the multipole-model-based properties of crystals. *J. Appl. Crystallogr.* **47**, 2086-2089, doi:doi:10.1107/S1600576714021566 (2014).

13 Stash, A. I. & Tsirelson, V. G. WinXPRO, 3DPlot and TrajPlot computer software: new options for orbital-free quantum crystallography studies. *J. Appl. Crystallogr.* **55**, 420-424, doi:doi:10.1107/S1600576722002321 (2022).

14 Shteingolts, S. A., Stash, A. I., Tsirelson, V. G. & Fayzullin, R. R. Real-Space Interpretation of Interatomic Charge Transfer and Electron Exchange Effects by Combining Static and Kinetic Potentials and Associated Vector Fields. *Chem. Eur. J.* **28**, e202200985, doi: 10.1002/chem.202200985 (2022).

15 Kartashov, S. V., Shteingolts, S. A., Stash, A. I., Tsirelson, V. G. & Fayzullin, R. R. Electronic and Crystal Packing Effects in Terms of Static and Kinetic Force Field

Features: Picolinic Acid N-Oxide and Methimazole. *Cryst. Growth Des.* **23**, 1726-1742, doi:10.1021/acs.cgd.2c01286 (2023).
16 Lu, T. & Chen, F. Multiwfn: A multifunctional wavefunction analyzer. *J. Comput. Chem.* **33**, 580-592, doi: 10.1002/jcc.22885 (2012).
17 Zhang, J. & Lu, T. Efficient evaluation of electrostatic potential with computerized optimized code. *Phys. Chem. Chem. Phys.* **23**, 20323-20328, doi:10.1039/D1CP02805G (2021).
18 Grimme, S., Bannwarth, C. & Shushkov, P. A Robust and Accurate Tight-Binding Quantum Chemical Method for Structures, Vibrational Frequencies, and Noncovalent Interactions of Large Molecular Systems Parametrized for All spd-Block Elements (Z = 1–86). *J. Chem. Theory Comput.* **13**, 1989-2009, doi:10.1021/acs.jctc.7b00118 (2017).
19 Bannwarth, C., Ehlert, S. & Grimme, S. GFN2-xTB—An Accurate and Broadly Parametrized Self-Consistent Tight-Binding Quantum Chemical Method with Multipole Electrostatics and Density-Dependent Dispersion Contributions. *J. Chem. Theory Comput.* **15**, 1652-1671, doi:10.1021/acs.jctc.8b01176 (2019).
20 Brandenburg, J. G., Bannwarth, C., Hansen, A. & Grimme, S. B97-3c: A revised low-cost variant of the B97-D density functional method. *J. Chem. Phys.* **148**, 064104, doi:10.1063/1.5012601 (2018).
21 Becke, A. D. Density-functional exchange-energy approximation with correct asymptotic behavior. *Phys. Rev. A* **38**, 3098-3100, doi:10.1103/PhysRevA.38.3098 (1988).
22 Perdew, J. P. Density-functional approximation for the correlation energy of the inhomogeneous electron gas. *Phys. Rev. B* **33**, 8822-8824, doi:10.1103/PhysRevB.33.8822 (1986).
23 Perdew, J. P. Erratum: Density-functional approximation for the correlation energy of the inhomogeneous electron gas. *Phys.l Rev. B* **34**, 7406-7406, doi:10.1103/PhysRevB.34.7406 (1986).
24 Zhao, Y. & Truhlar, D. G. A new local density functional for main-group thermochemistry, transition metal bonding, thermochemical kinetics, and noncovalent interactions. *J. Chem. Phys.* **125**, 194101, doi:10.1063/1.2370993 (2006).
25 Zhao, Y. & Truhlar, D. G. The M06 suite of density functionals for main group thermochemistry, thermochemical kinetics, noncovalent interactions, excited states, and transition elements: two new functionals and systematic testing of four M06-class functionals and 12 other functionals. *Theor. Chem. Acc.* **120**, 215-241, doi:10.1007/s00214-007-0310-x (2008).
26 Grimme, S., Antony, J., Ehrlich, S. & Krieg, H. A consistent and accurate ab initio parametrization of density functional dispersion correction (DFT-D) for the 94 elements H-Pu. *J. Chem. Phys.* **132**, 154104, doi:10.1063/1.3382344 (2010).
27 Tao, J., Perdew, J. P., Staroverov, V. N. & Scuseria, G. E. Climbing the Density Functional Ladder: Nonempirical Meta--Generalized Gradient Approximation Designed for Molecules and Solids. *Phys. Rev. Lett.* **91**, 146401, doi:10.1103/PhysRevLett.91.146401 (2003).
28 Staroverov, V. N., Scuseria, G. E., Tao, J. & Perdew, J. P. Comparative assessment of a new nonempirical density functional: Molecules and hydrogen-bonded complexes. *J. Chem. Phys.* **119**, 12129-12137, doi:10.1063/1.1626543 (2003).
29 Staroverov, V. N., Scuseria, G. E., Tao, J. & Perdew, J. P. Erratum: “Comparative assessment of a new nonempirical density functional: Molecules and hydrogen-bonded

complexes" [J. Chem. Phys. 119, 12129 (2003)]. *J. Chem. Phys.* **121**, 11507-11507, doi:10.1063/1.1795692 (2004).
30 Adamo, C. & Barone, V. Toward reliable density functional methods without adjustable parameters: The PBE0 model. *J. Chem. Phys.* **110**, 6158-6170, doi:10.1063/1.478522 (1999).
31 Ernzerhof, M. & Scuseria, G. E. Assessment of the Perdew–Burke–Ernzerhof exchange-correlation functional. *J. Chem. Phys.* **110**, 5029-5036, doi:10.1063/1.478401 (1999).
